# The influence of tree genus, phylogeny, and richness on the specificity, rarity, and diversity of ectomycorrhizal fungi

**DOI:** 10.1111/1758-2229.13253

**Published:** 2024-04-04

**Authors:** Leho Tedersoo, Rein Drenkhan, Kessy Abarenkov, Sten Anslan, Mohammad Bahram, Kriss Bitenieks, Franz Buegger, Daniyal Gohar, Niloufar Hagh‐Doust, Darta Klavina, Kristaps Makovskis, Austra Zusevica, Karin Pritsch, Allar Padari, Sergei Põlme, Saleh Rahimlou, Dainis Rungis, Vladimir Mikryukov

**Affiliations:** ^1^ Mycology and Microbiology Center University of Tartu Tartu Estonia; ^2^ Institute of Ecology and Earth Sciences University of Tartu Tartu Estonia; ^3^ College of Science King Saud University Riyadh Saudi Arabia; ^4^ Institute of Forestry and Engineering Estonian University of Life Sciences Tartu Estonia; ^5^ Natural History Museum University of Tartu Tartu Estonia; ^6^ Department of Ecology Swedish University of Agricultural Sciences Uppsala Sweden; ^7^ Latvian State Forest Research Institute ‘Silava’ (LSFRI Silava) Salaspils Latvia; ^8^ Helmholtz Zentrum München – German Research Center for Environmental Health (GmbH), Research Unit Environmental Simulation Neuherberg Germany

## Abstract

Partner specificity is a well‐documented phenomenon in biotic interactions, yet the factors that determine specificity in plant‐fungal associations remain largely unknown. By utilizing composite soil samples, we identified the predictors that drive partner specificity in both plants and fungi, with a particular focus on ectomycorrhizal associations. Fungal guilds exhibited significant differences in overall partner preference and avoidance, richness, and specificity to specific tree genera. The highest level of specificity was observed in root endophytic and ectomycorrhizal associations, while the lowest was found in arbuscular mycorrhizal associations. The majority of ectomycorrhizal fungal species showed a preference for one of their partner trees, primarily at the plant genus level. Specialist ectomycorrhizal fungi were dominant in belowground communities in terms of species richness and relative abundance. Moreover, all tree genera (and occasionally species) demonstrated a preference for certain fungal groups. Partner specificity was not related to the rarity of fungi or plants or environmental conditions, except for soil pH. Depending on the partner tree genus, specific fungi became more prevalent and relatively more abundant with increasing stand age, tree dominance, and soil pH conditions optimal for the partner tree genus. The richness of partner tree species and increased evenness of ectomycorrhizal fungi in multi‐host communities enhanced the species richness of ectomycorrhizal fungi. However, it was primarily the partner‐generalist fungi that contributed to the high diversity of ectomycorrhizal fungi in mixed forests.

## Graphical abstract

More than half of ectomycorrhizal fungi display partner specificity that is determined by tree genus and among‐plant phylogenetic relationships. Specific fungi are more common in monospecific stands and at extreme soil pH. Rarity is unrelated to specificity, but it is driven by overall fungal and plant partner richness, certain tree species (*Pinus* and *Salix*) and moderately acidic soils. Mainly partner‐generalist fungi contribute to the high diversity of ectomycorrhizal fungi in mixed forests.

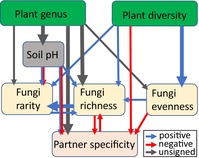

## INTRODUCTION

Host specificity is an important factor driving biodiversity and distribution of microorganisms besides abiotic environmental predictors such as pH and climate (Mallott & Amato, 2021; Perret et al., 2000). Specificity for living or dead hosts is particularly important in the fungal kingdom, which comprises ecologically important decomposers, pathogens and mycorrhizal root symbionts (Kennedy et al., 2015; Leonhardt et al., 2019; Põlme et al., 2018). Ectomycorrhizal (EcM) symbiosis is ecologically and economically the dominant type of root symbiosis of trees in much of the boreal and temperate forests of the Northern Hemisphere (Soudzilovskaia et al., 2019). Understanding of host specificity in trees and fungi provides important insights into their ecology and co‐evolution as well as practical information for reforestation programmes (Kennedy et al., 2015; Krpata et al., 2007). In this paper, we refer to host specificity as *partner specificity* to indicate that specificity and nutrient exchange should be viewed from the perspectives of both interacting organisms in mycorrhizal symbiosis (Southworth et al., 2005; Wyatt et al., 2014).

Partner specificity in ectomycorrhizal symbiosis is typically measured based on molecular identification of fungi from root tips that are traced to the plant individuals in mixed plant communities (Horton & Bruns, 1998; Ishida et al., 2007). Alternatively, specificity patterns can be addressed using sequencing of fungi from root tips (van der Linde et al., 2018) or soil environmental DNA (van Galen et al., 2023; Weißbecker et al., 2019) in replicated monoculture stands, or based on expert knowledge about plant‐macrofungi associations (Taudiere et al., 2015). Relative specificity is typically addressed using binomial tests (such as chi‐square test, Fisher's exact test or analogues) for fungal species, multivariate tests (perMANOVA and ordination methods) for plants and bipartite networks for both groups of partners. However, these tests offer only qualitative insights into specificity and/or cannot ascribe the effect to a particular partner taxon. Because relative specificity is a continuous variable (Ishida et al., 2007), it can be quantified using an indicator species analysis approach (e.g., Weißbecker et al., 2019; Yildirim et al., 2014) that was originally developed for determining habitat specialist plants (De Caceres & Legendre, 2009). Out of the multiple indicator indices used for inferring habitat specificity, the Φ index (also known as phi‐coefficient of association) has multiple benefits, such as range from −1 to 1, symmetry and index value calculation for all habitat types, and it has been recommended for determining diagnostic species in plant communities (Trichý & Chytrý, 2006; De Caceres & Legendre, 2009). These properties render the Φ index useful for inferring partner specificity (Weißbecker et al., 2019). Within the specificity concept, we distinguish partner *preference* (Φ >0) and *avoidance* (Φ <0) that refer to exclusivity and marginalization of certain partners, respectively. In microbes including fungi, it remains unknown whether partner specificity is related to the preference or avoidance phenomena.

Relative specificity of biotrophic and saprotrophic fungi varies by ecosystem type and the local occurrence of extreme generalists or specialists, such as mycoheterotrophic plants or species of *Alnus* and their reciprocally specific mycorrhizal symbionts (Kennedy et al., 2015; Leonhardt et al., 2019; Põlme et al., 2018). Specificity of fungal‐plant interactions may depend on availability of physiologically and phylogenetically similar partners, co‐occurrence patterns among compatible and incompatible partners, genetic variability of specificity genes in populations of the focal microbial species and the evolutionary pressure to maintain or switch hosts (Bruns et al., 2002). For example, several EcM fungal taxa inferred to be specific turned out to be non‐specific in situations where host plants co‐occur with non‐hosts (i.e., neighbourhood effect; Bogar & Kennedy, 2013) or when EcM host plants become locally extinct (Toftegaard et al., 2010; Perez‐Pasos et al., 2021). In some of these occasions, such ‘untypical’ symbiosis is physiologically efficient enough to allow fungi produce fruiting bodies (Lofgren et al., 2018). While partner specificity is relatively well documented for EcM macrofungi, much less is known about other groups, especially the fungal taxa that produce inconspicuous or no fruiting bodies (Põlme et al., 2018). In temperate and boreal forest ecosystems, 12%–13% of EcM fungi show specificity at the tree genus level (Ishida et al., 2007; van der Linde et al., 2018), but these values reach an exceptional 90% in mixed forests of *Alnus* (Bogar & Kennedy, 2013). Typically, partner‐specific mycorrhizal fungi are considered uncommon or rare in fungal communities (Cullings et al., 2000; Ishida et al., 2007). Similarly, specialist bacteria are rare typically on a larger scale, but they may dominate in specifically suitable microhabitats (Mariadassou et al., 2015). Considering that specialist fungi may be locally dominant in the proximity of their partner tree based on fruiting body production (Buee et al., 2011), it is plausible to assume that the relative proportion of generalists has been overestimated at the stand scale. It also remains poorly known how the rare species, specialists and generalists are related to richness and evenness of fungal communities.

Here we used an extensive stand‐scale molecular dataset of composite soil samples from multiple EcM partner trees to assess the relative effects of partner specificity, partner phylogeny and environmental and spatiotemporal variables on fungal diversity, rarity and distribution of specificity. Using this sampling strategy, our analyses reflect *potential associations* between the partners, because there is no direct evidence for physical connections (van Galen et al., 2023). We tested the following hypotheses: (i) relative specificity differs by plant and fungal taxonomic and functional groups; (ii) partner specific associations increase with stand age (Horton et al., 2005), tree successional status (hypothesised in Taudiere et al., 2015) and relative dominance of tree species; (iii) rare species and specialists contribute most to the local EcM fungal diversity; and (iv) specialist fungi are mostly rare members of the community (Ishida et al., 2007) (see also Table 1). We expected that species of *Alnus* and other early‐successional trees support relatively lower richness and more specific communities of EcM fungi compared with late‐successional trees (Kennedy et al., 2015; Taudiere et al., 2015). We also predicted that the large fungal genera considered as generalist (e.g., *Tomentella, Sebacina, Clavulina*) include a large proportion of specialist species. In certain occasions, we compared other co‐occurring fungal guilds to EcM fungi to put our main findings into context.

**TABLE 1:** Variants of the Φ indicator values and their interpretation.

Φ variants	Explanation	Uses	Hypotheses
Φ_plant_ = Φ (raw)	Indicator value of fungal species for each plant partner	Positive and negative values show preference and avoidance, respectively, for specific plant partners in fungal species	Fungal species of different guilds and ectomycorrhizal (EcM) fungal lineages differ in plant taxon preference and avoidance (e.g., EcM fungi are more specific to *Alnus* but saprotrophs are more specific to conifer genera); average preference and avoidance of fungi differ among certain plant partners (e.g., *Alnus* monocultures host relatively more specific fungi)
|Φ_max_|	The largest absolute value of Φ_plant_ for fungal species across all plant partners	Indicator value showing overall specificity in fungal species	Not applied (prevalence of avoidance was very rare)
Φ_max_	Maximum value of Φ_plant_ for fungal species across all plant partners	Indicator value showing preference in fungal species	Overall partner preference differs among fungal lineages and genera (e.g., species of suilloids and *Alnicola* are generally more specific than other ecm fungi)
Φ_min_	Minimum value of Φ across all hosts	Indicator value showing overall avoidance in fungal species	Partner avoidance differs among fungal guilds (e.g., Glomeromycota tend to avoid certain ecm tree monocultures)
Φ_max,ave_	Average maximum values of Φ_plant_	Average preference for plant partners across all fungal taxa for plots	Partner preference in plots is related to environment (e.g., older tree communities harbour relatively more indicator fungal species)
Φ_min,ave_	Average minimum values of Φ_plant_	Average avoidance for plant partners across all fungal taxa for plots	Average avoidance in plots is related to biotic and abiotic factors (e.g., avoidance is mostly attributable to extremities in soil ph)
Φ^w^_max_	Average abundance‐weighted values of Φ_max,ave_	Average preference for plant partners across all fungal taxa for plots, where averages are weighted by relative abundances	Partner preference in plots is related to environment (e.g., older tree communities harbour relatively greater proportional abundance of indicator fungal species)
Φ_plant,ave_	Average values of Φ_plant_ per plot	Average preference for specific plant taxon across all fungal taxa for plots	Proportion of fungal species preferentially associating with different tree genera differs based on environmental conditions (e.g., the ph optima of particular plant partners)
Φ^W^_plant_	Average abundance‐weighted values of Φ_plant_	Average preference for specific plant taxon across all fungal taxa for plots, where averages are weighted by relative abundances	Abundance‐weighted proportion of fungal species preferentially associating with different tree genera differs based on environmental conditions (e.g., the ph optima of particular plant partners)

## EXPERIMENTAL PROCEDURES

### Sampling

From July 2011 to September 2020, we collected composite soil samples in 1566 plots in the projected area of 80,000 km^2^ in Estonia and Latvia. Altogether 83% of these samples were collected and analysed within the study by Tedersoo et al. (2020). The 242 additional plots sampled in 2020 were particularly focused on covering monocultures of EcM plants, including parks, natural forests and forest plantations. In these monoculture stands, the nearest non‐target EcM trees were not allowed closer than the canopy height to the sampling spots. Similarly, sampling near occasional saplings of non‐focal EcM trees inside plots were avoided by collecting cores at a distance exceeding the height of these plants. This critical distance was determined by following root suckers and tracing roots of various tree species. Roots grow further away from the trunk in tropical ecosystems and stressed habitats (e.g., mining areas), where annual height growth is limited or soils are very shallow (L. Tedersoo, pers. obs.).

Following the Global Soil Mycobiome consortium (GSMc) design (Tedersoo et al., 2020, 2021), we established circular 2500‐m^2^ plots. From each four quadrats of the plots, five trees located at least 8 m apart were randomly selected. From two opposing sides of each 20 trees per plot, 1–1.5 m from the tree trunk, soil cores (5 cm diam. to 5 cm depth) were collected using a sharp knife. The material from all 40 cores per plot was pooled into the same bag, without removing fine roots or small stones (<1 cm diam.). In the last sampling campaign, we additionally used the SoilBON sampling strategy by collecting nine soil cores (5 cm diam. to 10 cm depth) from a 30 × 30 m^2^ plot (Anslan et al., 2021; Guerra et al., 2021). The SoilBON scheme was used when the area was too small for more in‐depth sampling. The composite soil sample was laid on a clean newspaper and air‐dried as soon as possible, within at least 24 h since collection, at <40 °C in a dry room, with heat from the sun, or in an oven.

### Molecular analysis and bioinformatics

The dried samples were kept in zip‐lock plastic bags and homogenized manually by intensively rubbing the bag and its contents by hands for 3 min. DNA was extracted from 2.0 g of soil dust from GSMc samples using the PowerMax Soil DNA Isolation kit (Qiagen, Hilden, Germany). The DNA extracts were further purified using FavorPrep™ Genomic DNA Clean‐Up Kit (Favorgen, Vienna, Austria). The SoilBON samples were subjected to DNA extraction from 0.25 g of soil dust using a KingFisher Flex Purification System (Thermo Fisher Scientific, Waltham, MA, USA) with MagAttract PowerSoil DNA KF kit (Qiagen) following the manufacturer's protocol.

The purified DNA extracts were subjected to amplification of the full‐length ITS region with the universal eukaryotic primers ITS9mun and ITS4ngsUni as described in Tedersoo et al. (2020). Sequencing was performed on PacBio Sequel instrument for samples collected before 2019 (33 1M SMRT cells and on Sequel II instrument for samples collected since 2019 (two 8M SMRT cells). Sample loading was performed by diffusion, and sequencing was performed with a movie time of 600 min and pre‐extension time of 45 min.

Bioinformatics data analysis and taxonomic identification followed Tedersoo et al. (2021) including clustering of quality‐filtered reads at 98% sequence similarity to operational taxonomic units (OTUs) that we refer to as ‘species’. We used FungalTraits (Põlme et al., 2020) to assign species to functional guilds and EcM fungi to phylogenetic lineages (cf. Tedersoo & Smith, 2017). For genera with multiple lifestyles, we used the assignments based on annotations at the level of sequences and species hypotheses (SHs) as given in UNITE 9.1 (Nilsson et al., 2019).

### Environmental variables

Chemical properties of each composite sample were measured from ca. 20 g of dried, homogenized material. Soil pH_KCl_, P, K, Mg and Ca concentration (log‐transformed for statistical analyses to approximate normal distribution) as well as ^15^N and ^13^C abundances were determined following Tedersoo, Bahram, et al. (2012). Phylogenies of woody plant species were adapted from Zanne et al. (2014), and the ultrametric tree (Figure S1) was pruned using the *picante* package of R (Kembel et al., 2010). Using the *adespatial* package of R (Dray et al., 2018), we generated the respective phylogenetic principal coordinates of neighbouring matrices (PCNM) eigenvectors for use as covariates and predictors in variation partitioning analysis.

Based on estimates of basal area of each tree species, we calculated the relative abundance of EcM plants (cf. Soudzilovskaia et al., 2020). Proportion of EcM plants and individual species were log‐ratio‐transformed (+0.01%) to vary from −4 (0.0%) to 0 (50.0%) to 4 (100.0%). The age of vegetation (i.e., age of oldest woody plants, determined based on the database of Estonian Forest Register, https://register.metsad.ee/, or communication with local foresters or land owners), number of woody plant species and EcM plant species sampled were used in both untransformed and square‐root‐transformed formats.

As a proxy for fungal diversity, we used species richness for all fungi, non‐EcM fungi and EcM fungi and the number of EcM fungal lineages. All richness measures were converted to residuals based on regression analyses of logarithm‐transformed richness to logarithm‐transformed sequencing depth to account for differences in sequencing depth (Tedersoo et al., 2022). Read‐based proportions of EcM fungi to all fungi were used untransformed. All used variables are listed in Table S1.

### Partner specificity proxies

In this paper, we define *partner specificity* quantitatively as the degree to which one of the taxa is restricted to its partner taxon, independently of their taxonomic level, nature of the association or vitality. Thus, partner specificity includes associations of saprotrophic organisms with substrates originating from a single partner species, such as wood and litter. Within the partner specificity concept, the term *partner preference* refers to situations where a certain partner taxon is disproportionally commonly represented in these associations. We introduce the term *partner avoidance* to refer to situations where one of the potential partner taxa is marginalized, that is, discriminated against, while the associations with other partners are common.

As a proxy for partner specificity, we calculated Pearson's Φ index (coefficient of association Phi, also known as fidelity) for each fungal species, as implemented in the indicspecies package v.1.7.12 of R (De Caceres & Legendre, 2009; Table 1). The Φ values range from −1 (indicating maximum avoidance) to 1 (indicating maximum preference) (Chytrý et al., 2002). Considering that some hosts have more samples than others, we utilized a version of the index that integrates a correction for unequal group sizes (Tichý & Chytrý, 2006). We assessed the statistical significance of Φ using a permutation test (comprising 9999 unrestricted permutations) as implemented in the permute package (Simpson, 2022). We used the *p*‐value cutoffs at 0.001 to denote strong indicators, and at 0.05 to indicate weak indicators (Figure S2). We interpreted the maximum absolute Φ value (|Φ_max_|) of each fungal species across partner tree taxa as the overall specificity index at specific plant taxonomic levels, that is, species, genus, order and class. The minimum (negative) values, Φ_min_, were interpreted as indicators of relative avoidance. Prior to conducting statistical tests, we performed a power analysis for fungal species associations with each plant taxon to exclude fungal species that fell below the detection threshold in particular tests. In other analyses, we categorized these taxa as ‘rare species’. To test for environmental effects on relative specificity at the ecosystem scale, we calculated the average of the fungal species' Φ_max_ values (Φ_max,ave_), Φ_min_ values (Φ_min,ave_, inferring average avoidance) and fungal Φ_plant_ values of for each plant monoculture (Φ_plant,ave_). In addition, we calculated relative‐abundance‐weighted Φ indices (Φ^W^_max_) by summing the Φ_max_ values of resident species multiplied by their relative abundance (proportion of reads). We mostly refer to Φ_max,ave_ and Φ^W^_max_ indices of partner preference calculated at the plant genus level, where specificity of fungi was the most prominent (Table 1). The schematic representation of the analysis workflow is provided in the Figure S2.

The maximum values of many indicators and host specificity indices are influenced by sample size (Reavie & Juggins, 2011). To address this, we used an alternative metric, the risk ratio (*R*). This metric compares the frequency of fungal species occurrence in a target host to its occurrence in other hosts. To examine the relationship between fungal specificity and prevalence, we utilized the effect size package v.0.8.3 (Ben‐Shachar et al., 2020) to calculate this metric for each host (*R*_host_) and for overall specificity (*R*_max_). The R index can range from 0 to ∞, with values >1 indicating a positive association. An issue arises when a fungal species is found exclusively on one host, resulting in *R*_host_ values of ∞. To mitigate this issue, we substituted the infinite *R*_host_ values of certain fungal species with double the maximum occurrence value observed on another host. This adjustment aligns well with the overall distribution trends of *R*_host_ and *R*_max_. To validate the robustness of this approximation, we tested the multipliers of 1.5 and 5 (instead of 2), but these modifications did not influence the directionality of the relationship or its interpretations. As an alternative metric, we used the Blüthgen d’ index, which is derived from Shannon entropy and quantifies the degree of interaction specialization in bipartite networks, with low sensitivity to variation in sampling intensity (Blüthgen et al., 2006). The Blüthgen d’ index was calculated using the bipartite package v.2.19 (Dormann et al., 2009).

### Statistical analyses

Using generalized mixed‐effects modelling (GLM), we evaluated the effects of plant genus and other floristic (relative abundance of each EcM plant genus, phylogenetic eigenvectors, EcM plant and total woody plant richness, EcM plant proportion, stand age), soil (pH; Ca, Mg, P, K, N and C concentrations) and temporal (year of sampling, month of sampling, linear time) variables on the residuals of fungal biodiversity and proportion of EcM fungi. To mitigate biases related to different sampling schemes, we confined these analyses to 1328 GSMc plots. We also excluded plots with high mould content, low read depth (<50 EcM fungal reads), and those without EcM plants (Table S2). In the models, we incorporated appropriately transformed covariates using both linear and second‐order polynomial functions, and included two‐way interaction terms for categorical predictors such as tree genus, month and year of sampling. We determined the best models based on the backward elimination of variables that contributed <1% to total variance. To test differences in residual richness and proportion of EcM fungi among tree species, genera and lineages, and among tree communities with different EcM plant richness, we performed one‐way ANOVAs, followed by Tukey tests to account for multiple comparisons. We also employed one‐way ANOVAs to assess differences in diversity between native and non‐native EcM plants.

To disentangle the pure and shared effects of environmental and floristic variables on EcM and non‐EcM fungal richness and composition, we performed variation partitioning analyses as implemented in the *vegan* package of R. To separate the effects of plant *species* and phylogeny, we confined the analysis to EcM monoculture plots of the GSMc design and fungal species with a minimum occurrence of five in this data subset (458 plots and 2638 species). Fungal communities were Hellinger‐transformed prior to the analysis. Categorical predictors (plant species, year and month) were converted to dummy variables. The variables were assigned to six partitions: plant species, plant phylogeny (represented by phylogenetic PCNMs), other floristic variables (vegetation age, proportion of EcM basal area), soil variables, temporal variables (year and month) and space (spatial PCNMs). Since the *varpart* function allows testing up to four partitions, we performed the analysis twice, each time using partly overlapping partitions. First, the main variables were tested by using combined floristic variables, soil, time and space. Then, to distinguish the effects of various floristic variables, especially plant phylogeny from species effect, we partitioned the variables to host phylogenetic vectors, host species, other floristic variables, and combined environmental, spatial and temporal predictors. As the variance explained by individual and combined partitions were nearly identical in these two analyses, the resulting values were merged and presented in Venn diagrams.

To identify the factors that govern preference (Φ_max,ave_ and Φ^W^_max_) and avoidance (Φ_min,ave_) in the study plots, we performed GLMs as described above. Using ANOVAs coupled with Tukey tests for unequal sampling, we examined differences in relative specificity among EcM host taxa and fungal genera. Plant taxa and fungal genera with <5 observations were excluded from these tests.

To investigate the relationship between host preference and commonness of fungal species, their Φ_max_ values were regressed against frequency and overall abundance based on read counts. Similar analyses were performed separately for each individual plant genus, using their respective Φ_plant_ values. To understand the effect of plant community dominance on fungal partner preference (Φ_max,ave_), we regressed both the richness‐based and abundance‐based proportions of strong indicators, weak indicators and non‐indicators against a simple dominance measure—the proportion of the most abundant EcM plant species relative to all other EcM plants. To evaluate the potential effect of environmental predictors on fungal partner preference, we regressed the proportions of strong, weak and non‐indicators against soil pH, C/N ratio, N and P concentrations.

To understand whether tree genera differ in their patterns in accumulating more specialists as they become increasingly dominant, we conducted both linear and lowess regressions for Φ_plant,ave_ and Φ^W^_plant_ of each individual plant genus against the relative abundance of this particular taxon. Similar calculations were performed for Φ_max,ave_ and Φ^W^_max_ values in relation to vegetation age in general, and Φ_plant,ave_ and Φ^W^_plant_ for each plant taxonomic group in particular. To further evaluate the dependence of partner preference on soil pH, we performed linear and lowess regressions for Φ_plant,ave_ and Φ^W^_plant_ of each individual plant taxon and soil pH of these specific sites. We also calculated the pH, C/N, N and P range for each fungal species, using their quartiles as a proxy for niche (Helaouët & Beaugrand, 2007), and explored how the range of these environmental conditions correlates with the specificity and commonness of these species. In these plant‐genus‐related calculations, we included all samples where one of the EcM trees accounted for at least 95% of the basal area of all EcM trees.

To assess the direct and indirect effects of predictors on fungal partner preference (Φ_max,ave_ and Φ^W^_max_ values) and richness, and to identify the directionality of relationships between richness and dominance, we used Structural Equation Modelling (SEM) as implemented in the *lavaan* package of R (Rosseel, 2012). For SEM analyses, we included the measure of community evenness (Pielou index) and all significant predictors of various dependent variables, including soil pH and its quadratic form separately, and performed backward elimination of non‐significant variables. The best SEM models were selected based on the corrected Akaike (AICc) information criterion.

## RESULTS

### General findings

This study included 1459 plots with EcM vegetation and 3,872,435 quality‐filtered fungal reads, of which 44.6% were assigned to EcM lifestyle. The dataset revealed 64,787 fungal species, including 18,470 species of EcM fungi (38.9% singletons). On average, each plot harboured 142.0 (±84.9, SD) EcM fungal species (147.1 ± 84.8 in the GSMc design and 66.4 ± 33.1 in the SoilBON design). The monospecific EcM plant data subset includes 474 plots representing 39 EcM plant species and 9993 EcM fungal species.

Of the 64 EcM fungal lineages found, the /tomentella‐thelephora lineage prevailed (5061 species, 27.4% of reads), followed by /cortinarius (2275, 8.2%), /inocybe (2170, 21.7%), /russula‐lactarius (2001, 14.0%) and /sebacina (1191, 5.4%). The most common fungal species were SH4280180.09FU (*Amphinema byssoides*, occupying 605 plots and contributing to 0.97% of all reads), SH1844144.09FU (*Laccaria araneosa*, 514, 0.45%), SH2766094.09FU (*Cenococcum geophilum*, 493, 0.28%), SH3575426.09FU (*Hymenogaster griseus*, 438, 0.51%) and SH3406821.09FU (*Tomentella bryophila*, 425, 0.25%).

### Richness and composition

The GLM models suggested that plant genus, soil pH, EcM tree proportion, EcM tree richness and year of sampling are the key predictors of richness of EcM fungi, EcM lineages, all fungi, non‐EcM fungi and relative abundance of EcM fungi (Table S3). In particular, plant genus explained 17.7% of variation in EcM fungal richness, followed by soil pH (12.9%, unimodal response) and year (9.3%), and positive effects of relative EcM plant proportion (6.8%), soil δ^15^N (i.e., forms of nitrogen, 3.5%) and EcM plant richness (1.5%). The relative abundance of EcM fungi was mostly related to EcM plant proportion (8.8%), soil pH (7.2%, unimodal response) and tree genus (6.8%) but also temporal variables (collectively 26.0%). While the relative abundance of EcM vegetation had a strong positive effect on EcM fungal taxonomic and phylogenetic richness, it had a negative effect on overall fungal richness and non‐EcM fungal richness (Table S3).

**FIGURE 1:**
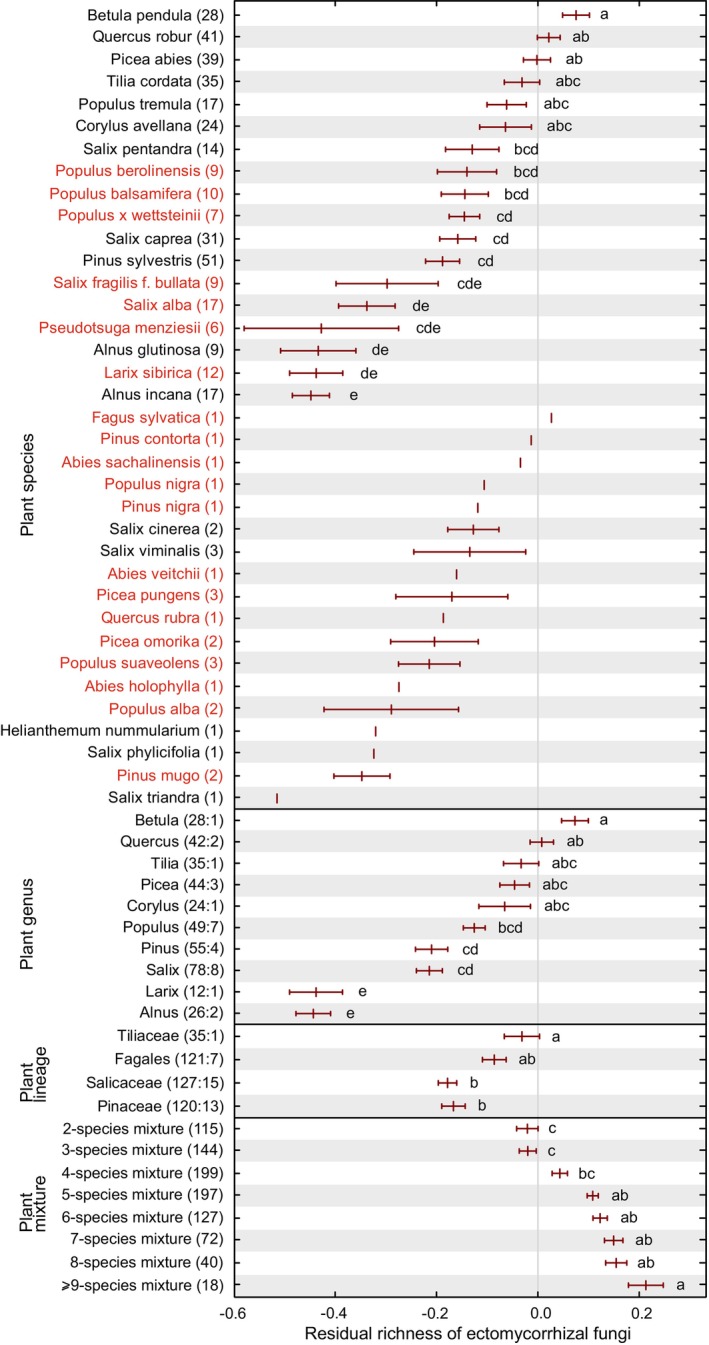
Relative ectomycorrhizal fungal species richness in plant species monocultures compared with mixed stands, as combined into species‐, genus‐ and lineage‐level groups. Only samples collected following the GSMc protocol are included. Different letters indicate statistically significantly different groups. Taxa with no letters had sample size <5 and were hence not tested. Species in red font are considered non‐native; numbers in parentheses indicate sample size, with numbers following the colon depict the number of plant species per genus and lineage.

Ectomycorrhizal plant species, genera and lineages differed substantially in their plot‐based richness estimates of EcM fungi (Figure 1). Relative differences were generally strongest at the level of plant genus compared with species and lineage levels. At the tree species and genus levels, *Betula pendula* had the highest average EcM fungal species richness, whereas *Alnus* spp., *Larix sibirica* and *Pseudotsuga menziesii* had the lowest richness (Figure 1). Similar patterns were evident for the richness of EcM fungal lineages (i.e., phylogenetic diversity; Figure S3). Relative abundance of EcM fungi was also low in these tree species as well as in *Salix alba* and *S. fragilis*. Conversely, total fungal richness and non‐EcM fungal richness were greatest for *Alnus* spp., *Salix pentandra* and *S. alba* but lowest in *Pinus* and *Picea*, which harboured high relative abundance of EcM fungi (Figure S4). At the level of plant lineages, Tiliaceae and Fagales displayed greater EcM fungal species and lineage richness compared with Pinaceae (Figures 1 and S3). Pinaceae had distinctly the lowest overall and non‐EcM fungal richness, especially when compared to Fagales and Salicaceae (Figure S4). Compared with monocultures, mixed EcM plant ecosystems had relatively higher richness of fungi and EcM fungal species and lineages. On average, monoculture stands of the non‐native and native EcM tree species harboured comparable richness of all fungi and EcM fungi (*p* > 0.10; Figure 1).

**FIGURE 2:**
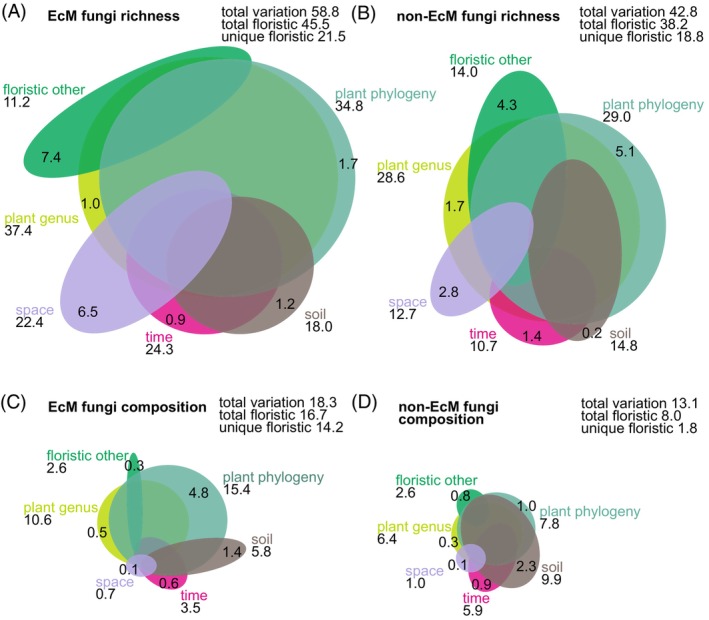
Venn diagrams illustrating variation partitioning of multiple variable groups for richness of ectomycorrhizal fungi (A) and non‐ectomycorrhizal fungi (B) as well as composition of ectomycorrhizal fungi (C) and non‐ectomycorrhizal fungi (D). Numbers inside ellipses and outside ellipses indicate unique and total variance explained (%), respectively, attributed to each partition.

Variation partitioning analyses revealed that floristic variables explained by far the greatest variation in richness and composition in EcM plant monocultures (Figure 2A,B). A moderate proportion of vegetation effect was shared with temporal predictors and spatial predictors as well as temporal and edaphic predictors combined. In general, the plant genus and plant phylogeny effects strongly prevailed, but these could not be efficiently disentangled due to their low unique explained variance. This analysis indicates that plant phylogeny provides little extra information compared with plant genus for EcM fungal richness, that is, richness is weakly affected by plant phylogeny above the genus level. For EcM fungal composition, however, plant phylogeny alone uniquely accounted for 4.8% of the total explained variation, indicating that above‐genus‐level relationships are of great importance in driving EcM fungal community structure. Within the phylogenetic partition, significant phylogenetic PCNM vectors (*p* < 0.001) included PCNM2 (*R* ^2^_adj_ = 0.024; contrasting angiosperms and gymnosperms), PCNM5 (*R* ^2^_adj_ = 0.013; *Pinus* vs. *Picea*), PCNM6 (R^2^_adj_ = 0.011; Fagaceae vs. Salicaceae), PCNM24 (*R* ^2^_adj_ = 0.010; *Alnus* vs. other Betulaceae) and PCNM3 (*R* ^2^_adj_ = 0.010; *Tilia* vs. others). Variation partitioning analyses for non‐EcM fungi revealed several‐fold lower effects of floristic variables but comparable effects of edaphic, spatial and temporal variables on richness and composition (Figure 2C,D).

### Partner specificity in ectomycorrhizal symbiosis

**FIGURE 3:**
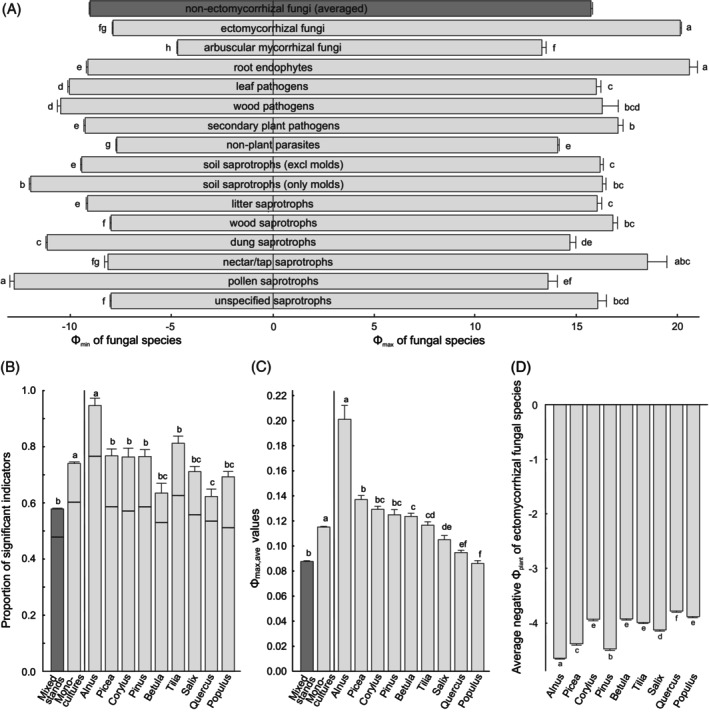
Distribution of partner preference and avoidance in fungi in monospecific stands: (A) preference (right panel) and avoidance (left panel) in functional guilds of fungi; (B) proportion of significant indicators of EcM fungi based on relative number of species (horizontal lines) and relative abundance of reads (bars); (C) average indicator value of all ectomycorrhizal fungal species present in monocultures; (D) partner avoidance in ectomycorrhizal fungi present in monocultures. Mixed forests were excluded from these analyses. Whiskers indicate standard errors, with letters above bars showing statistically significantly different groups.

There were large differences in Φ_max_ and Φ_min_ values among fungal functional guilds at the plant genus level, but no overall differences among mutualistic, antagonistic and saprotrophic lifestyles due to great variation among individual functional guilds (Figure 3A). Avoidance was relatively weaker compared with preference across fungal guilds, except in pollen saprotrophs. On average, root endophytes and EcM fungi had the highest partner preference but relatively low level of partner avoidance. While EcM fungi in *Alnus* stands displayed much greater partner preference than in other monocultures (Figure 3B,C), this situation differed in other functional guilds, where *Pinus* and *Corylus* commonly supported the fungi with strongest partner preference (Figure S5). Species of EcM fungi mostly avoided *Alnus, Pinus* or *Picea* as a partner (Figure 3D), which could be related to the lack of physiological compatibility in *Alnus* and poor compatibility or intolerance of acid soils characteristic of conifers.

Based on |Φ_max_| values, significant partner specificity at any plant taxonomic level was detected for 56.6% of EcM fungal species out of the 4965 species tested (i.e., above detection threshold). High proportions of partner specificity were determined at the level of plant species (29.5%, *n* = 3959), genus (48.4%, *n* = 4693), order (47.3%, *n* = 3422) and class (49.9%, *n* = 2802). The relatively higher proportion of plant genus‐level indicators compared with species‐level indicators suggests that most congeneric plant species (*Alnus* spp. and closely related *Salix* spp.) share their fungal species. There was no significant relationship between phylogenetic distinctness of plant genera (unique branch length) and Φ_max,ave_ and Φ^W^_max_ values (*p* > 0.1). At the plant genus level, there were only four fungal species that displayed slightly stronger avoidance than preference; therefore, we subsequently report mostly preference rather than overall specificity or avoidance.

Mixed stands and monocultures respectively harboured 48.0% and 60.0% of partner‐preferring fungal species on average. These proportions increased to 58.5% and 74.8% when considering the relative abundances (proportion of reads) of significant indicators (Figure 3B). In monocultures of all tree species, partner‐preferring species exceeded 50% in the proportion of species and 60% in the proportion of reads. In particular, significant indicators contributed 77.5% of species and 97.2% of relative abundance in *Alnus* monocultures on average. On the opposite, *Quercus* and *Betula* hosted the largest proportions of generalist EcM fungi (Figure 3B).

**FIGURE 4:**
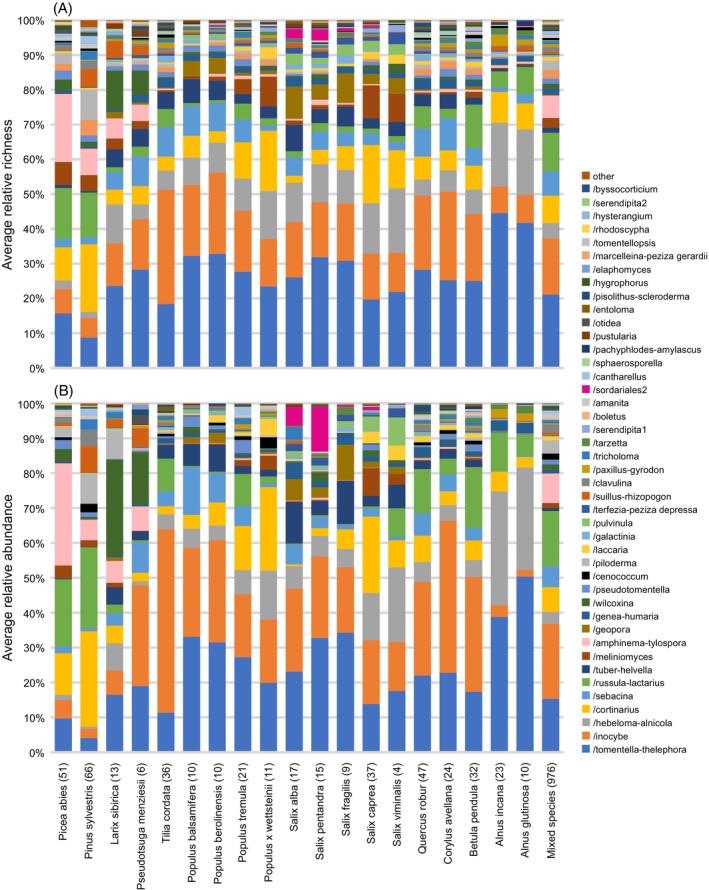
Tree species associations with lineages of ectomycorrhizal fungi based on (A) averaged relative richness and (B) averaged relative abundance. Numbers in parentheses indicate the number of monoculture or mixed species stands. Tree species are ordered by phylogeny and fungal lineages are ordered by the total averaged species richness.

There were large differences in associations between tree species and phylogenetic lineages of EcM fungi. Tree species preferentially associated with distinct groups of EcM fungi. For example, *Picea abies, Pinus sylvestris* and *Tilia cordata* associated particularly commonly with members of the /amphinema‐tylospora, /cortinarius and /inocybe lineages, respectively (Figure 4A,B). A bipartite network graph of individual plant genera and fungal species is shown in Figure S6.

### Distribution of partner preference in EcM fungal taxonomic groups

**FIGURE 5:**
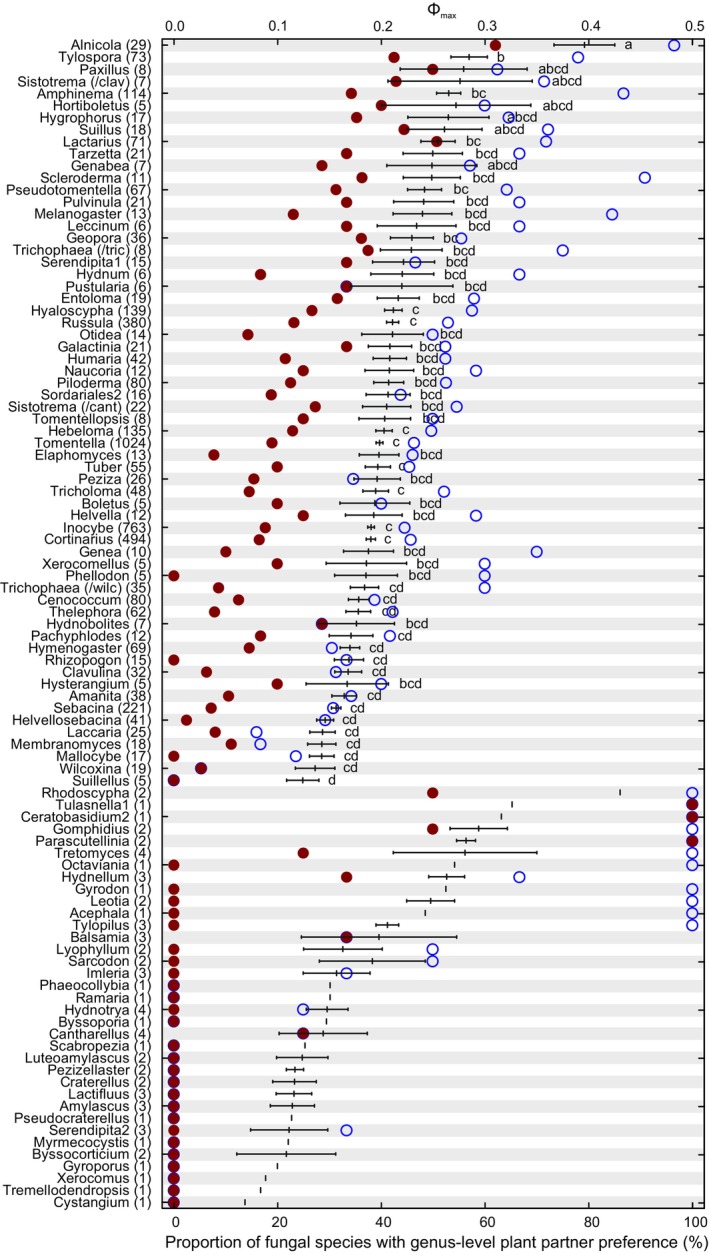
Relative genus‐level host plant preference of ectomycorrhizal fungal genera (n in parentheses). Open and closed circles depict proportion of fungal species with overall significant preference and strong preference, respectively; Lines and error bars indicate the average Φ_max_ index (±SE; y_top_ axis), with letters on the right showing statistically similar difference categories; Genera with *n* < 5 species were excluded from statistical analyses and are displayed for illustrative purpose.

Based on Φ_max_ values and the proportion of strong indicators, EcM fungal genera differed greatly in the proportion of indicators (Figure 5). Of major fungal genera (*n* >4 species), *Alnicola* had distinctly the greatest average indicator values (Φ_max_ = 0.396), followed by *Tylospora* (0.285), *Paxillus* (0.279), *Sistotrema p. parte* (species belonging to the /clavulina lineage; 0.276), *Hortiboletus* (0.272), *Amphinema* (0.265), *Hygrophorus* (0.265) and *Suillus* (0.261). The lowest partner preference was observed for *Suillellus* (0.124), *Wilcoxina* (0.142), *Mallocybe* (0.142), *Membranomyces* (0.143), *Laccaria* (0.143) and genera of the Sebacinaceae family. There was no difference in the relative partner preference in the genera of Basidiomycota and Ascomycota (F_1,95_ = 0.050; *p* = 0.824). Overall, two *Tomentella* species, SH3419813.09FU and SH3384443.09FU (respectively, Φ_plant=Alnus_ = 0.838 and Φ_plant=Alnus_ = 0.815), and *Lactarius cyathuliformis* SH3449865.09FU (Φ_plant=Alnus_ = 0.791) were the strongest indicator taxa (all for *Alnus*).

**FIGURE 6:**
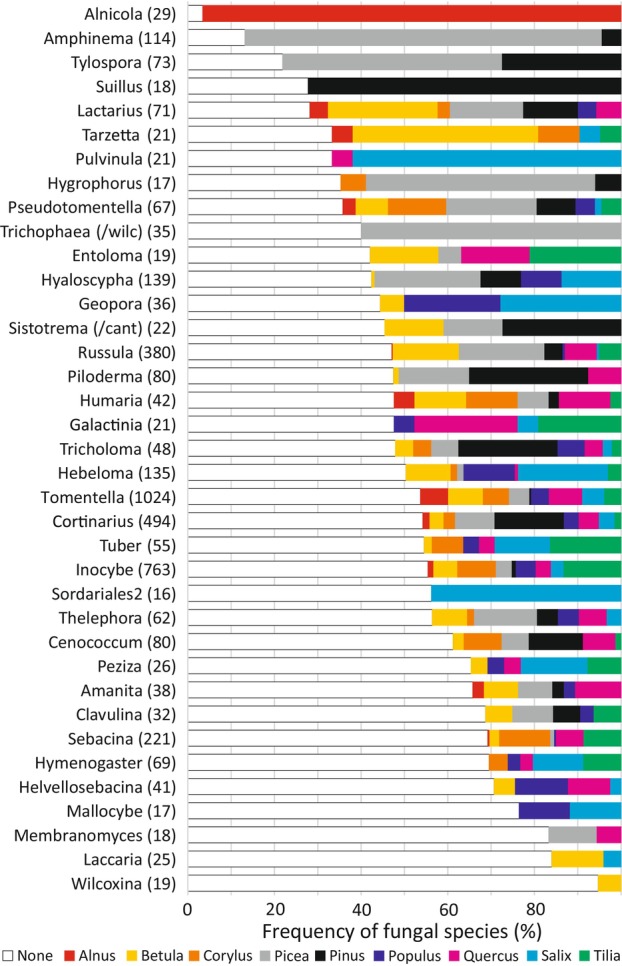
Distribution of partner preference in species belonging to the major genera of ectomycorrhizal fungi in relation to plant genera, as sorted by decreasing specificity. Non‐specific taxa are indicated by open bars (i.e., specificity to none). Size of the bars indicates the proportion of fungal species. Numbers in parentheses indicate the number of fungal species above the frequency threshold. /wilc, /wilcoxina lineage; /cant, /cantharellus lineage.

EcM fungal genera (37 genera with >15 species) also differed in their indicator species distribution among plant partners (Figure 6). For example, *Alnicola* spp. associated with *Alnus*; *Suillus* spp. were indicators of *Pinus*; *Amphinema* spp., *Hygrophorus* spp. and *Trichophaea* spp. (those belonging to the /wilcoxina lineage) were commonly specialists of *Picea*; and species of the unnamed Sordariales genus (/sordariales2 lineage) were preferential associates of *Salix*. Furthermore, *Tylospora* spp. and *Piloderma* spp. were commonly indicators of the Pinaceae genera (*Pinus* or *Picea*), while *Geopora* spp., *Hebeloma* spp. and *Mallocybe* spp. were often indicators of the Salicaceae genera (*Salix* or *Populus*). The largest fungal genera *Tomentella*, *Inocybe*, *Cortinarius*, *Russula* and *Sebacina* had a relatively low proportion of partner specific species, but the significant indicator species within these fungal genera were specialists of multiple tree genera (Figure 6).

**FIGURE 7:**
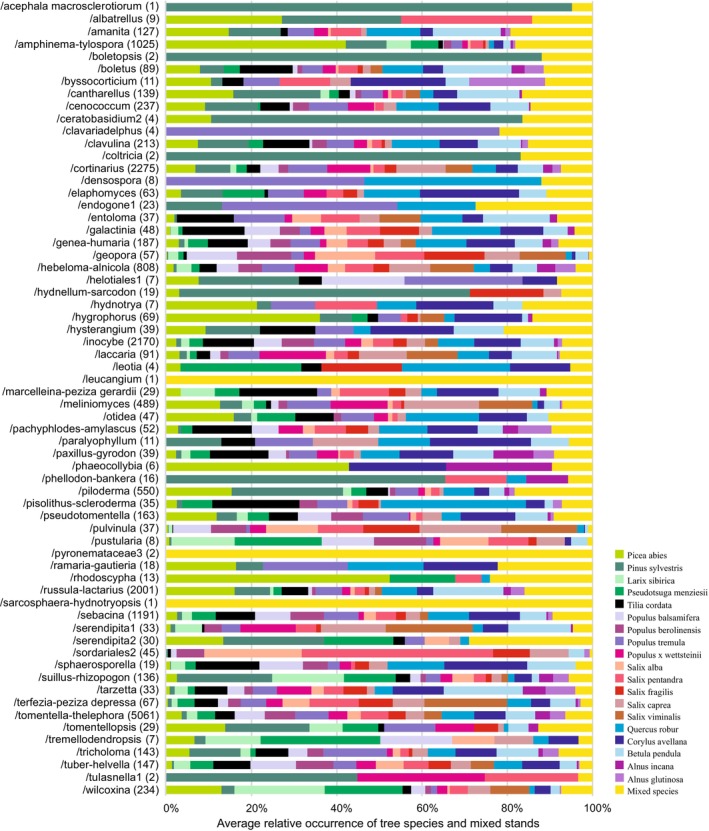
Ectomycorrhizal fungal lineages associating with tree species based on averaged relative occurrence. Numbers in parentheses indicate the number of fungal species used for calculations. Tree species are ordered by phylogeny and fungal lineages are ordered alphabetically.

Besides fungal species, there were strong associations of EcM fungal lineages with certain plant species (Figure 7). For example, the EcM fungal lineages /hygrophorus, /sordariales2 and /pisolithus‐scleroderma were commonly affiliated to *Picea abies, Salix pentandra* and *Quercus robur*, respectively, while only /serendipita2 was relatively more common in mixed stands compared to any particular tree species monoculture.

### Preference‐environment relationships

The proportion and relative abundance of strong fungal indicators increased with increasing tree genus dominance, especially at >95% dominance, whereas weak indicators had no response and non‐indicators declined (Figure S7). Taken separately, relative richness of partner‐preferring fungal taxa increased in all tree genera with their increasing relative abundances, but at different rates (Figure S8). *Alnus, Pinus* and *Picea* responded strongest, while *Quercus, Corylus* and *Betula* displayed the weakest effects. Relative abundances of partner‐preferring taxa closely followed these richness patterns, and were relatively stronger at higher dominance values for all tree genera, particularly *Alnus*, *Pinus, Tilia* and *Corylus* (Figure S8). While the relative richness and abundance of partner‐preferring taxa increased nearly linearly in most tree genera along with increasing abundance, exponential increases were evident in *Alnus*, *Populus* and *Pinus—*from around 30%, 80% and 90% of plant relative abundance, respectively.

The distribution of partner preference in fungal communities was also related to soil pH (Table S4). Relative richness and abundance proportions of strong indicators had U‐shaped relationships with pH, whereas weak indicators had a weak positive relationship and generalists (non‐indicators) had a hump‐shaped relationship (Figure S7). This suggests that strong indicators are commonly specialists of strongly acidic or alkaline soils, which may also reduce their choice among tree partners. However, fungal species‐level analyses showed that there was no difference in niche breadth in terms of soil pH range for specialists and generalists (*p* > 0.05; not shown), suggesting that low pH tolerance is unrelated to preference. At the plot scale, soil pH to Φ_plant,ave_ and Φ^W^_plant_ relationships differed among plant genera, generally peaking at the optimal pH values where the particular monospecific stands occur (Figures S9 and S10). In the genera *Pinus*, *Corylus* and *Quercus*, Φ_plant,ave_ and Φ^W^_plant_ values were greater in relatively acidic soils. In *Tilia* and *Populus*, partner preference was higher in alkaline soils, but in *Alnus* and *Picea*, the Φ^W^_plant_ values peaked at intermediate pH values (Figure S9). In general, the pH‐to‐preference relationship was stronger for weighted than unweighted statistics, suggesting that partner‐related optimal pH favours an increase in relative abundance of specialists.

The Φ_max,ave_ and Φ^W^_max_ values for plots were unrelated to the age of vegetation in monodominant (i.e., >95% dominance) and mixed stands (not shown). However, Φ_plant,ave_ values for *Pinus*, *Quercus* and *Tilia* monocultures increased with age. Similarly, Φ^W^_plant_ values increased in *Pinus* and *Quercus* stands but declined in *Picea* stands (Figure S9). These tree‐specific patterns were unrelated to tree successional status. Furthermore, there were no differences in partner preference and fungal diversity between plantations and naturally regenerating stands.

### Rarity

The total number of rare species of EcM fungi was strongly related to the overall site‐scale EcM fungal richness, which explained 56.4% of variation (Table S3). The proportion of rare species was best explained by soil pH, EcM fungal richness and tree genus. Rare species were relatively more common at intermediate pH level, mixed stands compared with monocultures, and *Pinus* and *Salix* stands compared with *Tilia* stands (Figure S11). The amount and proportion of rare species did not change with stand age or tree successional status.

We found no biologically meaningful relationship between the frequency or total abundance of species and their *R*_max_ or Blüthgen d’ values (Figure S12). Likewise, no significant correlations were evident when testing the fungal commonness and preference relationships in separate analyses of plant partners and the corresponding *R*_plant_ values (not shown). Fungal plant preference was also unrelated to the overall commonness of tree genera (Figure S13). Out of various environmental predictors, only tolerance to soil pH—that is, soil pH range of fungi—increased with commonness of fungal species (Figure S11). Hence, low pH tolerance may be one reason for rarity but not for partner preference.

**FIGURE 8:**
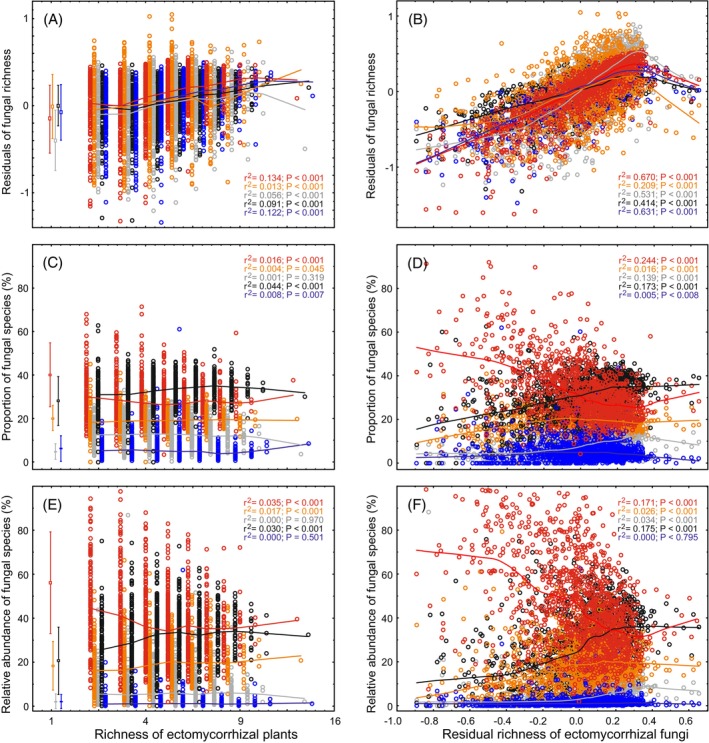
Accumulation of specialist and non‐specialist and rare species of ectomycorrhizal (EcM) fungi with increasing ectomycorrhizal tree richness (left panels) and ectomycorrhizal fungal richness (right panels) based on lowess regression curves: (A, B) ectomycorrhizal fungal richness (residuals accounting for sequencing depth); (C, D) proportion of ectomycorrhizal fungal species; (E, F) proportion of relative abundances of ectomycorrhizal fungi. Red symbols, strong indicators; orange symbols, weak indicators; grey symbols, unassessed species; black symbols, non‐indicators; and blue symbols, the rarest species (not assessed for indicator value). Determination coefficients represent linear fit. For the left panels, the values for single host species are indicated in comparison (box, mean ± SE; whiskers, SD). Monocultures were not included in the regression analyses, because indicator values were calculated based on these, and hence these have inherently higher values for preference and lower values for unknowns.

There were moderately strong relationships between EcM fungal species richness, relative richness and relative abundance of EcM fungal indicators, non‐indicators and the rarest species (frequency <3) on one hand and the overall EcM plant and EcM fungal richness on the other hand (Figure 8). Richness of strong indicators, weak indicators, non‐indicators and the rarest species all increased slightly but significantly with increasing EcM tree species richness and more strongly with increasing overall EcM fungal richness. The greatest increases were evident for strong indicators and the rarest species (Figure 8A,B) in response to increasing EcM tree diversity. Conversely, the relative proportion of strong indicators (in terms of both relative richness and relative abundance) declined with increasing EcM fungal diversity (Figure 8C,D). Only the relative proportion of non‐indicators increased with increasing EcM fungal diversity and relative to indicators and rarest species (Figure 8C–F).

**FIGURE 9:**
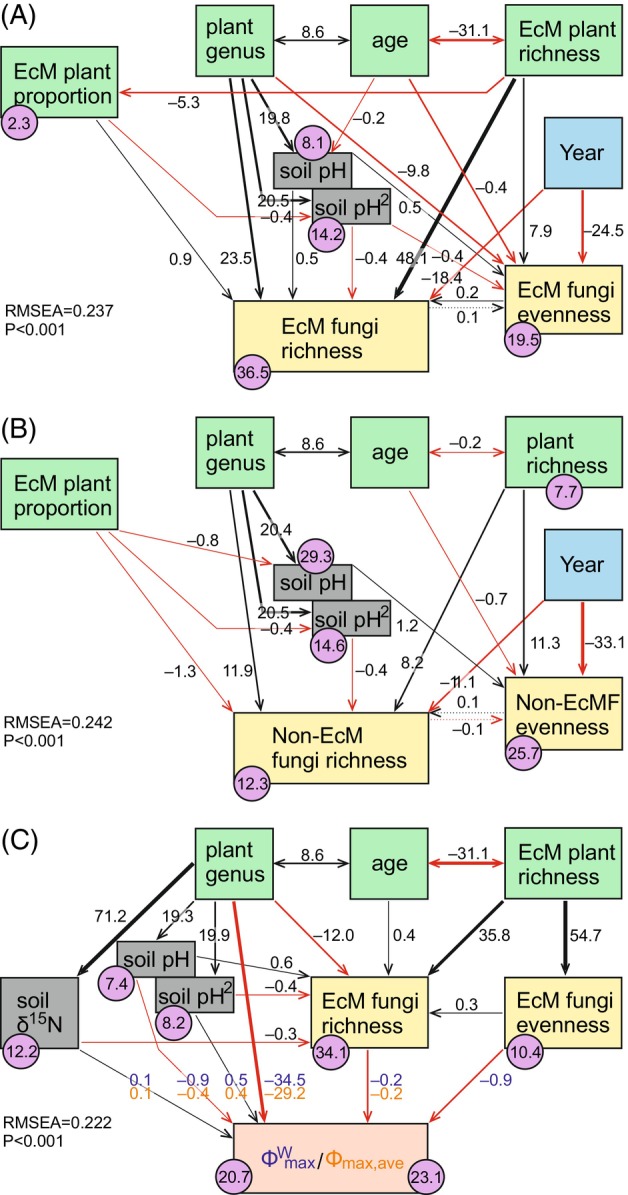
Structural equation models (SEM) explaining richness and evenness of (A) ectomycorrhizal fungi, (B) non‐ectomycorrhizal fungi (non‐EcMF) and (C) partner preference (Φ_max,ave_ and Φ^W^_max_ indices); black and red arrows indicate positive and negative relationships, respectively; double‐headed arrows indicate correlations; numbers on arrows indicate estimated path coefficients; numbers in circles indicate determination coefficients (x100) for variables.

To understand the direct and indirect associations among environmental drivers and vegetation as well as fungal diversity and partner preference, we performed Structural Equation Modelling (SEM). The SEM analysis suggests that EcM plant richness promotes mainly EcM fungal evenness, which in turn contributes to greater stand‐scale EcM fungal richness (Figures 9A and S15). Notably, such an evenness impact loop is negligible for non‐EcM fungi (Figure 9B), and evenness and richness are negatively correlated in most other organisms (Soininen et al., 2012). SEM modelling also revealed that partner preference (Φ_max,ave_ and Φ^W^_max_) is directly related to tree genus and soil pH (Figure 9C), but an additional small but significant proportion of the tree genus effect is indirect through soil pH. EcM fungal richness and evenness had a weak negative effect on preference, indicating that diversity and preference are negatively related.

## DISCUSSION

### Diversity and partner specificity

Tree species and especially tree genera differ greatly in the richness of all fungi, EcM fungi and lineages of EcM fungi on a stand scale, which confirm previous implications of fungal richness differences at the individual tree scale (Ishida et al., 2007; Kennedy et al., 2003; Wang et al., 2019). Across monospecific stands, plant species have the strongest effects on EcM fungal richness, whereas accounting for phylogenetic relationships among plants adds little extra information to the species effect. On the contrary, plant phylogeny alone accounts for 15% of explained variation in community composition, demonstrating for the first time that, indeed, pure phylogenetic relationships among species are important in structuring EcM fungal communities. Previous studies were unable to separate plant *species* and *phylogeny* effects due to a low number of replicates per plant species and no explicit testing (Ishida et al., 2007; Miyamoto et al., 2022; Põlme et al., 2013; Tedersoo et al., 2013; Wang et al., 2019; Wu et al., 2018). PCNMs reveal that the phylogeny effect is most apparent at the EcM plant genus and order levels. Plant phylogeny and taxonomy effects were much lower in non‐EcM fungi, probably due to the lack of obligate associations with tree roots.

Our results indicate that fungal species in different functional guilds tend to specialize on rather than avoid specific plant partners, but to a greatly different extent. Among fungal guilds, EcM fungi and putative root endophytes display the strongest partner preference patterns. This confirms previous findings of remarkable partner specificity in EcM fungi in other ecosystems (Kennedy et al., 2015; Põlme et al., 2018) and a previous study on root endophytes (Macia‐Vicente et al., 2008). However, the comparison of EcM fungi to other guilds may be somewhat affected by the fact that AM herbs, shrubs and trees are nearly always present in EcM plant monocultures and these plants certainly serve as hosts for non‐EcM fungi, especially for AM fungi.

More than half of the EcM fungal species were specific at a certain plant taxonomic level across the study area. At the plot scale, partner‐preferring EcM fungal species contributed 52% of species and 64% of relative abundance across mixed and monospecific stands on average. These values exceed the estimated 12%–13% share of specialist species based on root tip surveys in mixed (Ishida et al., 2007) and monoculture (van der Linde et al., 2018) stands in temperate forests. Therefore, our results challenge the old view that partner generalist fungi dominate in EcM fungal communities (e.g., Cullings et al., 2000; Ishida et al., 2007; Kennedy et al., 2003). We anticipate that differences among studies also depend on the investigated plant species (Kennedy et al., 2012; Walker et al., 2014), such as inclusion of the ultra‐specific *Alnus* (Kennedy et al., 2015) and highly promiscuous Arbutoideae (Molina & Trappe, 1982).

Both plant and fungal taxa display strong differences in partner preference, in line with the first hypothesis. Preference at plant genus, order and class levels is comparable, but it greatly exceeds species‐level preference, confirming previous implications (Ishida et al., 2007; Wu et al., 2018). Plant genera also differ from each other in terms of infrageneric differences: species of *Alnus* (sister species *A. incana* and *A. glutinosa*) share a large proportion of indicators, while species of *Salix* and *Populus* display more distinct association patterns, roughly following their classification to subgenera.

Ectomycorrhizal fungal genera differ greatly in partner preference of their individual species and preference for specific plant genera, most of which are novel observations. The shared preference of species within the fungal genera *Amphinema* (preference for *Picea*), *Tylospora* (*Picea* and Pinaceae), *Tarzetta* (*Betula*), *Geopora* (Salicaceae) and *Piloderma* (Pinaceae) towards one of the tree genera or families is unexpected, because members of these fungal genera usually turn out to be multi‐partner species in community analyses of EcM root tips (Ishida et al., 2007; Tedersoo et al., 2008).

We report the lowest partner preference in the EcM fungal genera *Wilcoxina*, *Laccaria* and *Membranomyces*. *Wilcoxina* and *Laccaria* species are so‐called pioneer fungi that commonly associate with multiple plant species in early successional habitats, including forest nurseries (Mikola, 1965; Newton, 1992). Our analyses also reveal that the genera *Hyaloscypha* and *Cenococcum*, with two and one described EcM fungal species, respectively, comprise tens of cryptic species (previously described for *Cenococcum*; Obase et al., 2017), many of which display preference towards particular partner genera, providing evidence for niche differentiation.

Although the high tree genus‐ and subgenus‐level preference of species of *Alnicola*, *Suillus* and *Rhizopogon* is well established (Molina et al., 1992, 1997; Rochet et al., 2011), our analyses reveal lower than expected indicator values for the two suilloid genera. *Suillus* spp. may form EcM associations with other untypical partner trees (Perez‐Pazos et al., 2021) perhaps more commonly than expected. *Suillus* and *Rhizopogon* species are exceptionally good dispersers by spores (Ashkannejhad & Horton, 2006). *Rhizopogon* spp. form a persistent spore bank in soil (Glassman et al., 2015), with a high likelihood of detection distant from their actual habitats. This suggests that we may detect dormant propagules originating from nearby stands, and that our soil‐based estimates of specificity may be systematically influenced by dispersal capacity and longevity of spores.

For many tree genera, preferential recruitment of specialists benefits from nearly absolute focal plant species dominance. These results corroborate our second hypothesis and previous observations that monospecific tree plantations harbour greater relative abundance of specialists compared with mixed plantations (Weissbecker et al., 2019). The stronger response of abundance‐weighted (Φ^W^_max_) than unweighted (Φ_max,ave_) indicators shows that the *relative abundance* of specialists increases more rapidly than their *richness*, suggesting that specialists may gain a competitive advantage under high dominance of their preferred plant partner species, perhaps due to greater physiological compatibility (Bruns et al., 2002) or adjustment to specific soil conditions as reflected by the soil pH preferences. However, our analyses show that specialists and generalists display a similar abiotic niche breadth for pH and soil properties in general. Furthermore, at least *Alnus*‐related specialists tolerate a broad range of edaphic and climatic conditions (Põlme et al., 2013; Roy et al., 2013).

High abundance of specialists may benefit the partner trees by keeping other plant species poorly connected to the mycorrhizal networks for reducing carbon drain by mycoheterotrophs and retaining competitive advantage relative to late‐successional trees (Kennedy et al., 2015). Although some early‐successional trees (*Alnus* spp.) show relatively higher fungal preference, specificity patterns found in other trees do not support the dominance maintenance hypothesis related to the successional status. Although commonly assumed, there is no experimental evidence that partner‐specific associations provide relatively stronger benefits to their plant partners (Bogar et al., 2019; Hoeksema et al., 2018; Kennedy et al., 2020; Parlade & Alvarez, 1993; Perry et al., 1989; but see Gorissen & Kuyper, 2000). As host specificity is more common in parasites (Barrett et al., 2009, but see Figure 3A), it is equally plausible that EcM fungal specialists lean towards parasitism, potentially receiving more carbon relative to providing mineral nutrients. Unfortunately, we still have very limited information on the relative functioning of specialists and generalists, and how they trade in biological markets.

Soil pH affects the relative richness and abundance of partner‐preferring and generalist EcM fungi, with specialists prevailing at pH extremes (<3.5 and >5.5–6.5). This pattern may be partly driven by the genus *Pinus* that grows at highly acidic soils, modifies the soil accordingly through its needle litter, and hosts relatively specific fungi especially at the lower pH extremes (Figure S7). Since monocultures of other trees are not naturally found in highly acidic soils, it remains to be examined whether the ultra‐low pH specialist fungi are truly pine‐specific or capable of colonizing roots of other subordinate EcM plants in these forests. It also remains to be experimentally studied whether the pine‐associated microbiota further magnify soil acidification to sustain their prevalence (Thorley et al., 2015). Given the similar pH niche breadth of specialists and generalists on one hand and differential pH‐to‐preference relationships among fungi colonizing different plant partners on the other hand, the co‐development of partner specificity and edaphic niche specialization remains an open question. However, in other organisms, specialization along different niche axes is unrelated (Carscadden et al., 2020; Chaloner et al., 2020).

There is context‐dependent support to the hypothesis that relatively older forests favour specialists (Horton et al., 2005), because accumulation of indicators with forest age depends on tree species in monocultures (this study) and mixed forests (Boeraeve et al., 2018). Arrival of specialists for relatively uncommon tree species, such as *Q. robur* and *T. cordata*, may simply take time from disconnected habitats. To test this possibility, we performed Mantel tests for fungal communities of each plant genus separately. Unlike in the entire EcM fungal community, congeneric monoculture stands generally displayed weak dispersal limitation, but its slope was unrelated to tree relative abundance or other features (Figure S14). For EcM fungi in *Tilia* and *Populus* monocultures that had relatively high dispersal limitation, we found that strong indicators exhibited stronger dispersal limitation compared with weak indicators and non‐indicators. The spatial analysis shows that EcM fungi generally exhibit low dispersal limitation in the forest‐dominated landscape (for open landscape, see Peay et al., 2010), but this is relatively higher for strong specialists. In particular, EcM fungal specialists of rare tree taxa may have greater difficulties establishing in suitable habitats because of paucity of suitable partner trees, as suggested for specialist wood decomposers (Norros et al., 2012).

We found that diverse tree communities accumulate species of EcM fungi that are classified as specialists, generalists or rarest. In disagreement with the third hypothesis, generalists benefit most from higher EcM plant richness and contribute relatively more to highly diverse EcM fungal communities. Hence, the accumulation of specialists and rarest species is only a weak contributor to the diversity‐begets‐diversity paradigm (cf. Whittaker, 1975), challenging such previous suggestions for EcM symbiosis (Ishida et al., 2007; Tedersoo et al., 2016). Our results agree with studies on bacteria, where generalists maintain diversity of the community by greater persistence and speciation rates (Sriswasdi et al., 2017).

### Rarity

Contrary to our fourth hypothesis, there is no evidence for relatively higher partner preference among rare species, suggesting that rarity is unrelated to partner specificity in EcM symbiosis. Using a similar composite sample metabarcoding approach, van Galen et al. (2023) indicated that Nothofagaceae tree species uniquely explain 4% variation in the composition of rare EcM fungal species but 0% in common and dominant species, compared with 30%–35% variation uniquely explained by abiotic variables. Our results are in partial agreement with a bird‐parasite association study, where commonness of different *Haemoproteus* species had a weak positive relationship with specificity, but commonness of *Leucocytozoon* species did not (Ellis et al., 2020).

Rare species display a lower pH range compared with common species, suggesting that abiotic niche breadth rather than partner specificity (i.e., biotic niche) is one of the drivers of rarity in EcM fungi. Narrow niche in terms of habitat specificity is inferred as one of the mechanisms of rarity in plants (Boulangeat et al., 2012) and other microorganisms (Godon et al., 2005). It is also possible that rare fungal species are more vulnerable to specific microbial antagonists or consumers based on evidence in plants (Klironomos, 2002) and bacteria (Kurm et al., 2019). Rare plant species are also commonly inferred as weak competitors (Griggs, 1940), but rare species of bacteria and EcM fungi are not necessarily inferior competitors (Kennedy et al., 2020; Kurm et al., 2019).

### Methodological considerations

While most plant‐fungal specificity studies have been performed on physically interacting, co‐occurring partners (reviewed in Põlme et al., 2018), our study tests partner specificity issues in monospecific stands (see also van der Linde et al., 2018; van Galen et al., 2023). Consequently, here we address *potential* fungal‐plant interaction patterns rather than realized specificity. Additionally, our study does not account for the neighbourhood effect, such as root contacts and influence of litter, which may promote increase in partner range and partner shifts (Jairus et al., 2011; Bogar & Kennedy, 2013; Perez‐Pazos et al., 2021). Although this approach excludes sampling of non‐target EcM plants, fungal spores distributed from nearby stands may blur the inferred specificity patterns, as discussed above for the suilloid fungi. However, our approach enables us to cover thousands of fungal species and nearly all native and non‐native plant species, which do not naturally co‐occur in any single plant community. Furthermore, spatially more distant forest stands are statistically independent, and represent the tree and fungal *species* better compared with multiple samples collected from one or two forest sites (Blüthgen et al., 2006).

Our analyses may also suffer from the challenges related to delimitation of biological species in fungi. Lumping of species with too conservative clustering threshold, as suspected for *Hebeloma* (Eberhardt et al., 2016), *Leccinum* (den Bakker et al., 2004), *Cortinarius* (Garnica et al., 2016) and *Alnicola* (Rochet et al., 2011), may blur specificity patterns. Conversely, oversplitting of taxa, as we suspect for Atheliaceae that comprise a few tens of described EcM fungal species, may magnify the effects of particular biological species relative to other species. Many of the rare species certainly correspond to undetected sequencing artefacts or uncommon haplotypes of dominant species (Runnel et al., 2022).

The quantitative assessment of partner preference and avoidance using the Φ index is a step forward compared with the qualitative tests for overall patterns in each species individually as based on non‐parametric tests. This allowed us estimate the relative level of specificity in all plant and fungal species with at least three observations, and revealed that in most fungal groups, preference patterns are stronger than avoidance. Further development of indicator indices for specificity would benefit from inclusion of a phylogenetic relatedness component and accounting for absolute or relative abundances in individual samples. The current abundance‐aware indicator algorithms penalize against uneven abundances rather than account for small or high absolute/relative abundances (De Caceres & Legendre, 2009).

### Synthesis and conclusions

We demonstrate that partner specificity, especially partner preference by both plants and fungi, is more common in EcM fungal symbiosis than previously expected and not restricted to conspicuous taxa with comprehensive fruiting body observations. In particular, more than one half of the fungal species display significant preference for certain plant taxa, but avoidance and exclusive specificity are less common. Species of EcM fungi show the greatest preference towards their plant partners at the genus level, although there is strong evidence for preference at the levels of subgenus, lineage and class, usually following phylogenetic relationships among plants. Conserved association patterns of congeneric EcM fungal species towards specific host plants suggest plant‐fungal coevolution in multiple taxonomic groups of fungi. We find that specialist EcM fungi accumulate with increasing proportion of their intimate partner plants and at soil pH extremes, but inconsistently so in relation to vegetation age and successional status. In EcM fungal species, specificity is unrelated to rarity or environmental niche breadth, but rare species exhibit relatively narrow pH niche. Generalists contribute relatively more than specialists and rare species to high stand‐level biodiversity of EcM fungi in mixed forests.

Our results indicate that monocultures of certain broadleaved trees, such as birch, support diverse fungal communities, but monospecific plantations of conifers offer the least benefit in supporting fungal diversity and biodiversity of other organisms (Hua et al., 2022) and should be therefore avoided in forestry practices. In addition to mixed stands, old monospecific stands are also valuable due to accumulation of more specialists that are less common in mixed stands. It is essential to conserve stands dominated by rare tree species (e.g., *Tilia cordata* and *Salix pentandra*), because these harbour multiple EcM fungi not found in other habitats, although the overall fungal diversity may not be particularly high.

## AUTHOR CONTRIBUTIONS

**Leho Tedersoo:** Conceptualization; investigation; funding acquisition; writing – original draft; methodology; validation; visualization; writing – review and editing; formal analysis; project administration; data curation; supervision; resources. **Rein Drenkhan:** Funding acquisition; formal analysis; resources; supervision. **Kessy Abarenkov:** Methodology; software; data curation; resources. **Sten Anslan:** Investigation; methodology; formal analysis. **Mohammad Bahram:** Investigation; formal analysis. **Kriss Bitenieks:** Investigation. **Franz Buegger:** Resources; formal analysis. **Daniyal Gohar:** Formal analysis. **Niloufar Hagh‐Doust:** Investigation; formal analysis. **Darta Klavina:** Investigation. **Kristaps Makovskis:** Investigation. **Austra Zusevica:** Investigation. **Karin Pritsch:** Resources; formal analysis. **Allar Padari:** Formal analysis. **Sergei Põlme:** Investigation. **Saleh Rahimlou:** Investigation; formal analysis. **Dainis Rungis:** Investigation; funding acquisition. **Vladimir Mikryukov:** Investigation; writing – review and editing; visualization; software; formal analysis; resources.

## CONFLICT OF INTEREST STATEMENT

The authors declared no conflicts of interest.

## Supporting information


**FIGURE S1.** Ultrametric phylogram of ectomycorrhizal plant species in Estonia and Latvia used for calculating phylogenetic distances among plant species.

**FIGURE S2.** Schematic representation of the workflow for estimation of the indicator values (Φ). Various Φ indices are accentuated with arrows; for interpretation of different variants of Φ, see Table 1. (A) Significance thresholds of Φ; (B) An example of Φ value distribution among four different plant hosts. Each point represents a unique fungal species (OTU); four example OTUs observed on all plant hosts are highlighted with colour; (C) Distribution of Φ values across all fungal OTUs for four individual plant hosts.

**FIGURE S3.** Relative ectomycorrhizal fungal lineage richness in plant species monocultures compared with mixed stands, as combined into species‐, genus‐, lineage‐ and phylum‐level groups. Only samples collected following the GSMc protocol are included. Different letters indicate statistically significantly different groups. Taxa with no letters had sample size.

**FIGURE S4.** Relative richness of (A) non‐ectomycorrhizal fungi and (B) all fungi and relative abundance of ectomycorrhizal fungi (C) in plant species monocultures belonging to different genera. Bars and whiskers indicate mean and standard error, respectively. Different letters indicate statistically significantly different groups. Numbers following plant genus names indicate sample size.

**FIGURE S5.** Average plant partner preference of fungal species belonging to different functional groups of fungi as based on their Φ_plant_ values. Whiskers depict standard errors and letters indicate statistically significant difference groups. Secondary plant pathogens include taxa for which plant pathogen is a non‐primary lifestyle; nonplant pathogens include parasites of fungi, animals and protists.

**FIGURE S6.** Bipartite Network indicating associations between plant genera (grey circles) and species of ectomycorrhizal fungi (coloured circles). For plants, circle size indicates the weighted degree (number of links), which is related to both sample size and average number of fungal species. The bipartite network graph was prepared by using incidence data as implemented in the *igraph* package of R (Csardi & Nepusz, 2006; International Journal of Complex Systems, 1695, 1–9) and visualized in Gephi software (Bastian et al., 2009; Proceedings of the International AAAI Conference on Weblogs and Social Media, 3, 361–362).

**FIGURE S7.** Distribution of indicator and non‐indicator ectomycorrhizal fungal species in habitats with differing dominance (relative basal area of the dominant EcM tree species, with AM tree proportions discounted; top panels) and pH (bottom panels) on the basis of relative species richness and abundance of EcM fungi in different indicator categories. Circles represent the proportion of strong indicators in samples. Note the sharp increase in strong indicator richness and abundance at very high (>95%) dominance and at pH extremes.

**FIGURE S8.** Relationship between relative abundance of partner tree genera and weighted (Φ^W^_plant_; blue circles and curves) and unweighted (Φ_plant,ave_; orange circles and red curves) plot‐based Φ‐values for the respective partner tree genera. Samples with the lack of focal hosts were excluded from analyses). Pearson correlations for linear fit are indicated; all *p*‐values are <0.001.

**FIGURE S9.** Relative effects of soil pH (top panels) and vegetation age (bottom panels) on the average partner preference in monocultures using unweighted (Φ_plant,ave_, left panels) and weighted (Φ^w^_plant_, right panels) values of focal host tree genera (red, Alnus; orange, Betula; yellow, Corylus; grey, Picea; black, Pinus; dark blue, Populus; magenta, Quercus; light blue, Salix; green, Tilia). Only significant linear and second‐order polynomial regressions are indicated.

**FIGURE S10.** Distribution of tree species in the soil pH gradient. Only monocultures (here, at least 95% relative abundance of all EcM plants) are indicated, with grey bars indicating focal tree genera and blue bars depicting all tree genera. The scale corresponds to the focal tree genus.

**FIGURE S11.** Rarity in ectomycorrhizal symbiosis: (A) The effect of tree genus on the proportion of rare species; (B) The effect of soil pH on the proportion of rare species; (C) Relationship between fungal species frequency and soil pH range based on the quartiles (i.e., niche breadth). For the quartile‐based calculation, species with occurrence in <4 sites were excluded. Note the logarithmic scale in the x‐axis.

**FIGURE S12.** Relationship between partner specificity and commonness of ectomycorrhizal fungi as based on (A) and (B), the *R*_max_ risk ratio metric, and (C) and (D), the Blüthgen d’ interaction specialization metric, using species frequency (A) and (C) and total abundance (number of reads; (B) and (D) as proxies for commonness. Note the logarithmic scale in the abundance plots. We suspect that the Blüthgen d’ metric is inherently positively related to frequency of observations for uncommon species (present in 1–10 samples).

**FIGURE S13.** Relative abundance of trees in Estonia: (A) relative volume of tree genera; (B) relative area covered by monocultures (defined here as at least 95% of volume belonging to a single tree species; corresponds to 17.8% of the entire state forest area); (C) the lack of significant relationship between volume‐based tree abundance and average Φ_max,ave_ values for each host. The tree abundance data are taken from the Estonian Forestry Register (https://register.metsad.ee).

**FIGURE S14.** Distance‐decay relationships among monospecific plots and all plots taken together (large panel) based on Bray–Curtis dissimilarity measure. In the extra graphs for Populus and Tilia, distance‐decay relationships were calculated separately for strong indicators of the target genus (red and magenta symbols), weak indicators of the target genus (orange symbols) and non‐indicators (black symbols). Lowess curves depict non‐linearity of these relationships. Statistics indicate linear Pearson correlations. Note that the relatively low distance decay across all plots is related to the lack of accounting for tree species and environment.

**FIGURE S15.** Relationships of (A) ectomycorrhizal plant richness and (B) ectomycorrhizal fungal richness with Pielou evenness index, magenta symbols) and dominance (proportional abundance of the most common species, black symbols). Note the logarithmic scale in (A).

**TABLE S1.** Plot‐based metadata used for statistical analyses.

**TABLE S2.** Samples used in the richness analysis. Additional metadata and OTU distribution are given in the datasets stored in the PlutoF repository.

**TABLE S3.** Best generalized mixed models for diversity parameters of fungi.

**TABLE S4.** Best models for Φ_max,ave_ and Φ^W^ values.

## Data Availability

DNA sequences recovered in this study were deposited in NCBI Sequence Read Archive (SRA) under accession numbers SRR23544682–SRR23545466, SRR23549188–SRR23550136, and SRR23550137–SRR23550424 (BioProject PRJNA936817, BioSample IDs SAMN33370525–SAMN33371103, SAMN33373544–SAMN33374090, SAMN33375111–SAMN33375312). Representative sequences of OTUs are available through the UNITE database. The bioinformatics pipeline and scripts used for the analysis are available on GitHub: https://github.com/Mycology‐Microbiology‐Center/GSMc, https://github.com/Mycology‐Microbiology‐Center/EcM‐partner‐specificity. The following datasets are available in the PlutoF repository: full‐size sample‐by‐OTU matrix with metadata (corresponds to Tables S1 and S4), matrices used for variation partitioning and SEM (https://doi.org/10.15156/BIO/2483937).
