# Supplementary material for: The influence of tree genus, phylogeny, and richness on the specificity, rarity, and diversity of ectomycorrhizal fungi
Source: Environ Microbiol Rep. 2024 Apr 4;16(2):e13253. doi: 10.1111/1758-2229.13253 (PMC10994715; doi:10.1111/1758-2229.13253)
Supplement: Supplementary file 1 — FIGURE S1. Ultrametric phylogram of ectomycorrhizal plant species in Estonia and Latvia used for calculating phylogenetic distances among plant species. [file EMI4-16-e13253-s016.pdf]

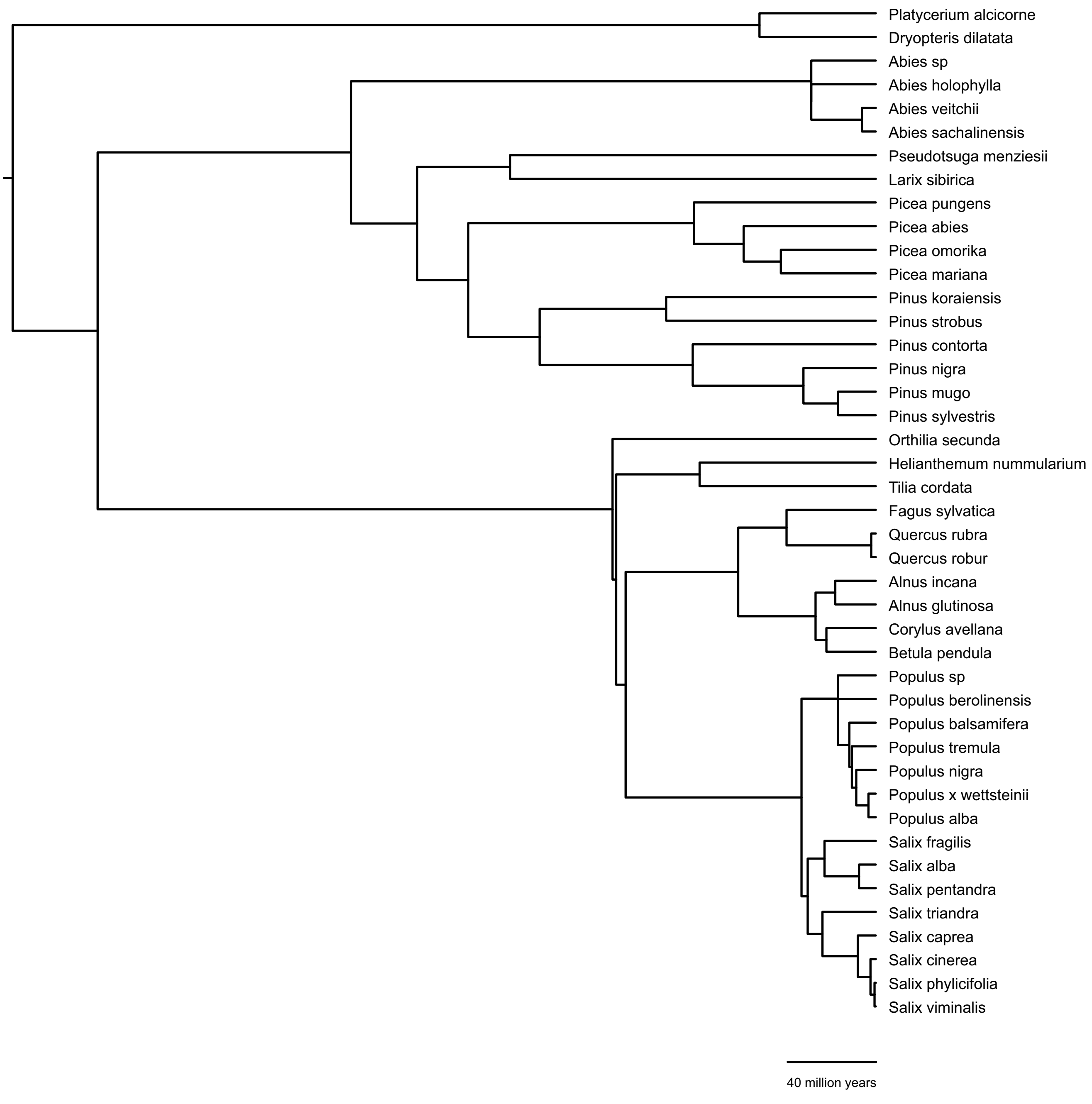

FIGURE S1 Ultrametric phylogram of ectomycorrhizal plant species in Estonia and Latvia used for calculating phylogenetic distances among plant species.
