# Supplementary material for: The influence of tree genus, phylogeny, and richness on the specificity, rarity, and diversity of ectomycorrhizal fungi
Source: Environ Microbiol Rep. 2024 Apr 4;16(2):e13253. doi: 10.1111/1758-2229.13253 (PMC10994715; doi:10.1111/1758-2229.13253)
Supplement: Supplementary file 2 — FIGURE S2. Schematic representation of the workflow for estimation of the indicator values (Φ). Various Φ indices are accentuated with arrows; for interpretation of different variants of Φ, see Table 1. (A) Significance thresholds of Φ; (B) An example of Φ value distribution among four different plant hosts. Each point represents a unique fungal species (OTU); four example OTUs observed on all plant hosts are highlighted with colour; (C) Distribution of Φ values across all fungal OTUs for four individual plant hosts. [file EMI4-16-e13253-s007.pdf]

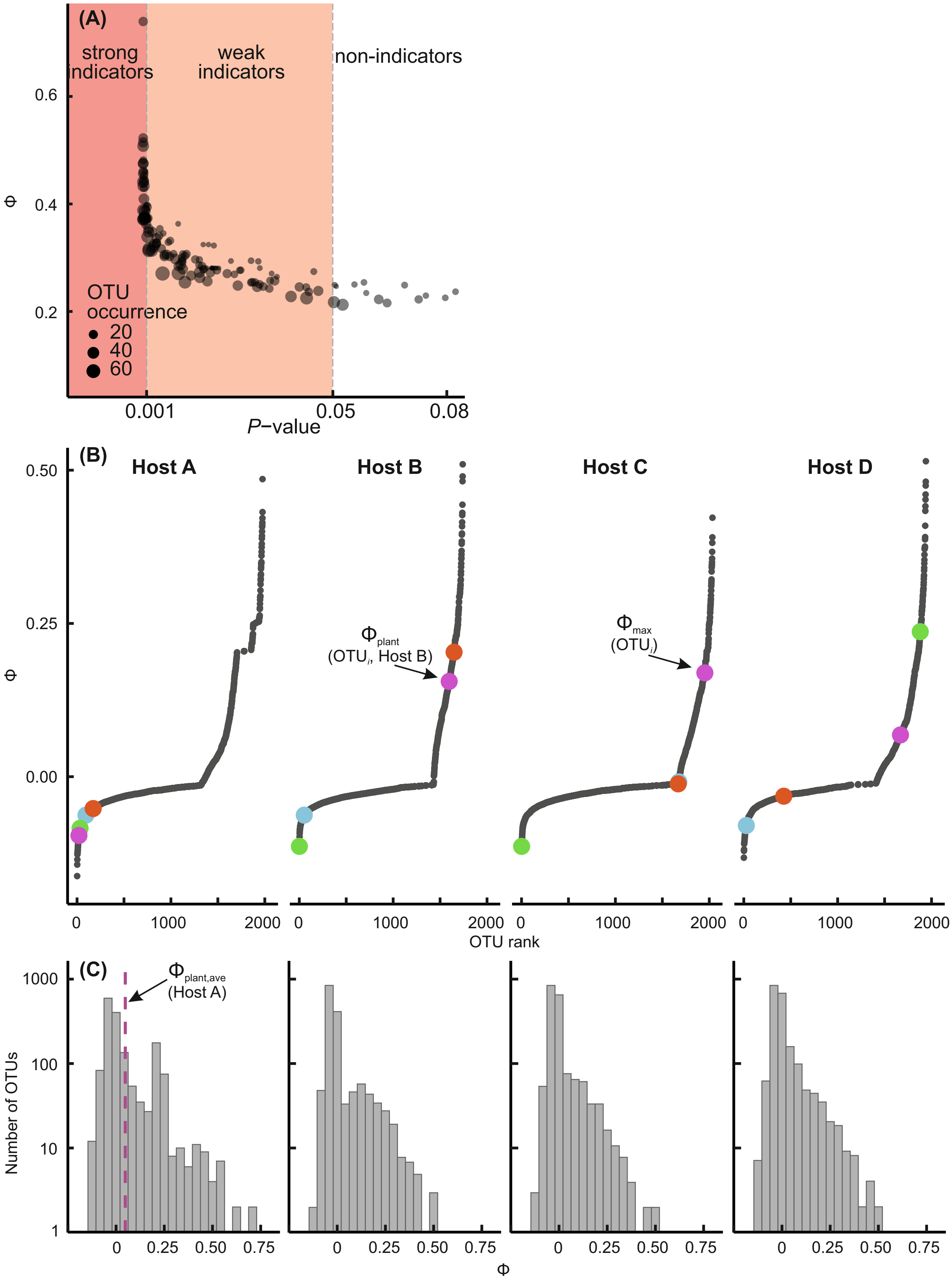

FIGURE S2 Schematic representation of the workflow for estimation of the indicator values (Φ). Various Φ indices are accentuated with arrows; for interpretation of different variants of Φ, see Table 1. (A) Significance thresholds of Φ; (B) An example of Φ value distribution among four different plant hosts. Each point represents a unique fungal species (OTU); four example OTUs observed on all plant hosts are highlighted with color; (C) Distribution of Φ values across all fungal OTUs for four individual plant hosts.
