# Supplementary material for: The influence of tree genus, phylogeny, and richness on the specificity, rarity, and diversity of ectomycorrhizal fungi
Source: Environ Microbiol Rep. 2024 Apr 4;16(2):e13253. doi: 10.1111/1758-2229.13253 (PMC10994715; doi:10.1111/1758-2229.13253)
Supplement: Supplementary file 3 — FIGURE S3. Relative ectomycorrhizal fungal lineage richness in plant species monocultures compared with mixed stands, as combined into species‐, genus‐, lineage‐ and phylum‐level groups. Only samples collected following the GSMc protocol are included. Different letters indicate statistically significantly different groups. Taxa with no letters had sample size. [file EMI4-16-e13253-s015.pdf]

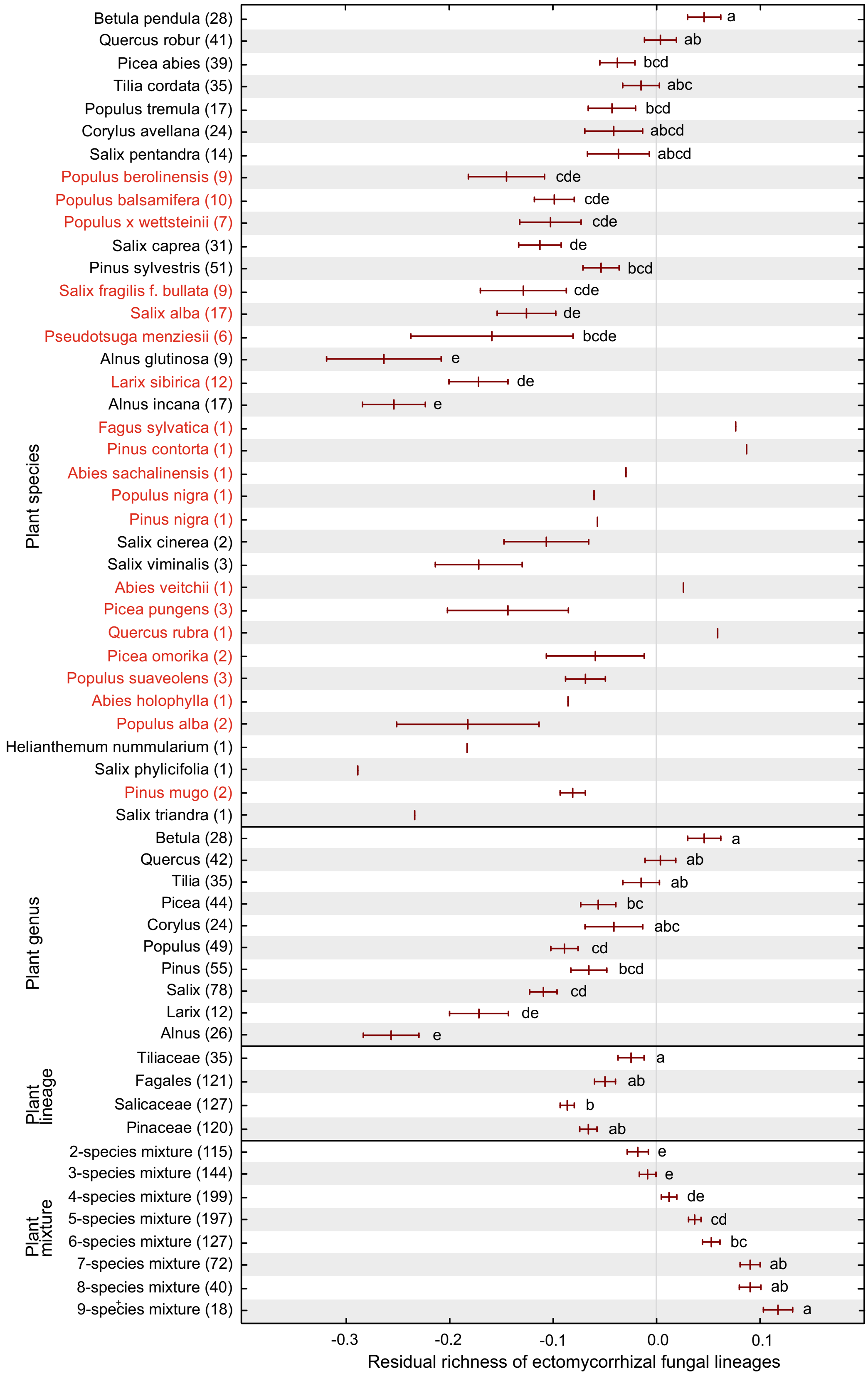

FIGURE S3 Relative ectomycorrhizal fungal lineage richness in plant species monocultures compared with mixed stands, as combined into species-, genus-, lineage- and phylum-level groups. Only samples collected following the GSMc protocol are included. Different letters indicate statistically significantly different groups. Taxa with no letters had sample size <5 and were hence not tested. Species in red font are considered non-native; numbers following plant names indicate sample size.
