# Supplementary material for: The influence of tree genus, phylogeny, and richness on the specificity, rarity, and diversity of ectomycorrhizal fungi
Source: Environ Microbiol Rep. 2024 Apr 4;16(2):e13253. doi: 10.1111/1758-2229.13253 (PMC10994715; doi:10.1111/1758-2229.13253)
Supplement: Supplementary file 4 — FIGURE S4. Relative richness of (A) non‐ectomycorrhizal fungi and (B) all fungi and relative abundance of ectomycorrhizal fungi (C) in plant species monocultures belonging to different genera. Bars and whiskers indicate mean and standard error, respectively. Different letters indicate statistically significantly different groups. Numbers following plant genus names indicate sample size. [file EMI4-16-e13253-s011.pdf]

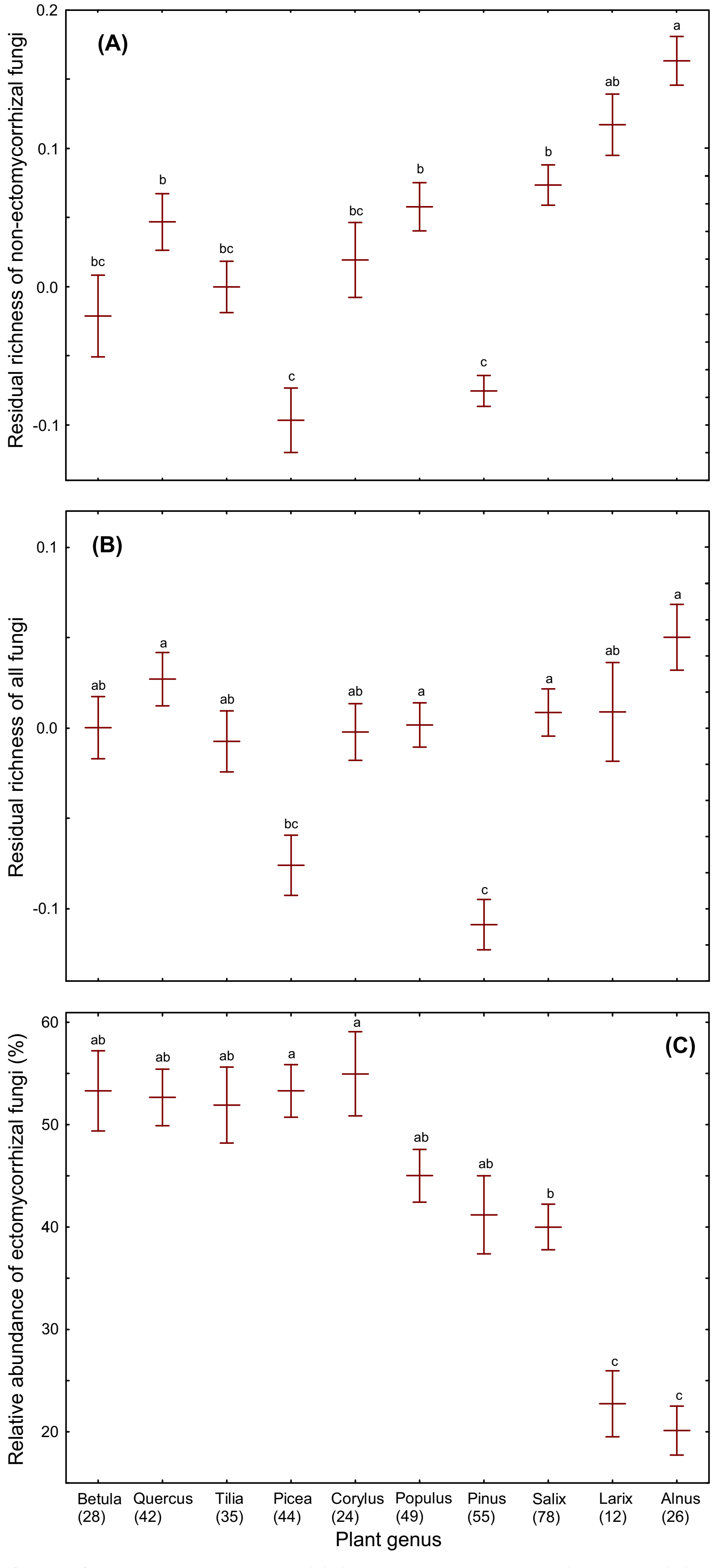

FIGURE S4 Relative richness of (A) non-ectomycorrhizal fungi and (B) all fungi and relative abundance of ectomycorrhizal fungi (C) in plant species monocultures belonging to different genera. Bars and whiskers indicate mean and standard error, respectively. Different letters indicate statistically significantly different groups. Numbers following plant genus names indicate sample size.
