# Supplementary material for: The influence of tree genus, phylogeny, and richness on the specificity, rarity, and diversity of ectomycorrhizal fungi
Source: Environ Microbiol Rep. 2024 Apr 4;16(2):e13253. doi: 10.1111/1758-2229.13253 (PMC10994715; doi:10.1111/1758-2229.13253)
Supplement: Supplementary file 5 — FIGURE S5. Average plant partner preference of fungal species belonging to different functional groups of fungi as based on their Φplant values. Whiskers depict standard errors and letters indicate statistically significant difference groups. Secondary plant pathogens include taxa for which plant pathogen is a non‐primary lifestyle; nonplant pathogens include parasites of fungi, animals and protists. [file EMI4-16-e13253-s003.pdf]

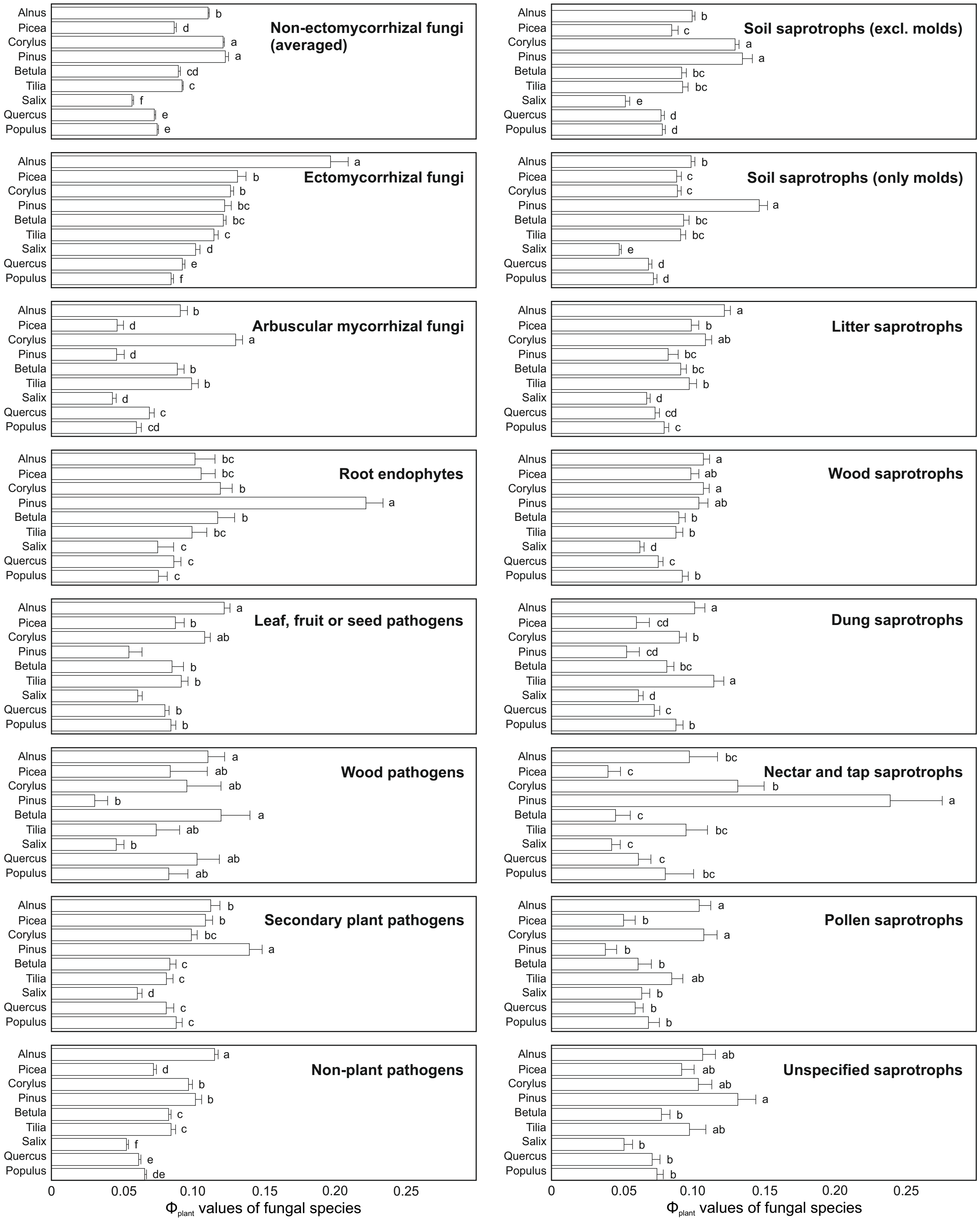

FIGURE S5 Average plant partner preference of fungal species belonging to different functional groups of fungi as based on their Φ_{plant} values. Whiskers depict standard errors and letters indicate statistically significant difference groups. Secondary plant pathogens include taxa for which plant pathogen is a non-primary lifestyle; non-plant pathogens include parasites of fungi, animals and protists.
