# Supplementary material for: The influence of tree genus, phylogeny, and richness on the specificity, rarity, and diversity of ectomycorrhizal fungi
Source: Environ Microbiol Rep. 2024 Apr 4;16(2):e13253. doi: 10.1111/1758-2229.13253 (PMC10994715; doi:10.1111/1758-2229.13253)
Supplement: Supplementary file 6 — FIGURE S6. Bipartite Network indicating associations between plant genera (grey circles) and species of ectomycorrhizal fungi (coloured circles). For plants, circle size indicates the weighted degree (number of links), which is related to both sample size and average number of fungal species. The bipartite network graph was prepared by using incidence data as implemented in the igraph package of R (Csardi & Nepusz, 2006; International Journal of Complex Systems, 1695, 1–9) and visualized in Gephi software (Bastian et al., 2009; Proceedings of the International AAAI Conference on Weblogs and Social Media, 3, 361–362). [file EMI4-16-e13253-s010.pdf]

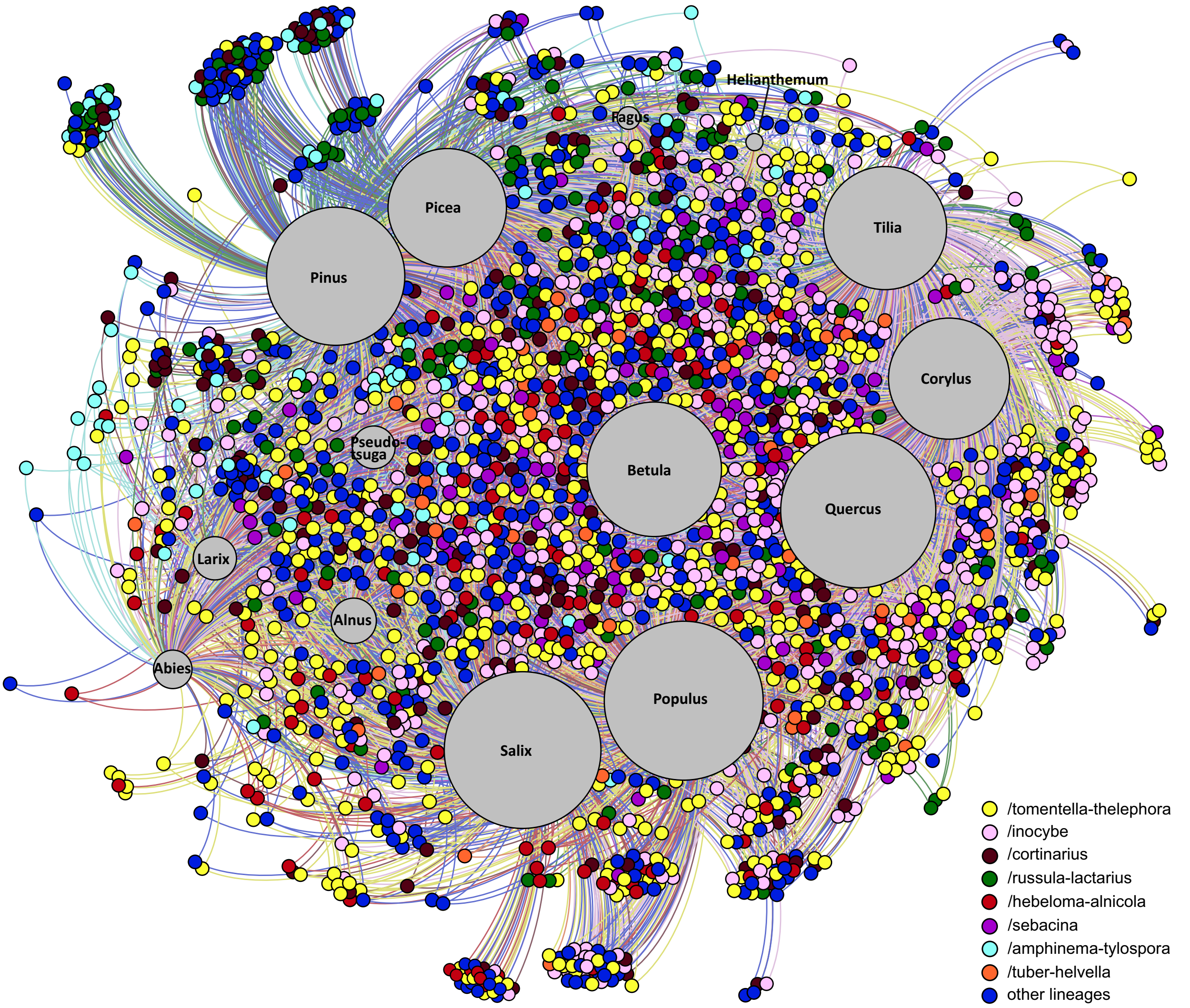

FIGURE S6 Bipartite Network indicating associations between plant genera (grey circles) and species of ecto-mycorrhizal fungi (coloured circles). For plants, circle size indicates the weighted degree (number of links), which is related to both sample size and average number of fungal species. The bipartite network graph was prepared by using incidence data as implemented in the *igraph* package of R (Csardi & Nepusz, 2006; *International Journal of Complex Systems*, 1695, 1-9) and visualised in Gephi software (Bastian et al., 2009; *Proceedings of the International AAAI Conference on Weblogs and Social Media*, 3, 361-362).
