# Supplementary material for: The influence of tree genus, phylogeny, and richness on the specificity, rarity, and diversity of ectomycorrhizal fungi
Source: Environ Microbiol Rep. 2024 Apr 4;16(2):e13253. doi: 10.1111/1758-2229.13253 (PMC10994715; doi:10.1111/1758-2229.13253)
Supplement: Supplementary file 7 — FIGURE S7. Distribution of indicator and non‐indicator ectomycorrhizal fungal species in habitats with differing dominance (relative basal area of the dominant EcM tree species, with AM tree proportions discounted; top panels) and pH (bottom panels) on the basis of relative species richness and abundance of EcM fungi in different indicator categories. Circles represent the proportion of strong indicators in samples. Note the sharp increase in strong indicator richness and abundance at very high (>95%) dominance and at pH extremes. [file EMI4-16-e13253-s012.pdf]

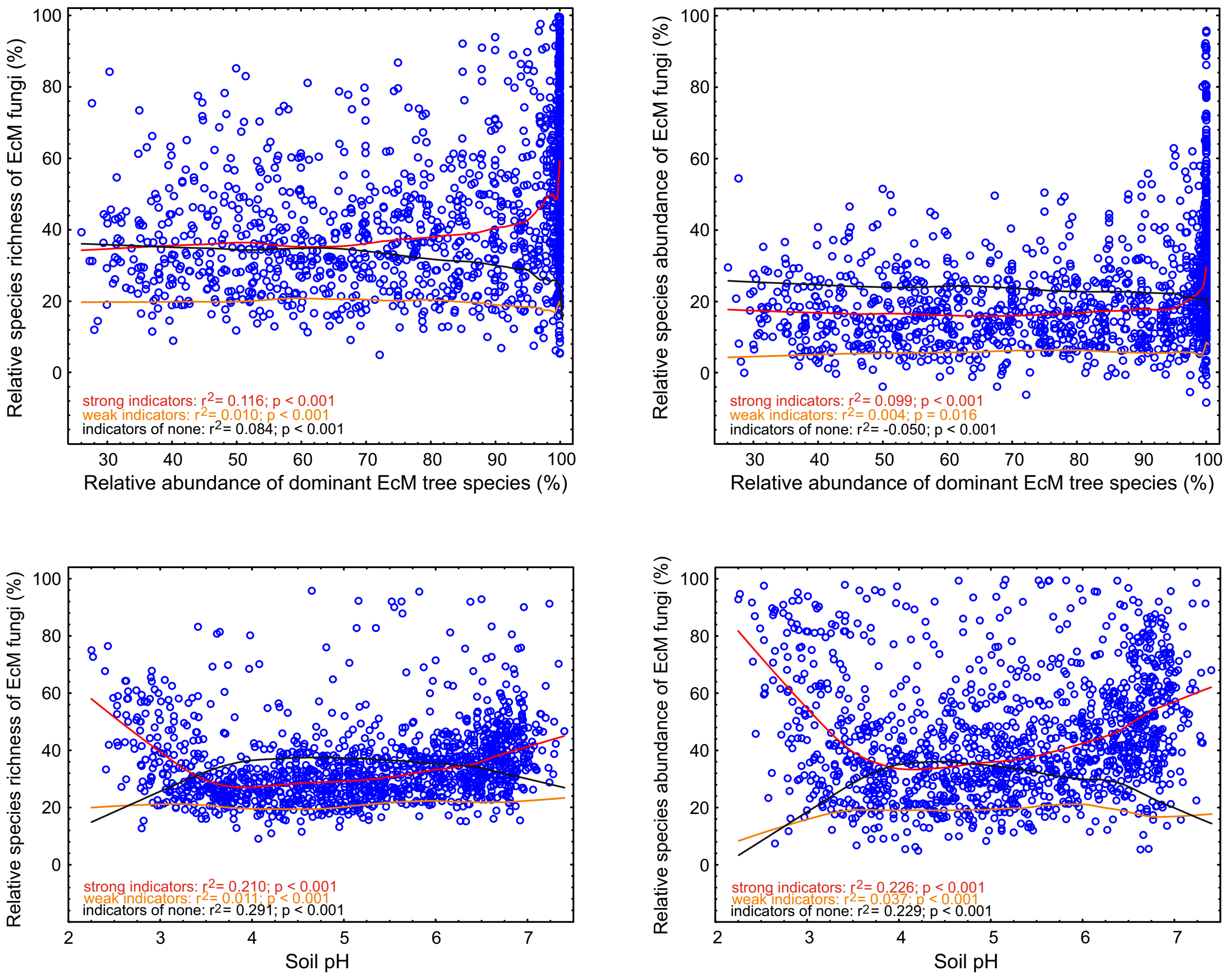

FIGURE S7 Distribution of indicator and non-indicator ectomycorrhizal fungal species in habitats with differing dominance (relative basal area of the dominant EcM tree species, with AM tree proportions discounted; top panels) and pH (bottom panels) on the basis of relative species richness and abundance of EcM fungi in different indicator categories. Circles represent the proportion of strong indicators in samples. Note the sharp increase in strong indicator richness and abundance at very high (>95%) dominance and at pH extremes.
