# Supplementary material for: The influence of tree genus, phylogeny, and richness on the specificity, rarity, and diversity of ectomycorrhizal fungi
Source: Environ Microbiol Rep. 2024 Apr 4;16(2):e13253. doi: 10.1111/1758-2229.13253 (PMC10994715; doi:10.1111/1758-2229.13253)
Supplement: Supplementary file 8 — FIGURE S8. Relationship between relative abundance of partner tree genera and weighted (ΦW plant; blue circles and curves) and unweighted (Φplant,ave; orange circles and red curves) plot‐based Φ‐values for the respective partner tree genera. Samples with the lack of focal hosts were excluded from analyses). Pearson correlations for linear fit are indicated; all p‐values are <0.001. [file EMI4-16-e13253-s004.pdf]

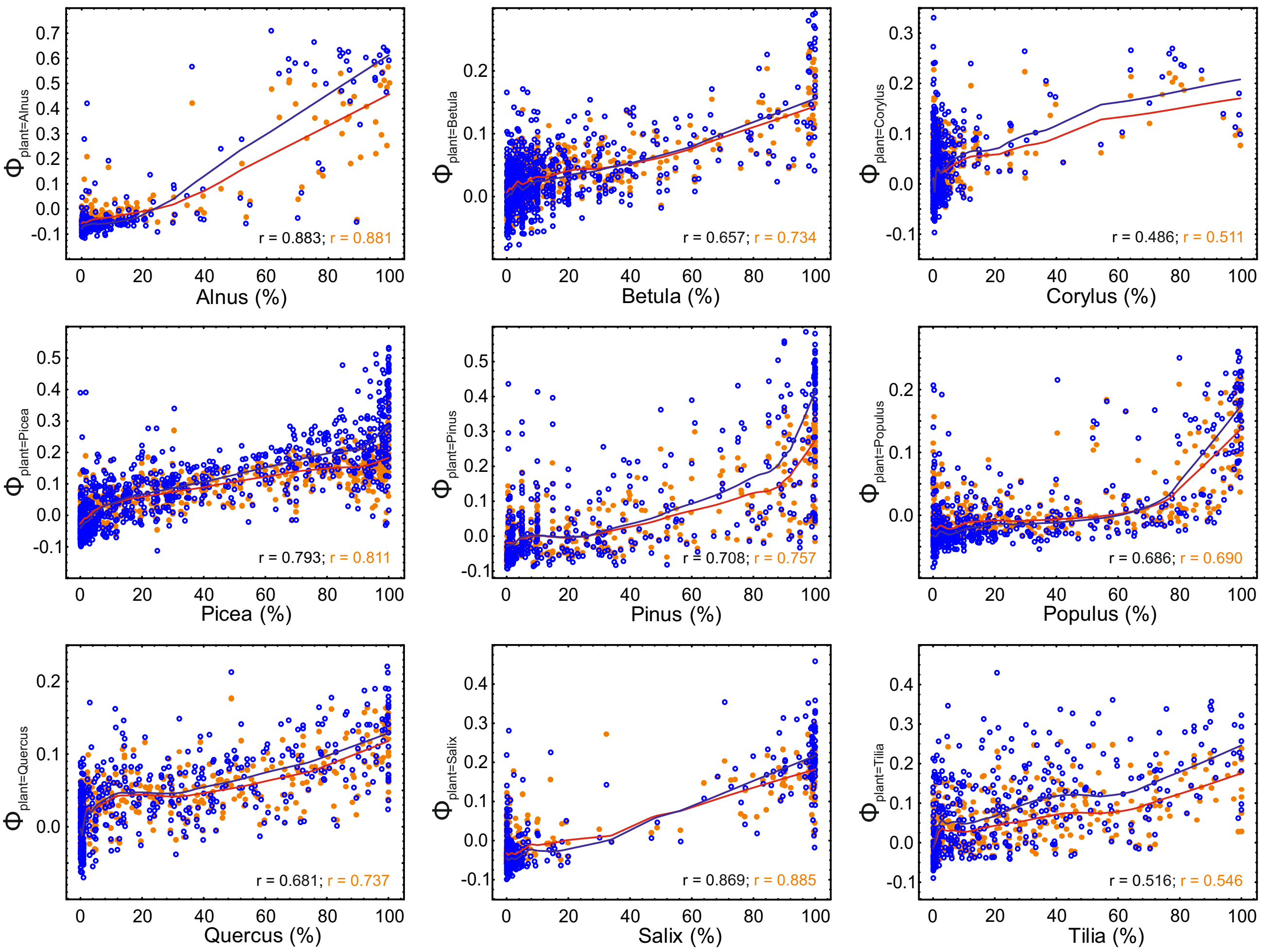

FIGURE S8 Relationship between relative abundance of partner tree genera and weighted (Φ^{W}_{plant}; blue circles and curves) and unweighted ($\Phi_{plant,ave}$; orange circles and red curves) plot-based Φ-values for the respective partner tree genera. Samples with the lack of focal hosts were excluded from analyses). Pearson correlations for linear fit are indicated; all *P*-values are <0.001.
