# Supplementary material for: The influence of tree genus, phylogeny, and richness on the specificity, rarity, and diversity of ectomycorrhizal fungi
Source: Environ Microbiol Rep. 2024 Apr 4;16(2):e13253. doi: 10.1111/1758-2229.13253 (PMC10994715; doi:10.1111/1758-2229.13253)
Supplement: Supplementary file 9 — FIGURE S9. Relative effects of soil pH (top panels) and vegetation age (bottom panels) on the average partner preference in monocultures using unweighted (Φplant,ave, left panels) and weighted (Φw plant, right panels) values of focal host tree genera (red, Alnus; orange, Betula; yellow, Corylus; grey, Picea; black, Pinus; dark blue, Populus; magenta, Quercus; light blue, Salix; green, Tilia). Only significant linear and second‐order polynomial regressions are indicated. [file EMI4-16-e13253-s017.pdf]

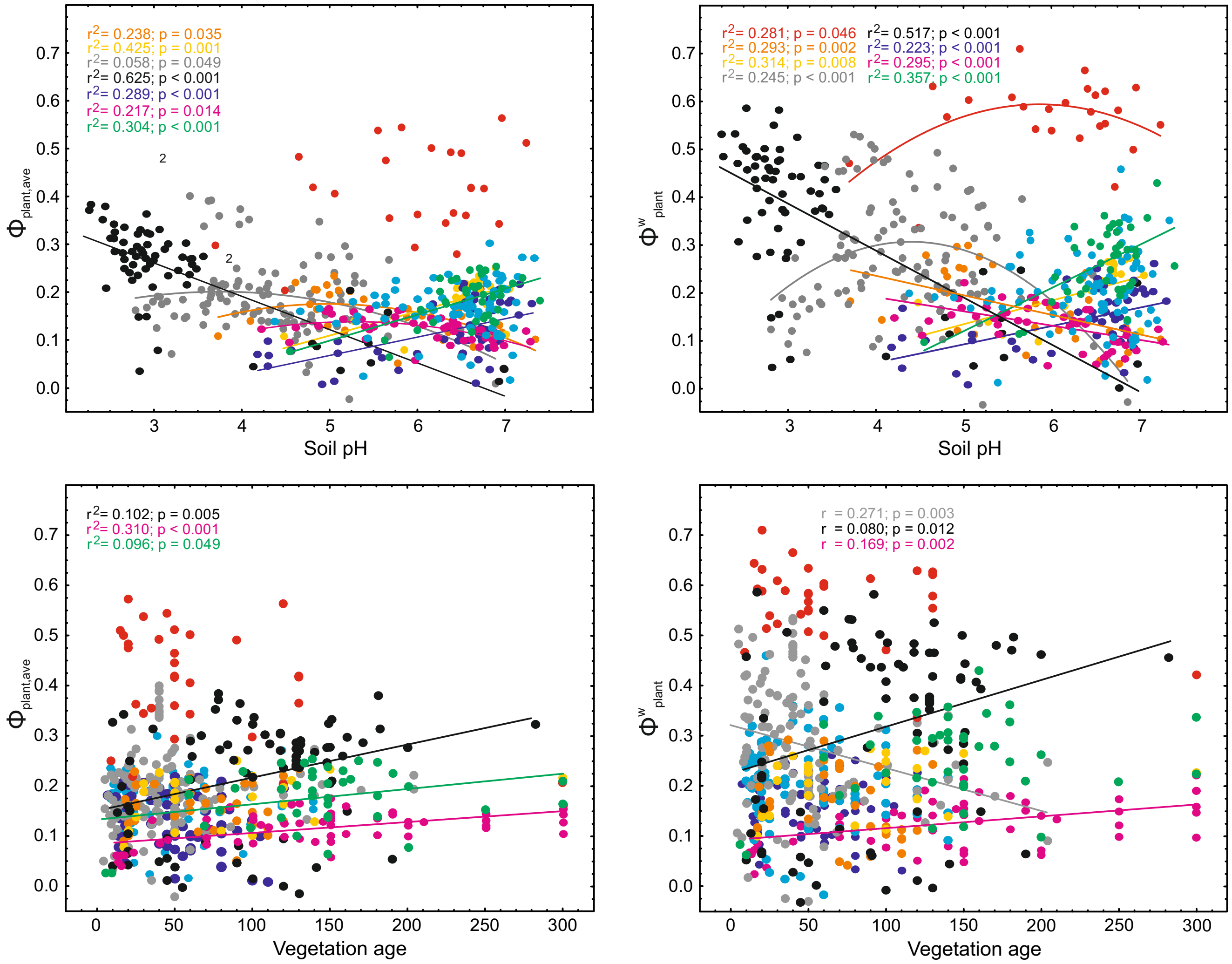

FIGURE S9 Relative effects of soil pH (top panels) and vegetation age (bottom panels) on the average partner preference in monocultures using unweighted ($\Phi_{plant,ave}$, left panels) and weighted (Φ^{W}_{plant}, right panels) values of focal host tree genera (red, *Alnus;* orange, *Betula;* yellow, *Corylus;* grey, *Picea;* black, *Pinus;* dark blue, *Populus;* magenta, *Quercus;* light blue, *Salix;* green, *Tilia).* Only significant linear and second-order polynomial regressions are indicated.
