# Supplementary material for: The influence of tree genus, phylogeny, and richness on the specificity, rarity, and diversity of ectomycorrhizal fungi
Source: Environ Microbiol Rep. 2024 Apr 4;16(2):e13253. doi: 10.1111/1758-2229.13253 (PMC10994715; doi:10.1111/1758-2229.13253)
Supplement: Supplementary file 10 — FIGURE S10. Distribution of tree species in the soil pH gradient. Only monocultures (here, at least 95% relative abundance of all EcM plants) are indicated, with grey bars indicating focal tree genera and blue bars depicting all tree genera. The scale corresponds to the focal tree genus. [file EMI4-16-e13253-s018.pdf]

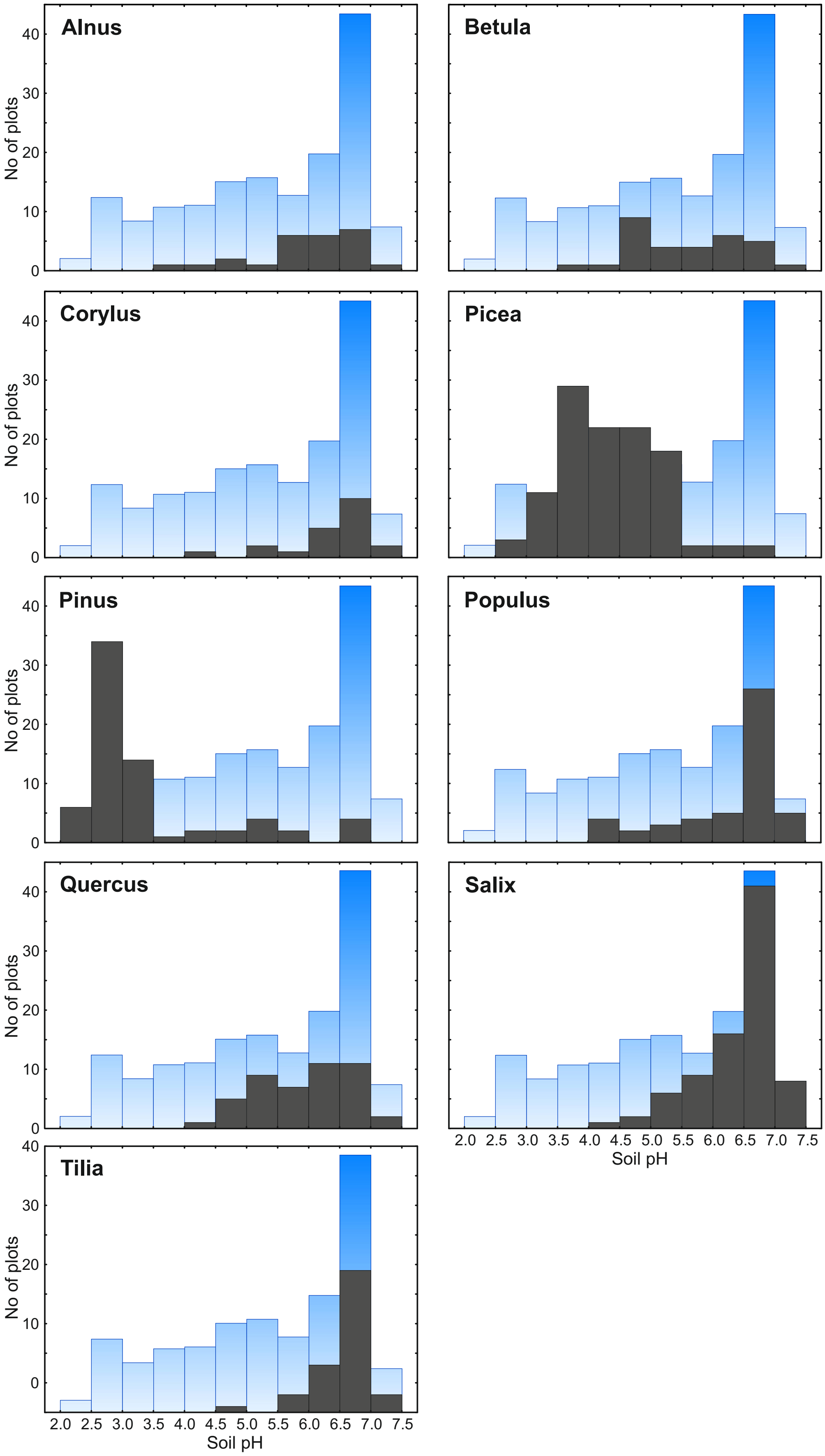

FIGURE S10 Distribution of tree species in the soil pH gradient. Only monocultures (here, at least 95% relative abundance of all EcM plants) are indicated, with grey bars indicating focal tree genera and blue bars depicting all tree genera. The scale corresponds to the focal tree genus.
