# Supplementary material for: The influence of tree genus, phylogeny, and richness on the specificity, rarity, and diversity of ectomycorrhizal fungi
Source: Environ Microbiol Rep. 2024 Apr 4;16(2):e13253. doi: 10.1111/1758-2229.13253 (PMC10994715; doi:10.1111/1758-2229.13253)
Supplement: Supplementary file 11 — FIGURE S11. Rarity in ectomycorrhizal symbiosis: (A) The effect of tree genus on the proportion of rare species; (B) The effect of soil pH on the proportion of rare species; (C) Relationship between fungal species frequency and soil pH range based on the quartiles (i.e., niche breadth). For the quartile‐based calculation, species with occurrence in <4 sites were excluded. Note the logarithmic scale in the x‐axis. [file EMI4-16-e13253-s005.pdf]

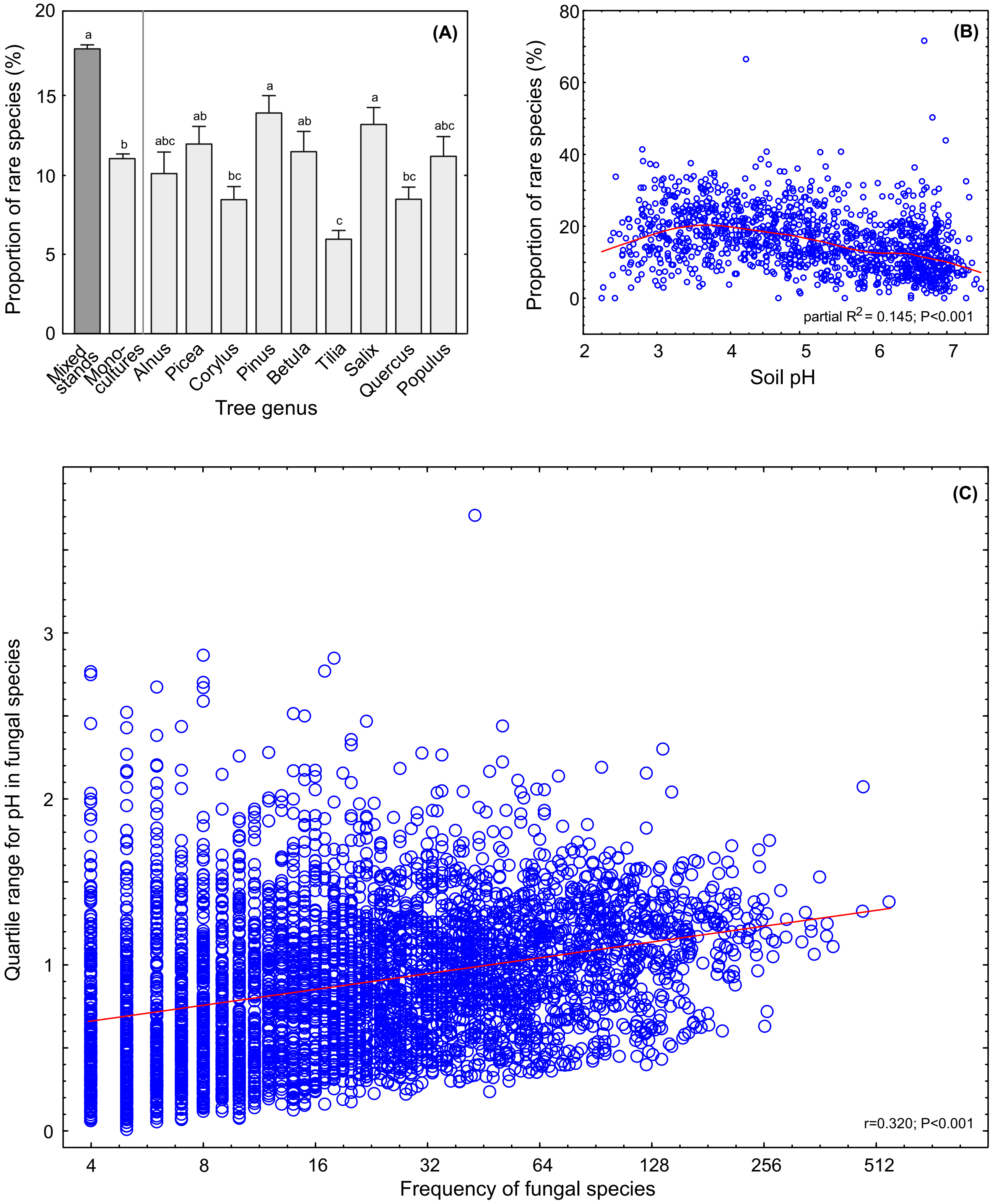

FIGURE S11 Rarity in ectomycorrhizal symbiosis: (A) The effect of tree genus on the proportion of rare species; (B) The effect of soil pH on the proportion of rare species; (C) Relationship between fungal species frequency and soil pH range based on the quartiles (i.e., niche breadth). For the quartile-based calculation, species with occurrence in <4 sites were excluded. Note the logarithmic scale in the x-axis.
