# Supplementary material for: The influence of tree genus, phylogeny, and richness on the specificity, rarity, and diversity of ectomycorrhizal fungi
Source: Environ Microbiol Rep. 2024 Apr 4;16(2):e13253. doi: 10.1111/1758-2229.13253 (PMC10994715; doi:10.1111/1758-2229.13253)
Supplement: Supplementary file 12 — FIGURE S12. Relationship between partner specificity and commonness of ectomycorrhizal fungi as based on (A) and (B), the R max risk ratio metric, and (C) and (D), the Blüthgen d’ interaction specialization metric, using species frequency (A) and (C) and total abundance (number of reads; (B) and (D) as proxies for commonness. Note the logarithmic scale in the abundance plots. We suspect that the Blüthgen d’ metric is inherently positively related to frequency of observations for uncommon species (present in 1–10 samples). [file EMI4-16-e13253-s019.pdf]

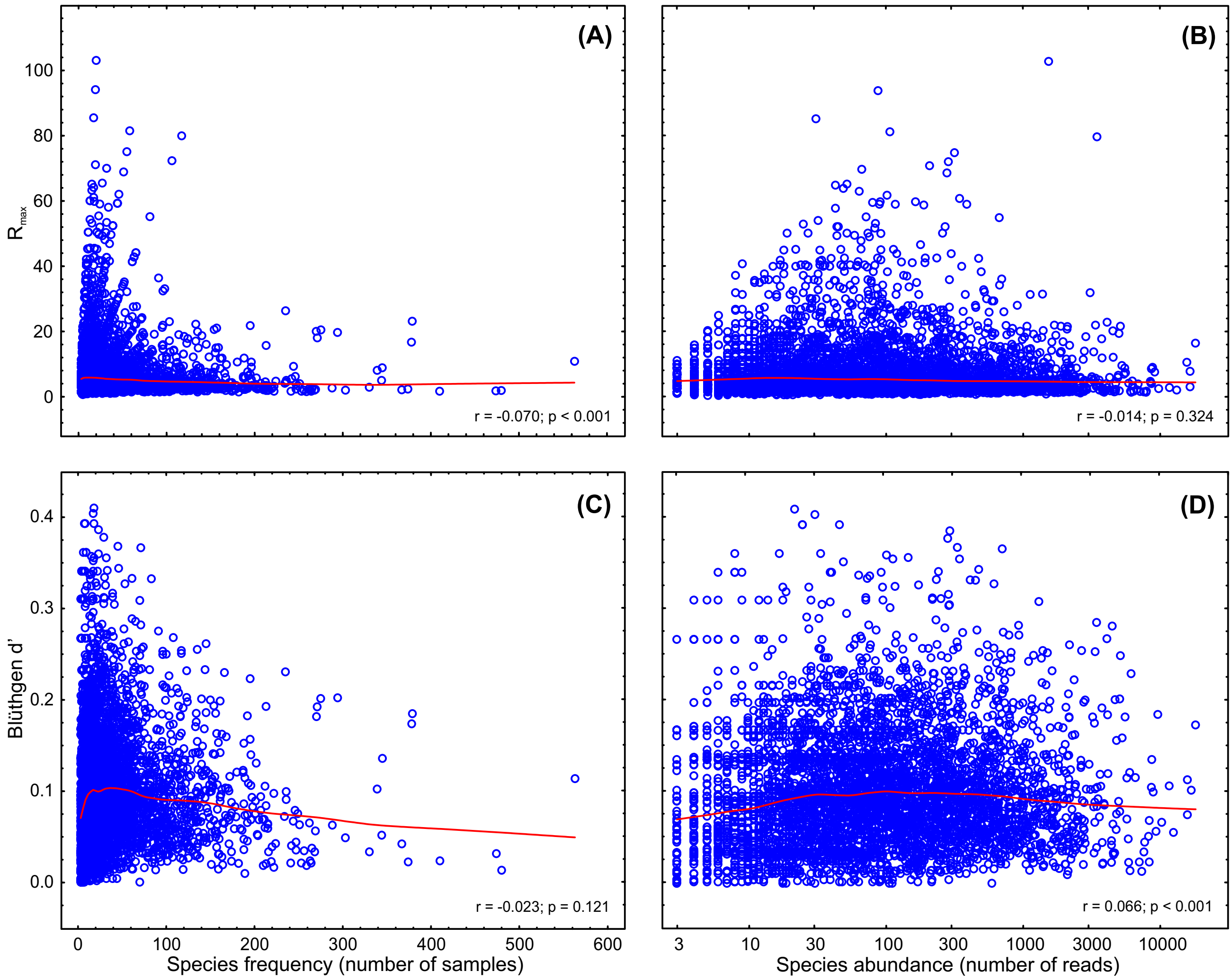

FIGURE S12 Relationship between partner specificity and commonness of ectomycorrhizal fungi as based on (A) and (B), the R_{max} risk ratio metric, and (C) and (D), the Blüthgen d' interaction specialisation metric, using species frequency ((A) and (C)) and total abundance (number of reads; (B) and (D)) as proxies for commonness. Note the logarithmic scale in the abundance plots. We suspect that the Blüthgen d' metric is inherently positively related to frequency of observations for uncommon species (present in 1-10 samples).
