# Supplementary material for: The influence of tree genus, phylogeny, and richness on the specificity, rarity, and diversity of ectomycorrhizal fungi
Source: Environ Microbiol Rep. 2024 Apr 4;16(2):e13253. doi: 10.1111/1758-2229.13253 (PMC10994715; doi:10.1111/1758-2229.13253)
Supplement: Supplementary file 13 — FIGURE S13. Relative abundance of trees in Estonia: (A) relative volume of tree genera; (B) relative area covered by monocultures (defined here as at least 95% of volume belonging to a single tree species; corresponds to 17.8% of the entire state forest area); (C) the lack of significant relationship between volume‐based tree abundance and average Φmax,ave values for each host. The tree abundance data are taken from the Estonian Forestry Register (https://register.metsad.ee). [file EMI4-16-e13253-s001.pdf]

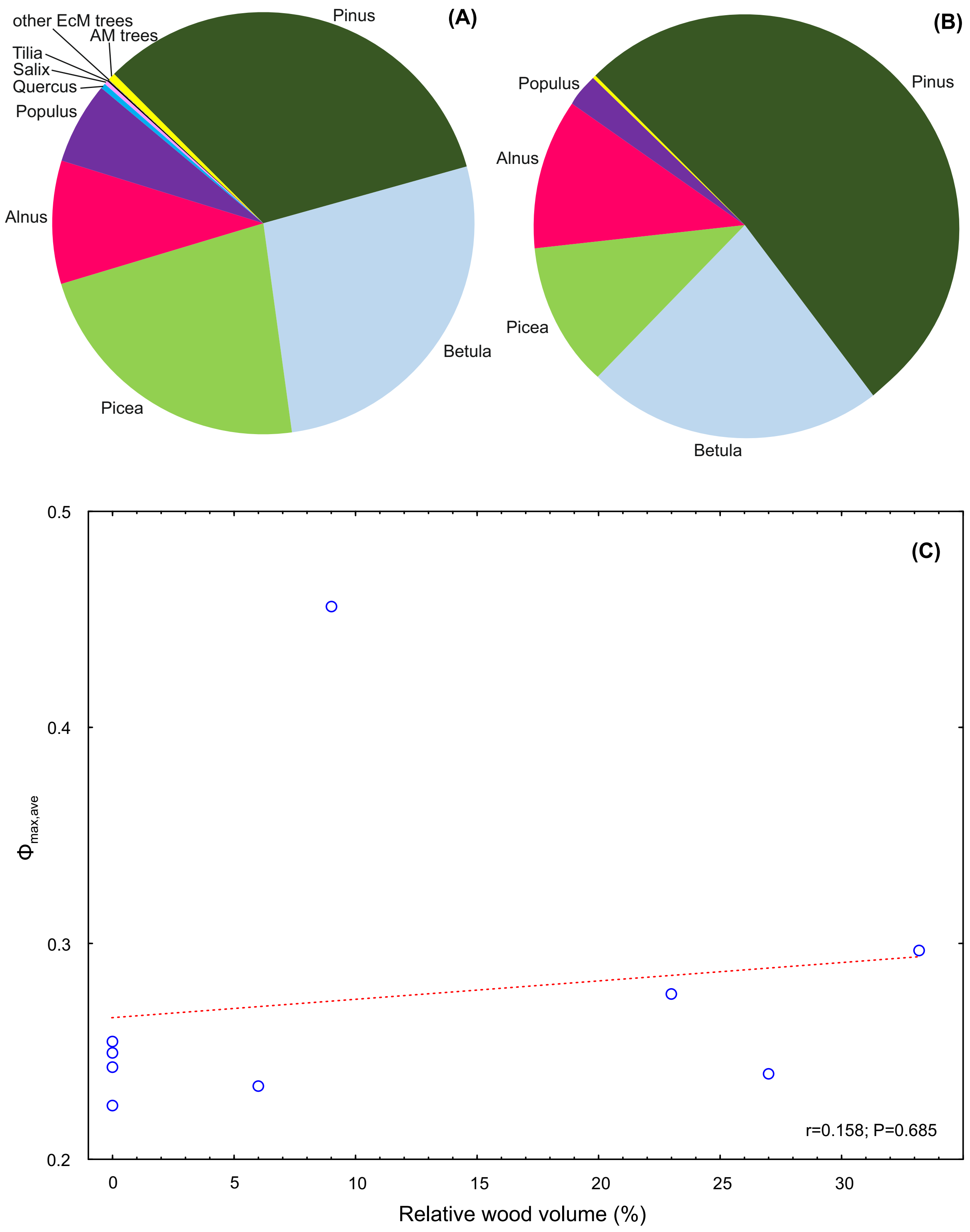

FIGURE S13 Relative abundance of trees in Estonia: (A) relative *volume* of tree genera; (B) relative *area* covered by monocultures (defined here as at least 95% of volume belonging to a single tree species; corresponds to 17.8% of the entire state forest area); (C) the lack of significant relationship between volume-based tree abundance and average $\Phi_{max,ave}$ values for each host. The tree abundance data is taken from the Estonian Forestry Register (https://register.metsad.ee).
