# Supplementary material for: The influence of tree genus, phylogeny, and richness on the specificity, rarity, and diversity of ectomycorrhizal fungi
Source: Environ Microbiol Rep. 2024 Apr 4;16(2):e13253. doi: 10.1111/1758-2229.13253 (PMC10994715; doi:10.1111/1758-2229.13253)
Supplement: Supplementary file 14 — FIGURE S14. Distance‐decay relationships among monospecific plots and all plots taken together (large panel) based on Bray–Curtis dissimilarity measure. In the extra graphs for Populus and Tilia, distance‐decay relationships were calculated separately for strong indicators of the target genus (red and magenta symbols), weak indicators of the target genus (orange symbols) and non‐indicators (black symbols). Lowess curves depict non‐linearity of these relationships. Statistics indicate linear Pearson correlations. Note that the relatively low distance decay across all plots is related to the lack of accounting for tree species and environment. [file EMI4-16-e13253-s002.pdf]

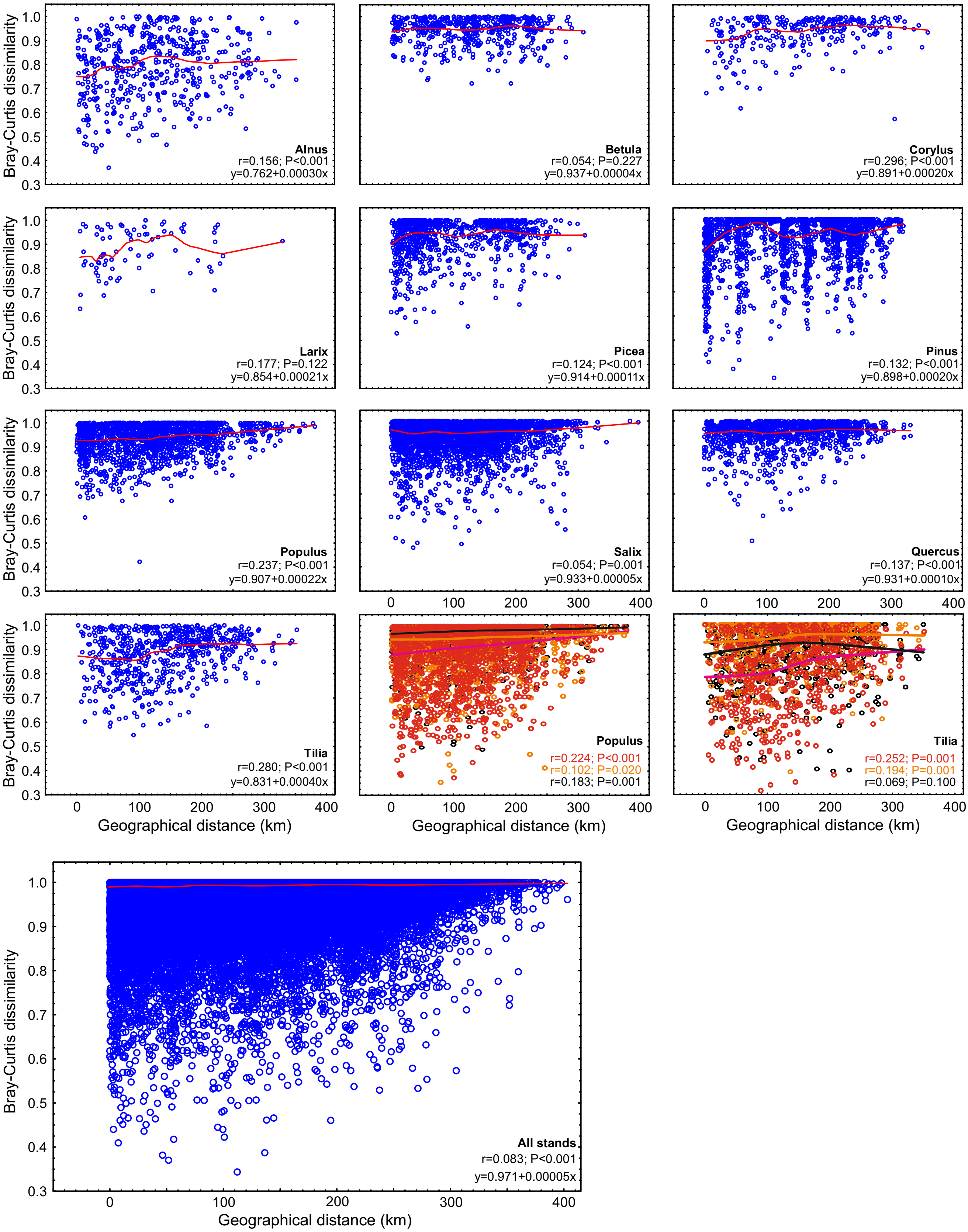

FIGURE S14 Distance-decay relationships among monospecific plots and all plots taken together (large panel) based on Bray-Curtis dissimilarity measure. In the extra graphs for Populus and Tilia, distance-decay relationships were calculated separately for strong indocators of the target genus (red and magenta symbols), weak indicators of the target genus (orange symbols) and non-indicators (black symbols). Lowess curves depict non-linearity of these relationships. Statistics indicate linear Pearson correlations. Note that the relatively low distance decay across all plots is related to the lack of accounting for tree species and environment.
