# Supplementary material for: The influence of tree genus, phylogeny, and richness on the specificity, rarity, and diversity of ectomycorrhizal fungi
Source: Environ Microbiol Rep. 2024 Apr 4;16(2):e13253. doi: 10.1111/1758-2229.13253 (PMC10994715; doi:10.1111/1758-2229.13253)
Supplement: Supplementary file 15 — FIGURE S15. Relationships of (A) ectomycorrhizal plant richness and (B) ectomycorrhizal fungal richness with Pielou evenness index, magenta symbols) and dominance (proportional abundance of the most common species, black symbols). Note the logarithmic scale in (A). [file EMI4-16-e13253-s013.pdf]

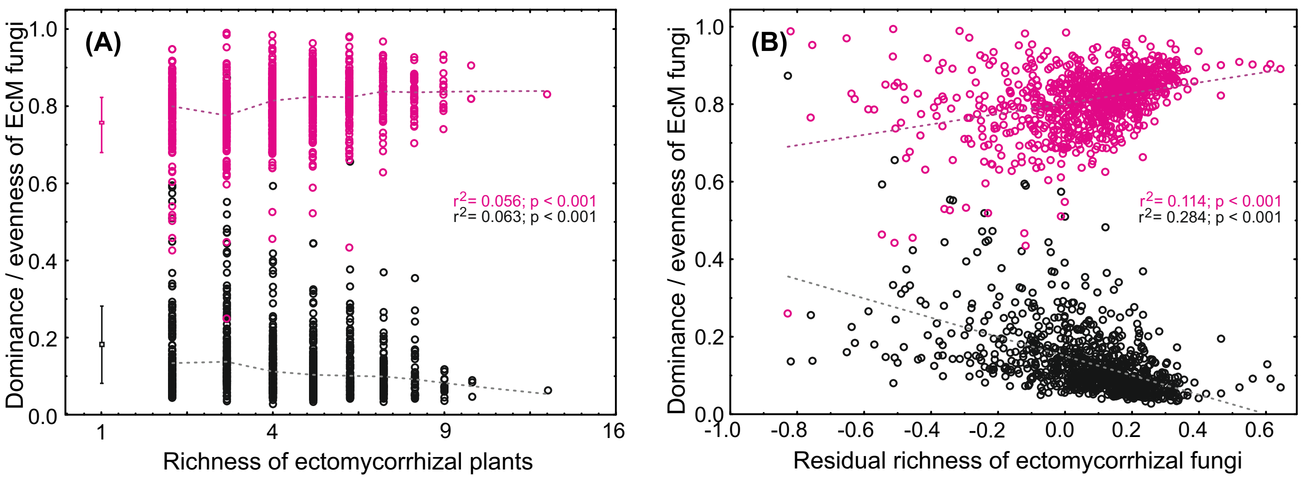

FIGURE S15 Relationships of (A) ectomycorrhizal plant richness and (B) ectomycorrhizal fungal richness with Pielou evenness index, magenta symbols) and dominance (proportional abundance of the most common species, black symbols). Note the logarithmic scale in (A).
