# Supplementary material for: The influence of tree genus, phylogeny, and richness on the specificity, rarity, and diversity of ectomycorrhizal fungi
Source: Environ Microbiol Rep. 2024 Apr 4;16(2):e13253. doi: 10.1111/1758-2229.13253 (PMC10994715; doi:10.1111/1758-2229.13253)
Supplement: Supplementary file 17 — TABLE S2. Samples used in the richness analysis. Additional metadata and OTU distribution are given in the datasets stored in the PlutoF repository. [file EMI4-16-e13253-s008.pdf]

**TABLE S2** Samples used in the richness analysis. Additional metadata and OTU distribution are given in the datasets stored in the PlutoF repository.

| Sample | Dominant plant species | Nativeness | Latitude | Longitude | Sampling time |
|---|---|---|---|---|---|
| G2536 | mixed | ND[1] | 58.46239 | 26.82084 | 2011-07-21 |
| G2537 | mixed | ND | 58.19075 | 26.57175 | 2011-07-21 |
| G2543 | mixed | ND | 58.27508 | 27.3247 | 2011-07-27 |
| G2541 | mixed | ND | 58.27711 | 27.32472 | 2011-07-27 |
| G2542 | mixed | ND | 58.27644 | 27.32429 | 2011-07-27 |
| G2544 | mixed | ND | 58.27423 | 27.32412 | 2011-07-27 |
| G2548 | mixed | ND | 58.27117 | 27.32785 | 2011-07-29 |
| G2545 | mixed | ND | 58.27515 | 27.32349 | 2011-07-29 |
| G2549 | mixed | ND | 58.36562 | 25.58797 | 2011-07-31 |
| G2555 | mixed | ND | 58.27624 | 27.32687 | 2011-08-01 |
| G2551 | mixed | ND | 58.28328 | 27.32476 | 2011-08-01 |
| G2552 | mixed | ND | 58.28402 | 27.32152 | 2011-08-01 |
| G2554 | mixed | ND | 58.27797 | 27.32377 | 2011-08-01 |
| G2553 | mixed | ND | 58.27779 | 27.32098 | 2011-08-01 |
| G2550 | mixed | ND | 58.28339 | 27.32315 | 2011-08-01 |
| G2556 | mixed | ND | 58.26945 | 27.3335 | 2011-08-02 |
| G2559 | mixed | ND | 58.27285 | 27.33115 | 2011-08-02 |
| G2557 | mixed | ND | 58.276 | 27.33773 | 2011-08-02 |
| G2558 | mixed | ND | 58.27534 | 27.33476 | 2011-08-02 |
| G2562 | mixed | ND | 58.26206 | 27.3179 | 2011-08-03 |
| G2561 | mixed | ND | 58.27091 | 27.31735 | 2011-08-03 |
| G2563 | mixed | ND | 58.26203 | 27.31185 | 2011-08-03 |
| G2564 | mixed | ND | 58.26329 | 27.31156 | 2011-08-03 |
| G2565 | mixed | ND | 58.26353 | 27.30645 | 2011-08-03 |
| G2567 | mixed | ND | 58.27957 | 27.31049 | 2011-08-08 |
| G2568 | mixed | ND | 58.28031 | 27.31255 | 2011-08-08 |
| G2566 | mixed | ND | 58.28122 | 27.29928 | 2011-08-08 |
| G2570 | mixed | ND | 58.2704 | 27.30979 | 2011-08-08 |
| G2569 | mixed | ND | 58.27645 | 27.31041 | 2011-08-08 |
| G2571 | mixed | ND | 58.29075 | 27.32895 | 2011-08-09 |
| G2573 | mixed | ND | 58.29013 | 27.32669 | 2011-08-09 |
| G2574 | mixed | ND | 58.289 | 27.31072 | 2011-08-09 |
| G2576 | mixed | ND | 58.27306 | 27.31827 | 2011-08-09 |
| G2575 | mixed | ND | 58.27111 | 27.31184 | 2011-08-09 |
| G2577 | mixed | ND | 58.26895 | 27.32285 | 2011-08-10 |
| G2578 | mixed | ND | 58.28548 | 27.36019 | 2011-08-10 |
| G2580 | mixed | ND | 58.27872 | 27.32624 | 2011-08-10 |
| G2581 | mixed | ND | 58.27893 | 27.32733 | 2011-08-10 |
| G2579 | mixed | ND | 58.28426 | 27.33501 | 2011-08-10 |
| G2620 | mixed | ND | 58.27961 | 26.3716 | 2011-08-20 |
| G2616 | mixed | ND | 58.26102 | 27.30141 | 2011-08-25 |
| G2629 | mixed | ND | 58.04579 | 24.71194 | 2011-08-30 |

| | | | | | |
|---|---|---|---|---|---|
| G2634 | mixed | ND | 58.33669 | 26.55972 | 2011-09-15 |
| S208 | Tilia_cordata | native | 58.15685 | 22.5004 | 2012-05-17 |
| S209 | mixed | ND | 58.49267 | 22.52195 | 2012-05-18 |
| G2755X | Pinus_sylvestris | native | 58.38859 | 22.65545 | 2012-06-28 |
| G2757X | mixed | ND | 58.01829 | 22.17463 | 2012-06-29 |
| G2767 | mixed | ND | 58.30937 | 22.07618 | 2012-06-30 |
| G2788 | mixed | ND | 58.30223 | 22.0958 | 2012-07-01 |
| G2797 | mixed | ND | 58.61218 | 22.80056 | 2012-07-02 |
| G2819 | Quercus_robur | native | 58.23408 | 22.44107 | 2012-07-04 |
| G2830 | mixed | ND | 58.58463 | 23.57021 | 2012-07-17 |
| G2829 | Tilia_cordata | native | 58.55674 | 23.54882 | 2012-07-17 |
| G2831 | Quercus_robur | native | 58.62038 | 24.10171 | 2012-07-18 |
| G2832 | Tilia_cordata | native | 58.24419 | 26.57518 | 2012-07-21 |
| G2844 | mixed | ND | 58.08112 | 26.34164 | 2012-09-11 |
| G2846 | mixed | ND | 58.89269 | 23.68405 | 2012-09-25 |
| G2847 | mixed | ND | 58.92812 | 23.86384 | 2012-09-26 |
| G2848 | mixed | ND | 58.43918 | 26.71262 | 2012-10-03 |
| G2971 | mixed | ND | 59.42275 | 24.6661 | 2012-10-24 |
| 45MR | mixed | ND | 58.25645 | 26.93328 | 2012-11-26 |
| 46MR | mixed | ND | 58.254 | 26.82118 | 2012-11-26 |
| 50MR | mixed | ND | 57.91666 | 27.18333 | 2013-04-24 |
| 40MR | mixed | ND | 58.1523 | 27.04826 | 2013-04-25 |
| 48MR | mixed | ND | 58.15738 | 27.04865 | 2013-04-25 |
| 47MR | mixed | ND | 57.92115 | 27.1859 | 2013-04-25 |
| 12MR | Picea_abies | native | 58.23692 | 26.48778 | 2013-04-25 |
| 43MR | mixed | ND | 58.21791 | 26.8386 | 2013-05-03 |
| 44MR | mixed | ND | 58.12398 | 26.62547 | 2013-05-03 |
| 49MR | mixed | ND | 58.13583 | 26.63528 | 2013-05-03 |
| 30MK | Picea_abies | native | 58.30203 | 26.66543 | 2013-05-03 |
| 41MR | mixed | ND | 58.27056 | 27.33336 | 2013-05-14 |
| S225 | Picea_abies | native | 58.3701 | 26.5561 | 2013-05-20 |
| S226 | mixed | ND | 58.4176 | 26.588 | 2013-05-21 |
| 19MR | mixed | ND | 58.26985 | 27.28958 | 2013-05-28 |
| 42MR | mixed | ND | 58.26763 | 27.29163 | 2013-05-29 |
| 27MR | mixed | ND | 58.18852 | 26.93822 | 2013-06-04 |
| 33MR | mixed | ND | 58.24941 | 26.92941 | 2013-06-04 |
| 39MR | mixed | ND | 58.24765 | 26.94472 | 2013-06-04 |
| 21MR | mixed | ND | 57.68446 | 26.87286 | 2013-06-05 |
| 25MR | mixed | ND | 58.1678 | 27.04318 | 2013-06-05 |
| 22MR | mixed | ND | 57.9108 | 27.32285 | 2013-06-05 |
| 2MR | mixed | ND | 58.15225 | 27.0454 | 2013-06-05 |
| 38MR | mixed | ND | 57.96941 | 27.41925 | 2013-06-05 |
| 29MR | mixed | ND | 58.13208 | 27.02621 | 2013-06-05 |
| 31MK | Pinus_sylvestris | native | 57.83018 | 26.89338 | 2013-06-05 |
| 17MR | mixed | ND | 59.01842 | 26.89503 | 2013-06-07 |
| 23MR | mixed | ND | 59.46488 | 25.75728 | 2013-06-07 |

| | | | | | |
|---|---|---|---|---|---|
| 34MR | mixed | ND | 59.07132 | 26.34942 | 2013-06-07 |
| 35MR | mixed | ND | 58.65855 | 26.95203 | 2013-06-07 |
| 14MR | mixed | ND | 59.21678 | 25.1753 | 2013-06-07 |
| 3MR | mixed | ND | 59.23448 | 24.8882 | 2013-06-07 |
| 32MR | mixed | ND | 59.24056 | 25.29783 | 2013-06-07 |
| 15MR | Pinus_sylvestris | native | 59.08153 | 27.16693 | 2013-06-07 |
| 18MR | Pinus_sylvestris | native | 59.13568 | 27.4421 | 2013-06-07 |
| 7MR | mixed | ND | 57.8267 | 26.40403 | 2013-06-11 |
| 10MR | mixed | ND | 58.01056 | 26.76935 | 2013-06-11 |
| 16MR | mixed | ND | 57.82707 | 26.40368 | 2013-06-11 |
| 6MR | mixed | ND | 58.09323 | 26.79088 | 2013-06-11 |
| 11MR | mixed | ND | 57.91195 | 26.31798 | 2013-06-11 |
| 24MR | Picea_abies | native | 58.06516 | 26.67997 | 2013-06-11 |
| 5MR | Picea_abies | native | 57.99168 | 26.67145 | 2013-06-11 |
| 8MK | Picea_abies | native | 57.9523 | 26.64025 | 2013-06-11 |
| G3224 | mixed | ND | 58.28062 | 22.81871 | 2013-06-12 |
| G3232 | Pinus_sylvestris | native | 58.21082 | 22.31476 | 2013-06-13 |
| G3242 | mixed | ND | 59.49325 | 25.36253 | 2013-06-15 |
| 1MR | mixed | ND | 58.15621 | 27.13176 | 2013-06-19 |
| 26MR | mixed | ND | 58.25496 | 26.57166 | 2013-06-19 |
| 4MR | mixed | ND | 58.15376 | 27.12508 | 2013-06-19 |
| 13MR | mixed | ND | 58.23698 | 26.48628 | 2013-06-19 |
| 36MR | Pinus_sylvestris | native | 58.2451 | 26.95928 | 2013-06-19 |
| S336 | Picea_abies | native | 58.3701 | 26.5561 | 2013-06-25 |
| IH.K19 | mixed | ND | 57.6415 | 26.4149 | 2013-06-28 |
| IH.K17 | Pinus_sylvestris | native | 57.6457 | 26.4148 | 2013-06-28 |
| IH.K29 | Pinus_sylvestris | native | 57.6898 | 26.4768 | 2013-06-29 |
| 28MR | mixed | ND | 57.54538 | 26.54443 | 2013-06-30 |
| 9MR | mixed | ND | 57.58672 | 26.68675 | 2013-06-30 |
| 20MR | Pinus_sylvestris | native | 57.58005 | 26.44685 | 2013-06-30 |
| IH.K16 | mixed | ND | 57.6652 | 26.4683 | 2013-07-01 |
| IH.K21 | mixed | ND | 57.6541 | 26.4972 | 2013-07-02 |
| G2745X | mixed | ND | 58.32669 | 21.89557 | 2013-07-02 |
| S227 | Corylus_avellana | native | 58.13673 | 25.54067 | 2013-07-02 |
| IH.K37 | Pinus_sylvestris | native | 57.6655 | 26.5061 | 2013-07-02 |
| G3247 | Corylus_avellana | native | 58.35784 | 22.47637 | 2013-07-03 |
| IH.K10 | Pinus_sylvestris | native | 57.6764 | 26.5529 | 2013-07-03 |
| IH.K35 | Pinus_sylvestris | native | 57.671 | 26.5578 | 2013-07-03 |
| IH.K05 | mixed | ND | 57.6823 | 26.6012 | 2013-07-04 |
| IH.K01 | Pinus_sylvestris | native | 57.6824 | 26.5988 | 2013-07-04 |
| IH.K02 | Pinus_sylvestris | native | 57.6726 | 26.5691 | 2013-07-05 |
| IH.K07 | Pinus_sylvestris | native | 57.6669 | 26.6047 | 2013-07-05 |
| IH.K24 | Pinus_sylvestris | native | 57.6687 | 26.4676 | 2013-07-05 |
| IH.K13 | mixed | ND | 57.6567 | 26.4493 | 2013-07-06 |
| IH.M10 | Pinus_sylvestris | native | 57.9409 | 27.3497 | 2013-07-09 |
| IH.M14 | mixed | ND | 57.9212 | 27.3791 | 2013-07-10 |

| | | | | | |
|---|---|---|---|---|---|
| S228 | Betula_pendula | native | 58.35203 | 26.60463 | 2013-07-10 |
| IH.M13 | Pinus_sylvestris | native | 57.9381 | 27.4035 | 2013-07-10 |
| IH.M07 | Pinus_sylvestris | native | 57.9246 | 27.3258 | 2013-07-11 |
| IH.M09 | Pinus_sylvestris | native | 57.9374 | 27.3465 | 2013-07-11 |
| IH.M27 | Pinus_sylvestris | native | 57.9384 | 27.299 | 2013-07-13 |
| IH.M33 | mixed | ND | 57.9504 | 27.3688 | 2013-07-14 |
| IH.M45 | mixed | ND | 57.9602 | 27.2862 | 2013-07-14 |
| IH.M23 | Pinus_sylvestris | native | 57.9608 | 27.2828 | 2013-07-14 |
| IH.M16 | mixed | ND | 57.9264 | 27.405 | 2013-07-15 |
| IH.M46 | mixed | ND | 57.9382 | 27.3527 | 2013-07-15 |
| IH.M30 | Pinus_sylvestris | native | 57.9557 | 27.3249 | 2013-07-19 |
| IH.M47 | Pinus_sylvestris | native | 57.9389 | 27.352 | 2013-07-20 |
| IH.M48 | Pinus_sylvestris | native | 57.9237 | 27.2948 | 2013-07-20 |
| IH.A61 | mixed | ND | 59.0579 | 27.5019 | 2013-07-24 |
| IH.A90 | mixed | ND | 59.0606 | 27.5044 | 2013-07-24 |
| IH.A12 | mixed | ND | 59.0773 | 27.4879 | 2013-07-25 |
| IH.A14 | Pinus_sylvestris | native | 59.0589 | 27.5083 | 2013-07-25 |
| IH.A39 | Pinus_sylvestris | native | 59.0619 | 27.5162 | 2013-07-27 |
| IH.A56 | Pinus_sylvestris | native | 59.0618 | 27.5112 | 2013-07-27 |
| IH.A01 | mixed | ND | 59.0673 | 27.5277 | 2013-07-27 |
| IH.A36 | Pinus_sylvestris | native | 59.0826 | 27.4557 | 2013-07-28 |
| IH.A51 | Pinus_sylvestris | native | 59.0771 | 27.4582 | 2013-07-28 |
| IH.A31 | Pinus_sylvestris | native | 59.1006 | 27.4371 | 2013-07-29 |
| IH.A89 | Pinus_sylvestris | native | 59.0972 | 27.4316 | 2013-07-29 |
| IH.A02 | mixed | ND | 59.096 | 27.4422 | 2013-07-29 |
| IH.A03 | mixed | ND | 59.0656 | 27.4805 | 2013-07-30 |
| IH.A21 | mixed | ND | 59.1001 | 27.4131 | 2013-07-30 |
| IH.A20 | Pinus_sylvestris | native | 59.1023 | 27.4188 | 2013-07-30 |
| IH.A93 | mixed | ND | 59.0729 | 27.4353 | 2013-07-31 |
| IH.A78 | mixed | ND | 59.0764 | 27.4764 | 2013-07-31 |
| S246 | Salix_pentandra | native | 58.42553 | 26.52957 | 2013-08-15 |
| S342 | mixed | ND | 58.4176 | 26.588 | 2013-08-16 |
| G3406 | Quercus_robur | native | 58.1478 | 25.0398 | 2013-08-25 |
| S281 | Quercus_robur | native | 58.379 | 26.706 | 2013-08-29 |
| S280 | Betula_pendula | native | 58.369 | 26.689 | 2013-08-30 |
| G3412 | Quercus_robur | native | 58.40102 | 26.74147 | 2013-09-05 |
| G3413 | Salix_caprea | native | 58.43891 | 26.89759 | 2013-09-05 |
| G3419 | Tilia_cordata | native | 58.31681 | 26.72325 | 2013-09-08 |
| G3430 | Salix_pentandra | native | 58.30027 | 22.5766 | 2013-09-08 |
| S337 | Picea_abies | native | 58.3701 | 26.5561 | 2013-09-16 |
| S343 | mixed | ND | 58.4176 | 26.588 | 2013-09-17 |
| G3426 | Betula_pendula | native | 58.35081 | 25.55063 | 2013-09-18 |
| G3429 | Quercus_robur | native | 58.47449 | 22.8236 | 2013-09-19 |
| S249 | Pinus_sylvestris | native | 57.87791 | 27.68945 | 2013-09-29 |
| G3449 | Quercus_robur | native | 58.09875 | 27.45895 | 2013-10-13 |
| S270 | Populus_tremula | native | 58.20379 | 26.80821 | 2014-05-01 |

| | | | | | |
|---|---|---|---|---|---|
| S338 | Picea_abies | native | 58.3701 | 26.5561 | 2014-05-06 |
| S344 | mixed | ND | 58.4176 | 26.588 | 2014-05-07 |
| G3518 | Quercus_robur | native | 59.4399 | 24.7935 | 2014-06-26 |
| S339 | Picea_abies | native | 58.3701 | 26.5561 | 2014-06-26 |
| S345 | mixed | ND | 58.4176 | 26.588 | 2014-06-27 |
| S272 | mixed | ND | 58.3325 | 21.9648 | 2014-07-01 |
| S273 | mixed | ND | 58.38 | 22.105 | 2014-07-01 |
| G3522 | Corylus_avellana | native | 58.4211 | 22.1522 | 2014-07-02 |
| S334 | Alnus_glutinosa | native | 57.95478 | 22.12285 | 2014-07-03 |
| S271 | Pinus_sylvestris | native | 59.3969 | 24.7292 | 2014-07-06 |
| S274 | Tilia_cordata | native | 57.9354 | 27.0355 | 2014-07-19 |
| G3527 | Tilia_cordata | native | 58.3457 | 23.9901 | 2014-07-25 |
| G3529 | Salix_pentandra | native | 58.335 | 24.285 | 2014-07-25 |
| G3531 | Salix_caprea | native | 58.3827 | 24.3231 | 2014-07-25 |
| S278 | Tilia_cordata | native | 58.8037 | 23.00227 | 2014-08-02 |
| S289 | mixed | ND | 59.01972 | 26.2319 | 2014-08-09 |
| G3541 | mixed | ND | 59.3994 | 24.2876 | 2014-08-15 |
| S340 | Picea_abies | native | 58.3701 | 26.5561 | 2014-08-15 |
| S346 | mixed | ND | 58.4176 | 26.588 | 2014-08-16 |
| G3546 | Quercus_robur | native | 59.4372 | 27.1851 | 2014-08-16 |
| G3544 | Alnus_incana | native | 59.4268 | 27.519 | 2014-08-16 |
| G3545 | Alnus_incana | native | 59.4269 | 27.5136 | 2014-08-16 |
| S276 | mixed | ND | 59.4641 | 24.4614 | 2014-08-20 |
| S277 | Alnus_incana | native | 58.0405 | 27.0525 | 2014-08-21 |
| G3547 | mixed | ND | 58.27905 | 27.32441 | 2014-08-23 |
| G3550 | mixed | ND | 58.41088 | 26.6008 | 2014-08-30 |
| G3552 | Salix_caprea | native | 58.3345 | 26.6617 | 2014-08-31 |
| G3556 | mixed | ND | 58.0374 | 26.5666 | 2014-09-12 |
| G3557 | mixed | ND | 58.0545 | 26.542 | 2014-09-12 |
| G3555 | Quercus_robur | native | 58.0443 | 26.4553 | 2014-09-12 |
| G3558 | Salix_caprea | native | 58.0629 | 26.9591 | 2014-09-12 |
| G3561 | mixed | ND | 57.977 | 26.784 | 2014-09-13 |
| G3560 | Quercus_robur | native | 57.91727 | 26.5767 | 2014-09-13 |
| G3559 | Salix_fragilis | introduced | 58.05987 | 26.9063 | 2014-09-13 |
| G3562 | Salix_fragilis | introduced | 58.0554 | 27.0601 | 2014-09-14 |
| G3567 | Salix_fragilis | introduced | 58.3875 | 26.5495 | 2014-09-14 |
| S341 | Picea_abies | native | 58.3701 | 26.5561 | 2014-09-16 |
| S347 | mixed | ND | 58.4176 | 26.588 | 2014-09-17 |
| G3565 | mixed | ND | 58.3245 | 26.5465 | 2014-09-20 |
| G3566 | Populus_x_wettsteinii | introduced | 58.3646 | 26.5182 | 2014-09-20 |
| G3563 | Salix_pentandra | native | 58.3402 | 26.6515 | 2014-09-20 |
| G3564 | Salix_caprea | native | 58.3078 | 26.5901 | 2014-09-20 |
| G3570 | Tilia_cordata | native | 58.5753 | 26.6848 | 2014-09-21 |
| G3569 | Quercus_robur | native | 58.52899 | 26.6385 | 2014-09-21 |
| G3571 | Salix_caprea | native | 58.57736 | 26.7223 | 2014-09-21 |
| G3572 | Picea_abies | native | 58.5301 | 26.7264 | 2014-09-21 |

| | | | | | |
|---|---|---|---|---|---|
| G3573 | mixed | ND | 57.179 | 24.844 | 2014-09-23 |
| G3574 | Betula_pendula | native | 57.1525 | 24.84865 | 2014-09-23 |
| G3575 | Betula_pendula | native | 56.9226 | 23.9848 | 2014-09-23 |
| G3577 | Fagus_sylvatica | introduced | 57.2431 | 22.6416 | 2014-09-24 |
| G3579 | Salix_caprea | native | 57.288 | 22.721 | 2014-09-24 |
| G3581 | mixed | ND | 57.6288 | 22.2878 | 2014-09-25 |
| G3585 | mixed | ND | 57.44429 | 22.64869 | 2014-09-25 |
| G3582 | mixed | ND | 57.6318 | 22.3927 | 2014-09-25 |
| G3589 | mixed | ND | 58.436 | 23.299 | 2014-09-27 |
| G3590 | Quercus_robur | native | 58.434 | 23.3011 | 2014-09-27 |
| G3593 | Populus_tremula | native | 58.5312 | 23.1117 | 2014-09-27 |
| G3595 | mixed | ND | 58.236 | 22.436 | 2014-09-28 |
| G3594 | mixed | ND | 58.3256 | 22.1404 | 2014-09-28 |
| G3600 | Corylus_avellana | native | 58.43088 | 22.8662 | 2014-09-28 |
| G3597 | Populus_tremula | native | 58.01 | 22.05 | 2014-09-28 |
| G3598 | Populus_tremula | native | 58.03 | 22.09 | 2014-09-28 |
| G3603 | mixed | ND | 57.6058 | 26.3519 | 2014-09-29 |
| G3601 | Tilia_cordata | native | 56.9705 | 24.1428 | 2014-09-29 |
| G3602 | Salix_pentandra | native | 57.45435 | 26.3477 | 2014-09-29 |
| G3605 | mixed | ND | 57.7368 | 27.0622 | 2014-10-11 |
| G3607 | mixed | ND | 57.948 | 27.207 | 2014-10-12 |
| S325 | mixed | ND | 58.36637 | 22.68792 | 2014-10-19 |
| G3609 | Alnus_glutinosa | native | 58.4709 | 26.7251 | 2014-10-20 |
| S326 | Salix_pentandra | native | 58.429 | 26.777 | 2014-10-20 |
| S327 | Salix_caprea | native | 58.4461 | 26.7493 | 2014-10-20 |
| G3608 | Picea_abies | native | 58.4422 | 26.7106 | 2014-10-20 |
| S328 | Betula_pendula | native | 58.33215 | 26.76782 | 2014-10-25 |
| S329 | Quercus_robur | native | 58.34965 | 26.76782 | 2014-10-25 |
| S331 | mixed | ND | 58.28881 | 26.635 | 2014-10-26 |
| S330 | mixed | ND | 58.32833 | 26.61229 | 2014-10-26 |
| S333 | Alnus_incana | native | 58.32802 | 26.81878 | 2014-10-29 |
| S332 | Salix_caprea | native | 58.2778 | 26.9858 | 2014-10-29 |
| G4004 | Quercus_robur | native | 58.47059 | 26.72608 | 2014-10-30 |
| G4005 | Salix_caprea | native | 58.35703 | 26.74306 | 2014-11-01 |
| S360 | Salix_fragilis | introduced | 58.3619 | 26.7492 | 2014-11-16 |
| S364 | mixed | ND | 58.669 | 23.69 | 2014-12-01 |
| S383 | Betula_pendula | native | 58.3889 | 26.7031 | 2014-12-20 |
| S382 | Corylus_avellana | native | 58.36755 | 26.69698 | 2014-12-20 |
| G4074 | mixed | ND | 58.08501 | 26.63799 | 2015-04-05 |
| G4081 | mixed | ND | 57.60641 | 26.96879 | 2015-04-18 |
| G4082 | mixed | ND | 57.70702 | 27.07193 | 2015-04-18 |
| G4078 | mixed | ND | 57.72607 | 27.22422 | 2015-04-18 |
| G4083 | mixed | ND | 57.74473 | 27.10667 | 2015-04-18 |
| G4080 | mixed | ND | 57.59164 | 27.05915 | 2015-04-18 |
| G4079 | mixed | ND | 57.58384 | 27.214 | 2015-04-18 |
| G4085 | mixed | ND | 58.21592 | 27.44006 | 2015-04-19 |

| | | | | | |
|---|---|---|---|---|---|
| G4084 | mixed | ND | 58.21251 | 27.47202 | 2015-04-19 |
| G4086 | mixed | ND | 58.22051 | 26.71692 | 2015-04-21 |
| G4088 | mixed | ND | 58.27148 | 26.73636 | 2015-04-21 |
| G4087 | Picea_abies | native | 58.17671 | 26.75998 | 2015-04-21 |
| G4089 | mixed | ND | 58.36555 | 26.55067 | 2015-04-26 |
| G4090 | mixed | ND | 58.43458 | 26.99315 | 2015-04-28 |
| G4091 | Tilia_cordata | native | 58.37949 | 27.04309 | 2015-04-28 |
| G4093 | mixed | ND | 58.65302 | 26.59739 | 2015-04-30 |
| G4092 | mixed | ND | 58.65107 | 26.62424 | 2015-04-30 |
| G4098 | mixed | ND | 58.68507 | 26.4643 | 2015-04-30 |
| G4094 | Quercus_robur | native | 58.73387 | 26.5282 | 2015-04-30 |
| G4097 | Corylus_avellana | native | 58.7816 | 26.50385 | 2015-04-30 |
| G4096 | Alnus_glutinosa | native | 58.73243 | 26.52714 | 2015-04-30 |
| G4095 | Salix_fragilis | introduced | 58.73288 | 26.52652 | 2015-04-30 |
| G4101 | mixed | ND | 57.72013 | 26.28092 | 2015-05-01 |
| G4100 | mixed | ND | 57.75778 | 26.30028 | 2015-05-01 |
| G4103 | mixed | ND | 57.61388 | 26.25787 | 2015-05-01 |
| G4099 | mixed | ND | 57.77012 | 26.30175 | 2015-05-01 |
| G4102 | mixed | ND | 57.59438 | 26.29983 | 2015-05-01 |
| G4104 | Salix_alba | introduced | 57.68283 | 26.20146 | 2015-05-01 |
| G4105 | Picea_abies | native | 57.70159 | 26.23994 | 2015-05-01 |
| G4109 | mixed | ND | 59.39403 | 27.84604 | 2015-05-04 |
| G4106 | mixed | ND | 59.34908 | 28.14477 | 2015-05-04 |
| G4111 | mixed | ND | 59.37509 | 27.86299 | 2015-05-04 |
| G4108 | mixed | ND | 59.41463 | 27.95951 | 2015-05-04 |
| G4107 | Tilia_cordata | native | 59.41076 | 27.97139 | 2015-05-04 |
| S516 | Picea_abies | native | 58.3701 | 26.5561 | 2015-05-04 |
| S496 | mixed | ND | 58.4176 | 26.588 | 2015-05-05 |
| S448 | mixed | ND | 58.566 | 23.211 | 2015-05-07 |
| S449 | mixed | ND | 58.551 | 23.0735 | 2015-05-07 |
| G4118 | mixed | ND | 58.56895 | 23.23048 | 2015-05-07 |
| G4119 | mixed | ND | 58.53566 | 23.08956 | 2015-05-07 |
| G4125 | mixed | ND | 58.56412 | 23.28072 | 2015-05-07 |
| S447 | mixed | ND | 58.609 | 23.173 | 2015-05-07 |
| G4115 | mixed | ND | 58.55604 | 23.27395 | 2015-05-07 |
| G4117 | Corylus_avellana | native | 58.61793 | 23.18202 | 2015-05-07 |
| G4126 | mixed | ND | 58.59361 | 23.17602 | 2015-05-08 |
| G4123 | mixed | ND | 58.67579 | 23.16582 | 2015-05-08 |
| G4124 | mixed | ND | 58.58693 | 23.28597 | 2015-05-08 |
| S446 | mixed | ND | 58.601 | 23.294 | 2015-05-08 |
| G4121 | mixed | ND | 58.59043 | 23.08258 | 2015-05-08 |
| G4122 | Corylus_avellana | native | 58.60097 | 23.09898 | 2015-05-08 |
| G4129 | mixed | ND | 58.55107 | 22.91708 | 2015-05-09 |
| G4131 | mixed | ND | 58.58903 | 22.85268 | 2015-05-09 |
| G4127 | Tilia_cordata | native | 58.53679 | 23.0413 | 2015-05-09 |
| G4132 | Tilia_cordata | native | 58.47591 | 22.78101 | 2015-05-09 |

| | | | | | |
|---|---|---|---|---|---|
| G4130 | Betula_pendula | native | 58.58002 | 22.84337 | 2015-05-09 |
| G4128 | Quercus_robur | native | 58.58311 | 22.9322 | 2015-05-09 |
| G4133 | mixed | ND | 58.35967 | 22.82632 | 2015-05-10 |
| G4136 | mixed | ND | 58.17031 | 24.955 | 2015-05-10 |
| G4134 | mixed | ND | 58.37815 | 22.9243 | 2015-05-10 |
| G4135 | Corylus_avellana | native | 58.40286 | 22.88935 | 2015-05-10 |
| S454 | mixed | ND | 58.476 | 25.5154 | 2015-05-26 |
| S453 | mixed | ND | 58.5601 | 25.5544 | 2015-05-26 |
| S451 | mixed | ND | 58.4594 | 26.0845 | 2015-05-26 |
| S452 | Quercus_robur | native | 58.4877 | 26.0504 | 2015-05-26 |
| G4154 | mixed | ND | 58.94185 | 25.44514 | 2015-05-27 |
| G4158 | mixed | ND | 58.80019 | 25.67248 | 2015-05-27 |
| G4156 | Tilia_cordata | native | 58.91272 | 25.61173 | 2015-05-27 |
| G4157 | Corylus_avellana | native | 58.89217 | 25.71693 | 2015-05-27 |
| G4155 | Salix_alba | introduced | 58.90255 | 25.57723 | 2015-05-27 |
| G4159 | mixed | ND | 58.31881 | 25.51712 | 2015-05-31 |
| G4166 | mixed | ND | 58.31913 | 25.7798 | 2015-05-31 |
| G4161 | mixed | ND | 58.29834 | 25.5007 | 2015-05-31 |
| G4162 | mixed | ND | 58.25145 | 25.48897 | 2015-05-31 |
| G4163 | mixed | ND | 58.20641 | 25.45762 | 2015-05-31 |
| G4160 | mixed | ND | 58.31679 | 25.49964 | 2015-05-31 |
| G4164 | Betula_pendula | native | 58.27206 | 25.62077 | 2015-05-31 |
| G4165 | Salix_caprea | native | 58.27242 | 25.68144 | 2015-05-31 |
| G4168 | mixed | ND | 58.97189 | 26.94217 | 2015-06-02 |
| G4170 | mixed | ND | 58.78374 | 26.77475 | 2015-06-02 |
| G4169 | Betula_pendula | native | 58.83692 | 26.80751 | 2015-06-02 |
| G4167 | Salix_pentandra | native | 58.92051 | 27.01118 | 2015-06-02 |
| G4171 | mixed | ND | 58.48766 | 26.22524 | 2015-06-04 |
| G4172 | mixed | ND | 58.54138 | 26.19709 | 2015-06-04 |
| G4173 | mixed | ND | 57.97677 | 25.92078 | 2015-06-07 |
| G4174 | mixed | ND | 57.86071 | 25.87761 | 2015-06-07 |
| G4176 | mixed | ND | 57.96317 | 25.71178 | 2015-06-07 |
| G4178 | mixed | ND | 58.03199 | 25.77246 | 2015-06-07 |
| G4175 | mixed | ND | 57.91811 | 25.87444 | 2015-06-07 |
| G4177 | mixed | ND | 58.00505 | 25.55198 | 2015-06-07 |
| G4179 | mixed | ND | 58.55342 | 27.00963 | 2015-06-10 |
| G4180 | mixed | ND | 58.55229 | 27.11187 | 2015-06-10 |
| G4181 | mixed | ND | 58.60784 | 27.11763 | 2015-06-10 |
| G4183 | mixed | ND | 58.10004 | 27.49112 | 2015-06-13 |
| G4184 | mixed | ND | 58.12625 | 27.52591 | 2015-06-13 |
| G4185 | Picea_abies | native | 58.10242 | 27.47875 | 2015-06-13 |
| G4188 | mixed | ND | 57.74963 | 27.46149 | 2015-06-14 |
| G4187 | mixed | ND | 57.70927 | 27.42154 | 2015-06-14 |
| G4186 | mixed | ND | 57.69097 | 27.35867 | 2015-06-14 |
| G4189 | Populus_tremula | native | 57.84444 | 27.20142 | 2015-06-14 |
| G4194 | mixed | ND | 58.69371 | 25.94653 | 2015-06-17 |

| | | | | | |
|---|---|---|---|---|---|
| G4192 | mixed | ND | 58.69936 | 25.89106 | 2015-06-17 |
| G4190 | Tilia_cordata | native | 58.62416 | 25.92107 | 2015-06-17 |
| G4191 | Tilia_cordata | native | 58.69443 | 25.73916 | 2015-06-17 |
| G4195 | Quercus_robur | native | 58.66293 | 26.08466 | 2015-06-17 |
| G4193 | Picea_abies | native | 58.70757 | 25.90916 | 2015-06-17 |
| G4196 | mixed | ND | 58.27992 | 25.23166 | 2015-06-21 |
| G4200 | mixed | ND | 58.47029 | 24.90098 | 2015-06-21 |
| G4201 | mixed | ND | 58.56521 | 25.27874 | 2015-06-21 |
| G4197 | mixed | ND | 58.45205 | 24.76821 | 2015-06-21 |
| G4199 | mixed | ND | 58.46025 | 24.77642 | 2015-06-21 |
| G4198 | Quercus_robur | native | 58.45005 | 24.76498 | 2015-06-21 |
| G4202 | Alnus_glutinosa | native | 59.51083 | 24.81622 | 2015-06-22 |
| G4203 | mixed | ND | 59.63416 | 25.76802 | 2015-06-23 |
| G4204 | mixed | ND | 59.63815 | 25.7568 | 2015-06-23 |
| G4209 | mixed | ND | 59.49752 | 25.08889 | 2015-06-23 |
| G4206 | mixed | ND | 59.47463 | 25.46561 | 2015-06-23 |
| G4207 | mixed | ND | 59.49261 | 25.28566 | 2015-06-23 |
| G4208 | mixed | ND | 59.47425 | 25.16384 | 2015-06-23 |
| G4210 | mixed | ND | 58.9642 | 24.04112 | 2015-06-24 |
| G4212 | mixed | ND | 58.83246 | 23.85741 | 2015-06-24 |
| G4214 | mixed | ND | 58.93124 | 24.18453 | 2015-06-24 |
| G4215 | mixed | ND | 58.95377 | 24.21012 | 2015-06-24 |
| G4213 | Tilia_cordata | native | 58.8548 | 23.96153 | 2015-06-24 |
| G4211 | Populus_tremula | native | 58.8231 | 23.8316 | 2015-06-24 |
| S517 | Picea_abies | native | 58.3701 | 26.5561 | 2015-06-25 |
| S497 | mixed | ND | 58.4176 | 26.588 | 2015-06-26 |
| G4216 | mixed | ND | 59.37045 | 24.59195 | 2015-06-26 |
| G4219 | mixed | ND | 59.32056 | 24.35037 | 2015-06-26 |
| G4220 | mixed | ND | 59.36552 | 24.1268 | 2015-06-26 |
| G4218 | Tilia_cordata | native | 59.46176 | 24.37443 | 2015-06-26 |
| G4222 | Tilia_cordata | native | 59.38461 | 24.03861 | 2015-06-26 |
| G4221 | Corylus_avellana | native | 59.3834 | 24.04384 | 2015-06-26 |
| G4224 | mixed | ND | 58.92665 | 24.39394 | 2015-06-27 |
| G4227 | mixed | ND | 58.67014 | 24.49917 | 2015-06-27 |
| G4225 | mixed | ND | 58.7675 | 24.24921 | 2015-06-27 |
| G4223 | Tilia_cordata | native | 58.92127 | 24.4496 | 2015-06-27 |
| G4226 | Betula_pendula | native | 58.66648 | 24.47796 | 2015-06-27 |
| G4229 | Quercus_robur | native | 58.88604 | 24.4728 | 2015-06-27 |
| G4228 | Populus_tremula | native | 58.77466 | 24.47113 | 2015-06-27 |
| G4230 | Tilia_cordata | native | 59.43324 | 24.73524 | 2015-06-28 |
| G4231 | Salix_caprea | native | 59.45245 | 24.85831 | 2015-06-28 |
| G4233 | mixed | ND | 59.09698 | 24.91857 | 2015-06-29 |
| G4236 | mixed | ND | 59.20217 | 24.8887 | 2015-06-29 |
| G4232 | mixed | ND | 59.10254 | 24.82091 | 2015-06-29 |
| G4234 | Betula_pendula | native | 59.04783 | 25.10548 | 2015-06-29 |
| G4235 | Picea_abies | native | 59.04865 | 25.02258 | 2015-06-29 |

| | | | | | |
|---|---|---|---|---|---|
| G4237 | mixed | ND | 58.18332 | 26.90637 | 2015-07-02 |
| G4238 | Betula_pendula | native | 58.39994 | 26.69266 | 2015-07-03 |
| G4242 | mixed | ND | 58.66776 | 26.76167 | 2015-07-04 |
| G4239 | mixed | ND | 58.9959 | 27.74103 | 2015-07-04 |
| G4240 | mixed | ND | 59.17464 | 27.79904 | 2015-07-04 |
| S457 | mixed | ND | 59.1386 | 27.81751 | 2015-07-04 |
| G4241 | mixed | ND | 59.1106 | 27.57847 | 2015-07-04 |
| S507 | mixed | ND | 58.04031 | 26.46058 | 2015-07-05 |
| G4245 | mixed | ND | 57.61513 | 26.6114 | 2015-07-05 |
| G4250 | mixed | ND | 57.63764 | 27.04034 | 2015-07-05 |
| G4244 | mixed | ND | 57.75937 | 26.57639 | 2015-07-05 |
| G4247 | mixed | ND | 57.58101 | 26.61423 | 2015-07-05 |
| G4249 | mixed | ND | 57.6387 | 26.9097 | 2015-07-05 |
| G4246 | mixed | ND | 57.58569 | 26.64614 | 2015-07-05 |
| G4243 | Tilia_cordata | native | 57.8628 | 26.61021 | 2015-07-05 |
| G4256 | mixed | ND | 58.47159 | 25.54663 | 2015-07-09 |
| G4255 | mixed | ND | 58.47737 | 25.4637 | 2015-07-09 |
| G4253 | mixed | ND | 58.39744 | 25.66688 | 2015-07-09 |
| G4251 | mixed | ND | 58.3913 | 25.74195 | 2015-07-09 |
| G4254 | Salix_caprea | native | 58.39614 | 25.6632 | 2015-07-09 |
| G4257 | Salix_fragilis | introduced | 58.36433 | 25.58592 | 2015-07-09 |
| G4258 | mixed | ND | 58.35593 | 27.28802 | 2015-07-11 |
| G4259 | mixed | ND | 58.28217 | 27.38359 | 2015-07-11 |
| S503 | mixed | ND | 58.49291 | 24.13659 | 2015-07-12 |
| G4260 | mixed | ND | 57.91466 | 27.69713 | 2015-07-12 |
| G4261 | mixed | ND | 57.94398 | 27.29372 | 2015-07-12 |
| S501 | mixed | ND | 58.50835 | 24.10847 | 2015-07-12 |
| S502 | mixed | ND | 58.50206 | 24.11465 | 2015-07-12 |
| S500 | mixed | ND | 58.548 | 23.9912 | 2015-07-12 |
| S506 | mixed | ND | 58.3972 | 24.09308 | 2015-07-13 |
| S504 | mixed | ND | 58.58833 | 24.35435 | 2015-07-13 |
| S505 | mixed | ND | 58.37978 | 24.05879 | 2015-07-13 |
| G4265 | mixed | ND | 58.04024 | 25.79569 | 2015-07-15 |
| G4267 | mixed | ND | 58.14075 | 25.68184 | 2015-07-15 |
| G4264 | mixed | ND | 58.10334 | 25.90432 | 2015-07-15 |
| G4266 | mixed | ND | 58.14011 | 25.69482 | 2015-07-15 |
| G4262 | Populus_tremula | native | 58.1045 | 26.00713 | 2015-07-15 |
| G4273 | mixed | ND | 58.91527 | 25.17961 | 2015-07-17 |
| G4271 | mixed | ND | 58.90765 | 24.93117 | 2015-07-17 |
| G4268 | mixed | ND | 59.0269 | 25.63589 | 2015-07-17 |
| G4272 | Alnus_incana | native | 58.90378 | 25.09558 | 2015-07-17 |
| G4270 | Populus_tremula | native | 58.99323 | 25.08443 | 2015-07-17 |
| G4274 | Betula_pendula | native | 57.94402 | 27.65129 | 2015-07-19 |
| G4276 | mixed | ND | 58.93485 | 23.58988 | 2015-07-20 |
| G4277 | mixed | ND | 59.07708 | 22.63511 | 2015-07-20 |
| G4280 | mixed | ND | 58.8653 | 22.93925 | 2015-07-20 |

| | | | | | |
|---|---|---|---|---|---|
| G4275 | mixed | ND | 58.94699 | 23.74408 | 2015-07-20 |
| G4278 | mixed | ND | 58.89158 | 22.99835 | 2015-07-21 |
| S468 | mixed | ND | 58.91431 | 22.67471 | 2015-07-21 |
| S471 | mixed | ND | 58.85375 | 22.56574 | 2015-07-21 |
| G4279 | mixed | ND | 58.88085 | 22.95802 | 2015-07-21 |
| G4283 | mixed | ND | 58.84292 | 23.04166 | 2015-07-21 |
| G4285 | mixed | ND | 58.80144 | 22.83915 | 2015-07-21 |
| G4282 | Quercus_robur | native | 58.86964 | 22.94076 | 2015-07-21 |
| G4284 | Corylus_avellana | native | 58.84627 | 22.99793 | 2015-07-21 |
| G4288 | mixed | ND | 58.9938 | 22.80058 | 2015-07-22 |
| S473 | mixed | ND | 58.7183 | 22.52558 | 2015-07-22 |
| S475 | mixed | ND | 58.71817 | 22.51345 | 2015-07-22 |
| G4289 | mixed | ND | 58.84069 | 22.78429 | 2015-07-22 |
| S472 | mixed | ND | 58.70136 | 22.50552 | 2015-07-22 |
| S474 | mixed | ND | 58.71806 | 22.51694 | 2015-07-22 |
| G4296 | mixed | ND | 58.7925 | 22.82919 | 2015-07-22 |
| S459 | mixed | ND | 58.78842 | 22.47252 | 2015-07-22 |
| G4291 | Helianthemum_nummularium | native | 58.76182 | 22.80332 | 2015-07-22 |
| S467 | mixed | ND | 58.92356 | 22.05616 | 2015-07-23 |
| S460 | mixed | ND | 58.9341 | 22.16529 | 2015-07-23 |
| S465 | mixed | ND | 58.89994 | 22.2581 | 2015-07-23 |
| S466 | mixed | ND | 58.92065 | 22.33186 | 2015-07-23 |
| G4297 | mixed | ND | 58.99143 | 22.43933 | 2015-07-23 |
| G4299 | Tilia_cordata | native | 58.98247 | 22.46456 | 2015-07-23 |
| G4300 | Alnus_glutinosa | native | 58.93359 | 22.39061 | 2015-07-23 |
| G4306 | mixed | ND | 58.69647 | 22.5162 | 2015-07-24 |
| S461 | mixed | ND | 58.83212 | 23.12336 | 2015-07-24 |
| S462 | mixed | ND | 58.83752 | 23.12283 | 2015-07-24 |
| S463 | mixed | ND | 58.75463 | 23.09454 | 2015-07-24 |
| S458 | mixed | ND | 58.9741 | 22.84718 | 2015-07-24 |
| S464 | mixed | ND | 58.98545 | 22.8584 | 2015-07-24 |
| S469 | mixed | ND | 58.97688 | 22.8645 | 2015-07-24 |
| G4301 | Betula_pendula | native | 58.83258 | 22.63346 | 2015-07-24 |
| G4305 | Pinus_sylvestris | native | 58.72023 | 22.46615 | 2015-07-24 |
| S476 | mixed | ND | 58.78311 | 23.04464 | 2015-07-25 |
| G4308 | mixed | ND | 58.74179 | 23.13487 | 2015-07-25 |
| G4309 | mixed | ND | 58.76592 | 23.08095 | 2015-07-25 |
| S477 | mixed | ND | 58.78039 | 23.04814 | 2015-07-25 |
| S478 | mixed | ND | 58.77486 | 23.04723 | 2015-07-25 |
| G4307 | Betula_pendula | native | 58.79553 | 23.00588 | 2015-07-25 |
| S479 | mixed | ND | 58.90898 | 23.63845 | 2015-07-26 |
| S480 | mixed | ND | 58.80407 | 25.22249 | 2015-07-26 |
| S481 | mixed | ND | 58.80102 | 25.26525 | 2015-07-26 |
| G4313 | mixed | ND | 58.72258 | 25.06363 | 2015-07-26 |
| G4311 | mixed | ND | 58.87539 | 24.74554 | 2015-07-26 |
| G4312 | mixed | ND | 58.69373 | 24.96776 | 2015-07-26 |

| | | | | | |
|---|---|---|---|---|---|
| G4310 | Quercus_robur | native | 58.9508 | 23.63036 | 2015-07-26 |
| S568 | mixed | ND | 59.25517 | 26.42818 | 2015-07-30 |
| S563 | mixed | ND | 59.20575 | 26.32442 | 2015-07-30 |
| S566 | Quercus_robur | native | 59.20778 | 26.4348 | 2015-07-30 |
| S508 | mixed | ND | 59.08257 | 23.73767 | 2015-08-02 |
| S509 | mixed | ND | 59.00297 | 23.48397 | 2015-08-02 |
| G4343 | mixed | ND | 58.12591 | 27.3317 | 2015-08-15 |
| G4344 | mixed | ND | 58.16686 | 27.30071 | 2015-08-15 |
| G4345 | mixed | ND | 57.73167 | 27.35911 | 2015-08-16 |
| G4348 | Quercus_robur | native | 57.84645 | 27.20106 | 2015-08-16 |
| G4347 | Alnus_incana | native | 57.75965 | 27.47665 | 2015-08-16 |
| G4346 | Picea_abies | native | 57.76052 | 27.47502 | 2015-08-16 |
| G4349 | mixed | ND | 58.63981 | 24.3248 | 2015-08-17 |
| G4350 | mixed | ND | 58.60927 | 24.18329 | 2015-08-17 |
| G4352 | mixed | ND | 58.6117 | 23.97482 | 2015-08-17 |
| G4351 | mixed | ND | 58.61721 | 24.03508 | 2015-08-17 |
| S498 | mixed | ND | 58.4176 | 26.588 | 2015-08-18 |
| G4353 | mixed | ND | 58.68618 | 23.69075 | 2015-08-18 |
| G4354 | Betula_pendula | native | 58.67603 | 23.533 | 2015-08-18 |
| S518 | Picea_abies | native | 58.3701 | 26.5561 | 2015-08-18 |
| G4355 | mixed | ND | 58.4647 | 23.8335 | 2015-08-19 |
| G4357 | mixed | ND | 58.54237 | 23.71942 | 2015-08-19 |
| G4356 | mixed | ND | 58.56201 | 23.72078 | 2015-08-19 |
| G4358 | mixed | ND | 58.38711 | 23.83884 | 2015-08-20 |
| G4359 | mixed | ND | 58.42663 | 23.69535 | 2015-08-20 |
| G4360 | Betula_pendula | native | 58.55383 | 23.59506 | 2015-08-20 |
| G4364 | mixed | ND | 58.61372 | 23.81123 | 2015-08-21 |
| G4362 | mixed | ND | 58.55742 | 23.55254 | 2015-08-21 |
| G4363 | mixed | ND | 58.65203 | 23.62096 | 2015-08-21 |
| G4361 | Alnus_incana | native | 58.58855 | 23.57538 | 2015-08-21 |
| S510 | mixed | ND | 58.1282 | 23.98151 | 2015-08-22 |
| S511 | mixed | ND | 58.14078 | 23.99018 | 2015-08-22 |
| S512 | mixed | ND | 58.13262 | 23.97074 | 2015-08-22 |
| S482 | mixed | ND | 59.3267 | 24.5699 | 2015-08-22 |
| S487 | mixed | ND | 58.18964 | 26.20608 | 2015-08-23 |
| S488 | mixed | ND | 58.22851 | 26.2963 | 2015-08-23 |
| S483 | mixed | ND | 58.1843 | 27.3847 | 2015-08-23 |
| S484 | Alnus_glutinosa | native | 58.217 | 27.4399 | 2015-08-23 |
| S485 | mixed | ND | 58.2017 | 26.5791 | 2015-08-26 |
| G4368 | mixed | ND | 59.51025 | 26.57118 | 2015-08-27 |
| G4365 | mixed | ND | 59.48589 | 26.52472 | 2015-08-27 |
| G4367 | Tilia_cordata | native | 59.49747 | 26.52084 | 2015-08-27 |
| G4366 | Quercus_robur | native | 59.50728 | 26.54138 | 2015-08-27 |
| G4372 | mixed | ND | 58.69554 | 26.91282 | 2015-09-04 |
| G4374 | Tilia_cordata | native | 58.60436 | 27.12826 | 2015-09-04 |
| S490 | mixed | ND | 58.23853 | 26.21607 | 2015-09-06 |

| | | | | | |
|---|---|---|---|---|---|
| S556 | mixed | ND | 59.19716 | 26.15651 | 2015-09-06 |
| S559 | mixed | ND | 59.13592 | 26.16336 | 2015-09-06 |
| S489 | mixed | ND | 58.21269 | 26.26296 | 2015-09-06 |
| G4375 | mixed | ND | 58.28933 | 26.94715 | 2015-09-06 |
| S561 | mixed | ND | 59.16674 | 26.07309 | 2015-09-06 |
| G4376 | mixed | ND | 58.03576 | 26.75627 | 2015-09-10 |
| G4378 | mixed | ND | 58.00251 | 26.67547 | 2015-09-10 |
| G4377 | mixed | ND | 58.01186 | 26.66654 | 2015-09-10 |
| G4380 | mixed | ND | 57.52467 | 26.48508 | 2015-09-11 |
| S577 | mixed | ND | 58.03085 | 26.39882 | 2015-09-11 |
| G4379 | mixed | ND | 57.54291 | 26.45242 | 2015-09-11 |
| G4383 | mixed | ND | 57.90104 | 26.28249 | 2015-09-12 |
| G4384 | mixed | ND | 57.93279 | 26.23701 | 2015-09-12 |
| S595 | mixed | ND | 58.03424 | 26.47601 | 2015-09-12 |
| G4381 | mixed | ND | 57.88603 | 26.38902 | 2015-09-12 |
| G4382 | mixed | ND | 57.90261 | 26.27616 | 2015-09-12 |
| G4385 | mixed | ND | 57.95575 | 26.27273 | 2015-09-12 |
| G4386 | mixed | ND | 57.70143 | 26.45922 | 2015-09-13 |
| G4389 | mixed | ND | 57.52091 | 26.6012 | 2015-09-13 |
| S550 | mixed | ND | 57.5512 | 26.55757 | 2015-09-13 |
| S594 | mixed | ND | 57.55567 | 26.54335 | 2015-09-13 |
| G4388 | mixed | ND | 57.51062 | 26.61442 | 2015-09-13 |
| G4393 | mixed | ND | 58.04115 | 26.36303 | 2015-09-14 |
| S579 | mixed | ND | 57.57234 | 26.667 | 2015-09-14 |
| S578 | mixed | ND | 57.58198 | 26.64923 | 2015-09-14 |
| G4390 | mixed | ND | 58.01453 | 26.46222 | 2015-09-14 |
| S576 | mixed | ND | 57.53688 | 26.58616 | 2015-09-14 |
| G4401 | mixed | ND | 57.91884 | 26.63531 | 2015-09-15 |
| G4394 | mixed | ND | 57.98353 | 26.3588 | 2015-09-15 |
| G4402 | mixed | ND | 57.97119 | 26.59306 | 2015-09-15 |
| G4395 | mixed | ND | 57.96374 | 26.34832 | 2015-09-15 |
| G4396 | mixed | ND | 57.94794 | 26.38082 | 2015-09-15 |
| G4403 | mixed | ND | 57.92431 | 26.7624 | 2015-09-16 |
| S585 | mixed | ND | 59.14031 | 24.23269 | 2015-09-17 |
| S584 | mixed | ND | 59.18165 | 24.56432 | 2015-09-17 |
| G4406 | mixed | ND | 59.20745 | 27.15334 | 2015-09-18 |
| S492 | mixed | ND | 59.23487 | 27.38942 | 2015-09-18 |
| G4405 | mixed | ND | 59.22482 | 27.34011 | 2015-09-18 |
| G4407 | mixed | ND | 59.1842 | 27.15056 | 2015-09-18 |
| S491 | mixed | ND | 59.37022 | 27.49997 | 2015-09-18 |
| S587 | mixed | ND | 59.02434 | 24.37966 | 2015-09-18 |
| S588 | mixed | ND | 59.08235 | 24.31246 | 2015-09-18 |
| G4404 | mixed | ND | 59.39022 | 27.45956 | 2015-09-18 |
| S586 | mixed | ND | 59.02274 | 24.40731 | 2015-09-18 |
| S589 | mixed | ND | 59.10435 | 24.34035 | 2015-09-19 |
| G4412 | mixed | ND | 57.97854 | 25.55026 | 2015-09-20 |

| | | | | | |
|---|---|---|---|---|---|
| S554 | mixed | ND | 59.32169 | 26.2621 | 2015-09-20 |
| G4413 | mixed | ND | 57.98693 | 25.56089 | 2015-09-20 |
| S553 | mixed | ND | 59.30415 | 26.24502 | 2015-09-20 |
| S591 | mixed | ND | 59.04086 | 24.507 | 2015-09-20 |
| S590 | mixed | ND | 59.01857 | 24.28041 | 2015-09-20 |
| G4414 | mixed | ND | 57.99961 | 25.54629 | 2015-09-20 |
| S499 | mixed | ND | 58.4176 | 26.588 | 2015-09-22 |
| S495 | Picea_abies | native | 58.3701 | 26.5561 | 2015-09-22 |
| G4420 | mixed | ND | 58.67367 | 24.61965 | 2015-09-24 |
| S493 | mixed | ND | 58.67577 | 24.60297 | 2015-09-24 |
| G4419 | mixed | ND | 58.67427 | 24.61056 | 2015-09-24 |
| G4418 | mixed | ND | 58.68433 | 24.69581 | 2015-09-24 |
| G4417 | mixed | ND | 58.67886 | 24.69484 | 2015-09-24 |
| S574 | mixed | ND | 58.46721 | 22.3082 | 2015-09-25 |
| G4424 | mixed | ND | 58.28111 | 21.83647 | 2015-09-25 |
| G4425 | mixed | ND | 58.17355 | 22.14902 | 2015-09-25 |
| G4423 | mixed | ND | 58.29241 | 21.85424 | 2015-09-25 |
| S569 | mixed | ND | 58.3782 | 22.21022 | 2015-09-25 |
| G4426 | mixed | ND | 58.34617 | 21.96826 | 2015-09-25 |
| S572 | mixed | ND | 58.49144 | 22.21582 | 2015-09-25 |
| S571 | mixed | ND | 58.37142 | 22.20443 | 2015-09-25 |
| S573 | mixed | ND | 58.43581 | 22.04076 | 2015-09-25 |
| G4422 | Corylus_avellana | native | 58.30035 | 21.83533 | 2015-09-25 |
| S544 | mixed | ND | 59.4228 | 26.1435 | 2015-09-26 |
| S543 | mixed | ND | 59.4561 | 26.0051 | 2015-09-26 |
| S558 | mixed | ND | 59.3382 | 26.3507 | 2015-09-26 |
| S545 | mixed | ND | 59.5711 | 26.2712 | 2015-09-26 |
| S546 | mixed | ND | 59.4965 | 26.3097 | 2015-09-26 |
| S547 | mixed | ND | 59.3901 | 26.3754 | 2015-09-26 |
| G4427 | mixed | ND | 58.11562 | 22.21424 | 2015-09-26 |
| G4428 | mixed | ND | 58.11566 | 22.21431 | 2015-09-26 |
| G4430 | mixed | ND | 57.95327 | 22.12184 | 2015-09-26 |
| G4431 | mixed | ND | 58.58195 | 22.54854 | 2015-09-27 |
| G4436 | mixed | ND | 58.42778 | 24.28947 | 2015-09-27 |
| G4434 | mixed | ND | 58.5377 | 24.06659 | 2015-09-27 |
| G4432 | mixed | ND | 58.47507 | 22.94365 | 2015-09-27 |
| G4435 | mixed | ND | 58.47546 | 24.3331 | 2015-09-27 |
| S570 | Betula_pendula | native | 58.47962 | 22.94584 | 2015-09-27 |
| G4448 | mixed | ND | 58.62274 | 26.2926 | 2015-09-30 |
| G4444 | mixed | ND | 58.53676 | 26.28322 | 2015-09-30 |
| G4445 | mixed | ND | 58.54556 | 26.37929 | 2015-09-30 |
| G4449 | mixed | ND | 58.70322 | 26.14475 | 2015-09-30 |
| G4446 | mixed | ND | 58.58016 | 26.43521 | 2015-09-30 |
| G4454 | mixed | ND | 58.06163 | 24.54246 | 2015-10-04 |
| G4450 | mixed | ND | 58.019 | 24.68039 | 2015-10-04 |
| G4452 | mixed | ND | 57.98103 | 24.67273 | 2015-10-04 |

| | | | | | |
|---|---|---|---|---|---|
| G4451 | mixed | ND | 57.94707 | 24.62104 | 2015-10-04 |
| S583 | mixed | ND | 58.33326 | 27.18975 | 2015-10-04 |
| G4455 | mixed | ND | 57.53212 | 27.33267 | 2015-10-06 |
| G4456 | mixed | ND | 57.43844 | 27.30075 | 2015-10-06 |
| G4457 | mixed | ND | 57.50349 | 27.10151 | 2015-10-06 |
| G4458 | Quercus_robur | native | 57.62578 | 26.9993 | 2015-10-06 |
| G4459 | Picea_abies | native | 57.62597 | 26.95557 | 2015-10-06 |
| G4461 | Salix_viminalis | native | 58.28634 | 26.54899 | 2015-10-08 |
| G4462 | Salix_viminalis | native | 58.25166 | 26.71276 | 2015-10-08 |
| G4467 | mixed | ND | 59.38033 | 25.3035 | 2015-10-09 |
| G4468 | mixed | ND | 59.16326 | 25.42893 | 2015-10-09 |
| G4463 | mixed | ND | 59.42742 | 25.01653 | 2015-10-09 |
| G4471 | mixed | ND | 59.20556 | 25.10089 | 2015-10-09 |
| G4464 | mixed | ND | 59.45059 | 25.22167 | 2015-10-09 |
| S560 | mixed | ND | 58.9926 | 26.3414 | 2015-10-09 |
| G4470 | mixed | ND | 59.18189 | 25.22027 | 2015-10-09 |
| G4469 | Salix_caprea | native | 59.17969 | 25.23308 | 2015-10-09 |
| G4465 | Salix_alba | introduced | 59.44521 | 25.26929 | 2015-10-09 |
| G4476 | mixed | ND | 59.35663 | 24.90358 | 2015-10-10 |
| G4473 | mixed | ND | 59.34574 | 24.87833 | 2015-10-10 |
| G4474 | mixed | ND | 59.35123 | 24.95902 | 2015-10-10 |
| G4475 | mixed | ND | 59.32602 | 25.11184 | 2015-10-10 |
| S557 | mixed | ND | 59.0984 | 26.5345 | 2015-10-11 |
| S551 | Quercus_robur | native | 59.2819 | 26.2523 | 2015-10-11 |
| G4492 | mixed | ND | 57.90369 | 26.2774 | 2015-10-13 |
| G4477 | mixed | ND | 57.90066 | 26.28343 | 2015-10-13 |
| G4498 | mixed | ND | 59.15358 | 25.65769 | 2015-10-14 |
| G4500 | mixed | ND | 59.12606 | 25.85231 | 2015-10-14 |
| G4501 | mixed | ND | 59.12461 | 25.85034 | 2015-10-14 |
| G4502 | mixed | ND | 59.11289 | 25.80023 | 2015-10-14 |
| G4495 | Tilia_cordata | native | 59.02957 | 25.70598 | 2015-10-14 |
| G4496 | Tilia_cordata | native | 59.09768 | 25.69973 | 2015-10-14 |
| G4497 | Salix_caprea | native | 59.15164 | 25.66416 | 2015-10-14 |
| G4499 | Picea_abies | native | 59.12809 | 25.76396 | 2015-10-14 |
| S562 | mixed | ND | 59.1243 | 26.31548 | 2015-10-17 |
| S565 | mixed | ND | 59.29079 | 26.43204 | 2015-10-17 |
| S549 | mixed | ND | 59.27565 | 26.3543 | 2015-10-17 |
| G4504 | mixed | ND | 58.23795 | 27.2178 | 2015-10-17 |
| G4503 | mixed | ND | 58.27394 | 26.92525 | 2015-10-17 |
| G4505 | mixed | ND | 58.27247 | 27.06854 | 2015-10-17 |
| G4506 | mixed | ND | 58.29724 | 27.06813 | 2015-10-17 |
| S548 | mixed | ND | 59.04003 | 26.74493 | 2015-10-18 |
| S552 | mixed | ND | 58.9652 | 26.6024 | 2015-10-18 |
| G4508 | mixed | ND | 58.41209 | 26.96311 | 2015-10-18 |
| G4507 | Salix_caprea | native | 58.38237 | 26.96401 | 2015-10-18 |
| G4514 | mixed | ND | 58.69157 | 25.69677 | 2015-10-20 |

| | | | | | |
|---|---|---|---|---|---|
| G4510 | mixed | ND | 58.65885 | 25.25881 | 2015-10-20 |
| G4513 | Populus_tremula | native | 58.67847 | 25.5136 | 2015-10-20 |
| G4515 | mixed | ND | 58.40486 | 25.85168 | 2015-10-22 |
| G4516 | mixed | ND | 58.45857 | 25.83877 | 2015-10-22 |
| G4518 | mixed | ND | 58.35005 | 25.60564 | 2015-10-22 |
| G4519 | mixed | ND | 58.34921 | 25.53711 | 2015-10-22 |
| G4520 | mixed | ND | 58.38441 | 25.33371 | 2015-10-22 |
| G4517 | Betula_pendula | native | 58.45543 | 25.83043 | 2015-10-22 |
| G4521 | Salix_alba | introduced | 58.36391 | 25.58831 | 2015-10-22 |
| G4527 | mixed | ND | 59.28319 | 26.77791 | 2015-10-24 |
| G4522 | mixed | ND | 59.00103 | 26.79703 | 2015-10-24 |
| G4526 | mixed | ND | 59.24696 | 26.528 | 2015-10-24 |
| G4524 | mixed | ND | 59.17149 | 26.5875 | 2015-10-24 |
| G4528 | mixed | ND | 59.29111 | 26.88034 | 2015-10-24 |
| G4523 | Tilia_cordata | native | 59.11146 | 26.63559 | 2015-10-24 |
| G4529 | mixed | ND | 58.17839 | 26.86382 | 2015-10-25 |
| G4531 | mixed | ND | 57.62166 | 27.1793 | 2015-11-03 |
| G4532 | mixed | ND | 57.63871 | 27.08991 | 2015-11-03 |
| G4538 | mixed | ND | 58.95459 | 26.37764 | 2015-11-05 |
| G4537 | mixed | ND | 59.00988 | 26.11501 | 2015-11-05 |
| G4534 | mixed | ND | 58.94356 | 26.08618 | 2015-11-05 |
| G4536 | mixed | ND | 58.97005 | 25.94334 | 2015-11-05 |
| G4535 | Salix_alba | introduced | 58.9361 | 26.0736 | 2015-11-05 |
| S580 | mixed | ND | 58.28311 | 24.72518 | 2015-11-15 |
| G4540 | mixed | ND | 57.73179 | 26.92081 | 2015-11-15 |
| S581 | mixed | ND | 58.31284 | 24.68531 | 2015-11-15 |
| S582 | mixed | ND | 58.33673 | 24.65504 | 2015-11-16 |
| G4543 | mixed | ND | 59.31302 | 24.49305 | 2015-11-21 |
| G4544 | mixed | ND | 59.3875 | 24.4164 | 2015-11-21 |
| G4542 | mixed | ND | 57.85263 | 26.87997 | 2015-11-21 |
| G4545 | mixed | ND | 59.11129 | 24.77781 | 2015-11-22 |
| G4546 | mixed | ND | 59.06406 | 24.67274 | 2015-11-22 |
| G4547 | mixed | ND | 59.06671 | 24.70329 | 2015-11-22 |
| S593 | Quercus_robur | native | 58.46851 | 26.72559 | 2015-12-05 |
| G4553 | mixed | ND | 57.85546 | 24.53828 | 2015-12-06 |
| G4550 | mixed | ND | 58.04162 | 25.01002 | 2015-12-06 |
| G4551 | mixed | ND | 58.03189 | 24.96371 | 2015-12-06 |
| G4552 | mixed | ND | 57.84563 | 24.71121 | 2015-12-06 |
| G4554 | mixed | ND | 58.35735 | 26.88689 | 2015-12-13 |
| G4556 | mixed | ND | 58.65833 | 25.96883 | 2015-12-19 |
| G4555 | mixed | ND | 58.55958 | 25.84749 | 2015-12-19 |
| G4557 | Picea_abies | native | 59.54978 | 24.80877 | 2015-12-25 |
| G4559 | mixed | ND | 59.44182 | 24.50343 | 2015-12-26 |
| G4558 | mixed | ND | 59.44064 | 24.52266 | 2015-12-26 |
| G4562 | mixed | ND | 59.40053 | 24.25788 | 2015-12-26 |
| G4561 | Corylus_avellana | native | 59.42105 | 24.33486 | 2015-12-26 |

| | | | | | |
|---|---|---|---|---|---|
| G4563 | Betula_pendula | native | 59.4085 | 24.7985 | 2016-02-13 |
| G4564 | mixed | ND | 59.43556 | 24.63726 | 2016-02-14 |
| G4597 | mixed | ND | 57.9528 | 27.12351 | 2016-03-20 |
| G4596 | Quercus_robur | native | 57.83847 | 27.19867 | 2016-03-20 |
| G4599 | mixed | ND | 58.07906 | 26.00937 | 2016-03-25 |
| G4600 | mixed | ND | 58.17785 | 25.65604 | 2016-03-25 |
| G4603 | mixed | ND | 58.24544 | 26.68631 | 2016-04-03 |
| G4602 | mixed | ND | 58.02791 | 26.92454 | 2016-04-03 |
| G4606 | mixed | ND | 59.32067 | 25.43214 | 2016-04-07 |
| G4604 | mixed | ND | 59.24726 | 25.68298 | 2016-04-07 |
| G4605 | mixed | ND | 59.25269 | 25.64206 | 2016-04-07 |
| G4607 | mixed | ND | 59.32068 | 25.43215 | 2016-04-07 |
| G4609 | mixed | ND | 59.20578 | 24.92858 | 2016-04-09 |
| G4608 | Quercus_robur | native | 59.22307 | 24.97138 | 2016-04-09 |
| G4610 | Salix_caprea | native | 59.29395 | 24.90647 | 2016-04-09 |
| G4612 | mixed | ND | 58.94682 | 26.37125 | 2016-04-16 |
| G4613 | Betula_pendula | native | 58.74765 | 26.62599 | 2016-04-16 |
| G4611 | Alnus_incana | native | 58.71484 | 26.43655 | 2016-04-16 |
| G4615 | mixed | ND | 58.21683 | 26.92623 | 2016-05-01 |
| G4614 | Salix_alba | introduced | 58.21787 | 26.93233 | 2016-05-01 |
| G4616 | mixed | ND | 59.35593 | 24.14681 | 2016-05-06 |
| G4619 | mixed | ND | 59.0828 | 24.27337 | 2016-05-06 |
| G4618 | mixed | ND | 59.07597 | 24.13359 | 2016-05-06 |
| G4617 | Salix_caprea | native | 59.35358 | 24.14266 | 2016-05-06 |
| G4622 | mixed | ND | 59.21186 | 23.93378 | 2016-05-07 |
| G4620 | mixed | ND | 59.21874 | 24.14135 | 2016-05-07 |
| G4621 | mixed | ND | 59.18242 | 24.26531 | 2016-05-07 |
| G4625 | mixed | ND | 58.73808 | 25.80413 | 2016-05-08 |
| G4624 | mixed | ND | 58.76628 | 25.78772 | 2016-05-08 |
| G4623 | Pinus_sylvestris | native | 59.18587 | 23.83031 | 2016-05-08 |
| G4628 | mixed | ND | 59.1545 | 26.8274 | 2016-05-11 |
| G4626 | mixed | ND | 59.07754 | 26.76145 | 2016-05-11 |
| G4627 | mixed | ND | 59.11369 | 26.75944 | 2016-05-11 |
| G4629 | mixed | ND | 58.50582 | 26.48343 | 2016-05-15 |
| G4630 | mixed | ND | 58.52922 | 26.35785 | 2016-05-15 |
| G4631 | Salix_alba | introduced | 58.38029 | 26.73367 | 2016-05-15 |
| G4633 | mixed | ND | 57.71522 | 27.0577 | 2016-05-17 |
| G4634 | mixed | ND | 57.54299 | 26.9449 | 2016-05-17 |
| G4632 | mixed | ND | 57.73489 | 27.06409 | 2016-05-17 |
| G4636 | mixed | ND | 57.72659 | 26.92051 | 2016-05-17 |
| G4635 | Corylus_avellana | native | 57.52906 | 26.86514 | 2016-05-17 |
| G4637 | Tilia_cordata | native | 58.25126 | 26.78071 | 2016-05-20 |
| G4638 | mixed | ND | 58.39058 | 26.88647 | 2016-05-22 |
| G4643 | mixed | ND | 58.46726 | 25.44357 | 2016-06-05 |
| G4644 | mixed | ND | 58.39563 | 25.60391 | 2016-06-05 |
| G4639 | Tilia_cordata | native | 58.35937 | 25.59228 | 2016-06-05 |

| | | | | | |
|---|---|---|---|---|---|
| G4641 | Betula_pendula | native | 58.36562 | 26.62687 | 2016-06-05 |
| G4642 | Quercus_robur | native | 58.36566 | 25.58586 | 2016-06-05 |
| G4640 | Corylus_avellana | native | 58.36003 | 25.59288 | 2016-06-05 |
| G4645 | mixed | ND | 59.215 | 25.042 | 2016-06-20 |
| G4649 | mixed | ND | 59.5836 | 25.6283 | 2016-06-21 |
| G4646 | mixed | ND | 59.4044 | 25.9027 | 2016-06-21 |
| G4647 | mixed | ND | 59.4798 | 25.9058 | 2016-06-21 |
| G4650 | Picea_abies | native | 59.5836 | 25.6273 | 2016-06-21 |
| G4648 | Pinus_sylvestris | native | 59.5769 | 25.9528 | 2016-06-21 |
| G4652 | mixed | ND | 59.4423 | 24.526 | 2016-06-22 |
| G4651 | Betula_pendula | native | 59.4194 | 24.66265 | 2016-06-22 |
| G4653 | Salix_alba | introduced | 59.4433 | 24.7938 | 2016-06-23 |
| G4669 | mixed | ND | 59.02551 | 24.95239 | 2016-06-24 |
| G4666 | Betula_pendula | native | 59.0671 | 24.7593 | 2016-06-24 |
| G4667 | Salix_caprea | native | 59.06159 | 24.75966 | 2016-06-24 |
| G4670 | mixed | ND | 59.366 | 24.781 | 2016-06-25 |
| G4672 | mixed | ND | 59.47026 | 24.88203 | 2016-06-26 |
| G4671 | Tilia_cordata | native | 59.46705 | 24.87833 | 2016-06-26 |
| G4673 | mixed | ND | 58.022 | 26.9251 | 2016-06-27 |
| S674 | mixed | ND | 58.62933 | 23.02356 | 2016-07-01 |
| S675 | mixed | ND | 58.00971 | 22.12827 | 2016-07-02 |
| G4675 | mixed | ND | 58.24519 | 22.0354 | 2016-07-02 |
| S678 | mixed | ND | 58.28355 | 22.54061 | 2016-07-03 |
| S676 | mixed | ND | 58.31299 | 22.41582 | 2016-07-03 |
| S677 | mixed | ND | 58.2844 | 22.54025 | 2016-07-03 |
| S679 | mixed | ND | 58.40062 | 22.25178 | 2016-07-03 |
| S680 | mixed | ND | 58.23121 | 22.08396 | 2016-07-04 |
| G4678 | mixed | ND | 58.25124 | 22.00562 | 2016-07-04 |
| S681 | mixed | ND | 58.26628 | 22.12264 | 2016-07-04 |
| S682 | mixed | ND | 58.48718 | 21.95993 | 2016-07-04 |
| G4676 | Corylus_avellana | native | 58.263 | 22.0343 | 2016-07-04 |
| G4677 | Salix_cinerea | native | 58.2617 | 21.9127 | 2016-07-04 |
| S683 | mixed | ND | 58.44261 | 22.29135 | 2016-07-05 |
| G4683 | mixed | ND | 59.41085 | 24.79904 | 2016-07-05 |
| S685 | mixed | ND | 58.34873 | 22.07884 | 2016-07-05 |
| S684 | Tilia_cordata | native | 58.46634 | 22.30828 | 2016-07-05 |
| S686 | mixed | ND | 58.15469 | 22.50151 | 2016-07-06 |
| G4682 | mixed | ND | 59.6342 | 24.99949 | 2016-07-06 |
| G4681 | Populus_tremula | native | 59.61553 | 24.99532 | 2016-07-06 |
| G4680 | Salix_phylicifolia | native | 59.61535 | 24.98596 | 2016-07-06 |
| G4679 | Salix_triandra | native | 59.61505 | 24.98713 | 2016-07-06 |
| G4686 | mixed | ND | 57.79313 | 27.1696 | 2016-07-16 |
| G4687 | mixed | ND | 57.799 | 27.1559 | 2016-07-16 |
| G4685 | mixed | ND | 57.8089 | 27.04915 | 2016-07-16 |
| G4688 | Salix_alba | introduced | 58.37058 | 26.79971 | 2016-07-17 |
| G4689 | mixed | ND | 58.32371 | 25.90441 | 2016-07-19 |

| | | | | | |
|---|---|---|---|---|---|
| G4693 | mixed | ND | 58.28012 | 25.58443 | 2016-07-19 |
| G4690 | mixed | ND | 58.30279 | 25.81747 | 2016-07-19 |
| G4694 | mixed | ND | 58.33807 | 25.45529 | 2016-07-19 |
| G4691 | Tilia_cordata | native | 58.2344 | 25.8958 | 2016-07-19 |
| G4692 | Salix_pentandra | native | 58.24891 | 25.68695 | 2016-07-19 |
| G4700 | mixed | ND | 58.976 | 26.048 | 2016-07-21 |
| G4699 | mixed | ND | 58.981 | 26.2587 | 2016-07-21 |
| G4696 | mixed | ND | 58.73948 | 26.64526 | 2016-07-21 |
| G4698 | mixed | ND | 58.988 | 26.32 | 2016-07-21 |
| G4697 | Quercus_robur | native | 58.76801 | 26.5651 | 2016-07-21 |
| G4695 | Salix_caprea | native | 58.63503 | 26.69661 | 2016-07-21 |
| G4701 | Salix_caprea | native | 58.35759 | 26.63616 | 2016-07-23 |
| G4702 | mixed | ND | 57.71752 | 27.05494 | 2016-07-24 |
| S702 | mixed | ND | 57.80113 | 23.26129 | 2016-07-30 |
| S673 | mixed | ND | 58.1447 | 23.9683 | 2016-07-30 |
| S703 | Corylus_avellana | native | 57.80539 | 23.24117 | 2016-07-30 |
| S672 | Pinus_sylvestris | native | 58.1448 | 24.0118 | 2016-07-30 |
| S701 | Pinus_sylvestris | native | 57.78934 | 23.27037 | 2016-07-30 |
| G4704 | mixed | ND | 59.21817 | 27.35086 | 2016-08-01 |
| G4705 | mixed | ND | 59.2551 | 27.5777 | 2016-08-01 |
| G4703 | mixed | ND | 59.224 | 27.326 | 2016-08-01 |
| G4706 | Tilia_cordata | native | 59.27139 | 27.51445 | 2016-08-01 |
| G4723 | mixed | ND | 58.62853 | 23.43437 | 2016-08-15 |
| G4724 | Populus_berolinensis | introduced | 58.7297 | 23.9714 | 2016-08-15 |
| G4725 | Salix_alba | introduced | 58.55894 | 23.55104 | 2016-08-15 |
| G4728 | mixed | ND | 59.00616 | 23.1999 | 2016-08-17 |
| G4727 | mixed | ND | 58.9914 | 23.193 | 2016-08-17 |
| G4726 | mixed | ND | 58.99897 | 23.25202 | 2016-08-17 |
| G4729 | Picea_abies | native | 59.02163 | 23.20193 | 2016-08-17 |
| G4732 | mixed | ND | 58.73506 | 23.675 | 2016-08-18 |
| G4730 | Corylus_avellana | native | 58.66996 | 23.69916 | 2016-08-18 |
| G4733 | mixed | ND | 58.23877 | 24.61423 | 2016-08-19 |
| G4735 | mixed | ND | 58.02361 | 24.64315 | 2016-08-19 |
| G4734 | mixed | ND | 58.02674 | 24.65739 | 2016-08-19 |
| G4738 | mixed | ND | 58.20056 | 26.30843 | 2016-08-26 |
| G4736 | mixed | ND | 58.15944 | 26.23042 | 2016-08-26 |
| G4737 | mixed | ND | 58.15135 | 26.24649 | 2016-08-26 |
| G4739 | Salix_pentandra | native | 58.13964 | 26.3585 | 2016-08-26 |
| G4742 | mixed | ND | 58.14442 | 25.26283 | 2016-08-29 |
| G4743 | mixed | ND | 58.1307 | 25.26657 | 2016-08-29 |
| G4741 | mixed | ND | 58.04791 | 25.38875 | 2016-08-29 |
| G4740 | mixed | ND | 58.04893 | 25.39788 | 2016-08-29 |
| G4747 | mixed | ND | 57.5942 | 26.3241 | 2016-08-31 |
| G4744 | mixed | ND | 57.75734 | 26.19106 | 2016-08-31 |
| G4745 | mixed | ND | 57.75198 | 26.21503 | 2016-08-31 |
| G4746 | Betula_pendula | native | 57.59761 | 26.35493 | 2016-08-31 |

| | | | | | |
|---|---|---|---|---|---|
| G4748 | Quercus_robur | native | 57.59131 | 26.3195 | 2016-08-31 |
| G4750 | mixed | ND | 58.34605 | 26.66094 | 2016-09-03 |
| G4749 | Picea_abies | native | 58.30085 | 26.58832 | 2016-09-03 |
| G4751 | mixed | ND | 59.30702 | 26.45794 | 2016-09-09 |
| G4752 | mixed | ND | 59.32449 | 26.58481 | 2016-09-09 |
| G4753 | mixed | ND | 59.30541 | 26.13707 | 2016-09-09 |
| G4754 | mixed | ND | 59.25681 | 26.22693 | 2016-09-09 |
| G4756 | mixed | ND | 59.05143 | 26.22834 | 2016-09-09 |
| G4755 | Quercus_robur | native | 59.28117 | 26.24996 | 2016-09-09 |
| G4757 | Picea_abies | native | 58.23762 | 26.6844 | 2016-09-10 |
| G4759 | mixed | ND | 58.37569 | 26.5569 | 2016-09-11 |
| G4760 | mixed | ND | 58.52618 | 26.7138 | 2016-09-11 |
| DD1 | mixed | ND | 58.40344 | 26.65631 | 2016-09-15 |
| FF1 | mixed | ND | 58.40476 | 26.63817 | 2016-09-16 |
| G4761 | mixed | ND | 58.27948 | 25.58374 | 2016-09-16 |
| G4767 | mixed | ND | 58.35996 | 25.75669 | 2016-09-16 |
| G4766 | mixed | ND | 58.3363 | 25.78084 | 2016-09-16 |
| G4763 | mixed | ND | 58.17612 | 25.68227 | 2016-09-16 |
| G4764 | mixed | ND | 58.18281 | 25.73653 | 2016-09-16 |
| G4765 | Salix_pentandra | native | 58.29475 | 25.88172 | 2016-09-16 |
| G4762 | Picea_abies | native | 58.27976 | 25.58477 | 2016-09-16 |
| G4768 | mixed | ND | 58.97949 | 25.59474 | 2016-09-18 |
| CC1 | mixed | ND | 58.27671 | 26.68848 | 2016-09-19 |
| DD2 | mixed | ND | 58.30115 | 26.67519 | 2016-09-20 |
| S719 | mixed | ND | 58.4176 | 26.588 | 2016-09-21 |
| G4772 | mixed | ND | 58.95935 | 24.30018 | 2016-09-22 |
| G4769 | mixed | ND | 59.27369 | 24.55078 | 2016-09-22 |
| G4770 | Populus_berolinensis | introduced | 59.06755 | 24.47843 | 2016-09-22 |
| G4771 | Salix_pentandra | native | 59.00725 | 24.5702 | 2016-09-22 |
| G4774 | Salix_caprea | native | 59.22869 | 24.13686 | 2016-09-22 |
| G4773 | Salix_alba | introduced | 59.27627 | 24.45874 | 2016-09-22 |
| CC2 | mixed | ND | 58.41618 | 26.61541 | 2016-09-23 |
| G4775 | mixed | ND | 59.31387 | 23.93453 | 2016-09-23 |
| G4776 | Salix_caprea | native | 59.34427 | 23.91938 | 2016-09-23 |
| G4781 | mixed | ND | 58.91729 | 23.55022 | 2016-09-24 |
| G4782 | mixed | ND | 58.77606 | 23.48857 | 2016-09-24 |
| G4783 | mixed | ND | 58.78136 | 23.55082 | 2016-09-24 |
| G4780 | mixed | ND | 58.92795 | 23.48759 | 2016-09-24 |
| G4784 | Betula_pendula | native | 58.78795 | 23.53275 | 2016-09-24 |
| G4785 | mixed | ND | 58.99756 | 23.73995 | 2016-09-24 |
| G4790 | mixed | ND | 59.47063 | 25.2983 | 2016-09-25 |
| G4786 | mixed | ND | 59.22915 | 24.01194 | 2016-09-25 |
| G4787 | mixed | ND | 59.39427 | 24.29545 | 2016-09-25 |
| G4788 | Tilia_cordata | native | 59.43496 | 24.54918 | 2016-09-25 |
| EE1 | mixed | ND | 58.54457 | 26.88323 | 2016-09-26 |
| G4792 | mixed | ND | 57.83887 | 26.40866 | 2016-09-29 |

| | | | | | |
|---|---|---|---|---|---|
| G4794 | mixed | ND | 57.91782 | 26.52069 | 2016-09-29 |
| G4791 | mixed | ND | 57.90808 | 26.27391 | 2016-09-29 |
| G4793 | Quercus_robur | native | 57.8622 | 26.51757 | 2016-09-29 |
| EE2 | mixed | ND | 58.41251 | 26.6349 | 2016-10-03 |
| DD3 | mixed | ND | 58.23444 | 26.67666 | 2016-10-05 |
| G4795 | mixed | ND | 58.74809 | 26.49781 | 2016-10-07 |
| G4796 | mixed | ND | 58.7562 | 26.51096 | 2016-10-07 |
| G4797 | mixed | ND | 58.54841 | 26.92724 | 2016-10-07 |
| EE3 | mixed | ND | 58.43037 | 26.54341 | 2016-10-11 |
| FF2 | mixed | ND | 58.405 | 26.64117 | 2016-10-12 |
| G4798 | mixed | ND | 58.44381 | 25.50775 | 2016-10-12 |
| G4800 | mixed | ND | 58.41593 | 25.23269 | 2016-10-12 |
| G4801 | mixed | ND | 58.48005 | 24.98509 | 2016-10-12 |
| G4802 | mixed | ND | 58.45035 | 25.05619 | 2016-10-12 |
| G4799 | Pinus_sylvestris | native | 58.41171 | 25.28874 | 2016-10-12 |
| G4803 | mixed | ND | 58.05628 | 26.33548 | 2016-10-14 |
| G4804 | mixed | ND | 58.0214 | 26.06243 | 2016-10-14 |
| G4807 | mixed | ND | 58.11438 | 26.17691 | 2016-10-14 |
| G4805 | Salix_caprea | native | 58.14228 | 25.92182 | 2016-10-14 |
| G4811 | mixed | ND | 58.30383 | 25.44702 | 2016-10-21 |
| G4812 | mixed | ND | 58.33115 | 25.51401 | 2016-10-21 |
| G4809 | mixed | ND | 58.3609 | 25.41844 | 2016-10-21 |
| G4810 | mixed | ND | 58.27159 | 25.18275 | 2016-10-21 |
| D2 | mixed | ND | 58.2716 | 26.64082 | 2016-10-27 |
| D1 | mixed | ND | 58.27064 | 26.64392 | 2016-10-27 |
| BB1 | Picea_abies | native | 58.28809 | 26.63314 | 2016-10-27 |
| G4813 | mixed | ND | 57.6942 | 27.37766 | 2016-10-29 |
| C5 | mixed | ND | 58.33796 | 27.04647 | 2016-11-11 |
| E2 | mixed | ND | 58.54332 | 26.86359 | 2016-11-17 |
| E1 | mixed | ND | 58.54058 | 26.86708 | 2016-11-17 |
| B6 | Picea_abies | native | 58.19571 | 26.66877 | 2016-11-23 |
| C1 | mixed | ND | 58.1886 | 26.79476 | 2016-11-24 |
| C3 | mixed | ND | 58.20302 | 26.73655 | 2016-11-24 |
| B5 | Picea_abies | native | 58.18892 | 26.80318 | 2016-11-24 |
| A5 | mixed | ND | 58.52349 | 26.96373 | 2016-12-01 |
| C2 | mixed | ND | 58.52217 | 26.96401 | 2016-12-01 |
| D16 | mixed | ND | 58.52061 | 26.95905 | 2016-12-01 |
| E3 | mixed | ND | 58.59431 | 27.13954 | 2016-12-29 |
| D4 | mixed | ND | 58.59426 | 27.14257 | 2016-12-29 |
| D3 | mixed | ND | 58.16489 | 27.04083 | 2017-01-16 |
| A29 | mixed | ND | 58.74054 | 26.65621 | 2017-01-27 |
| D7 | mixed | ND | 59.23766 | 25.66597 | 2017-03-09 |
| F1 | mixed | ND | 58.53249 | 26.72314 | 2017-03-24 |
| C4 | mixed | ND | 58.54311 | 27.16238 | 2017-03-24 |
| C10 | mixed | ND | 58.4203 | 27.03095 | 2017-04-18 |
| A10 | mixed | ND | 58.41071 | 26.64834 | 2017-04-18 |

| | | | | | |
|---|---|---|---|---|---|
| A7 | mixed | ND | 58.41531 | 27.01135 | 2017-04-18 |
| A8 | mixed | ND | 58.34459 | 26.88742 | 2017-04-18 |
| A11 | mixed | ND | 58.54372 | 27.17655 | 2017-04-20 |
| A12 | mixed | ND | 58.57232 | 26.97972 | 2017-04-20 |
| C9 | mixed | ND | 58.57532 | 27.00387 | 2017-04-20 |
| D5 | mixed | ND | 58.57557 | 26.93348 | 2017-04-20 |
| A14 | mixed | ND | 58.68143 | 25.94158 | 2017-04-26 |
| E7 | mixed | ND | 58.69349 | 25.88983 | 2017-04-26 |
| A13 | mixed | ND | 58.7923 | 26.69756 | 2017-04-27 |
| A6 | mixed | ND | 58.74837 | 26.66048 | 2017-04-27 |
| B8 | mixed | ND | 58.78949 | 26.69286 | 2017-04-27 |
| D13 | mixed | ND | 58.78722 | 26.69229 | 2017-04-27 |
| C16 | mixed | ND | 58.70963 | 26.24122 | 2017-04-28 |
| E6 | mixed | ND | 58.67396 | 26.21472 | 2017-04-28 |
| F13 | mixed | ND | 58.5894 | 26.87087 | 2017-04-28 |
| A9 | mixed | ND | 58.5637 | 26.84393 | 2017-04-28 |
| B10 | mixed | ND | 58.78356 | 26.35436 | 2017-05-02 |
| A25 | mixed | ND | 58.78243 | 26.34949 | 2017-05-02 |
| A28 | mixed | ND | 58.77627 | 26.36268 | 2017-05-02 |
| C14 | mixed | ND | 58.78302 | 26.35552 | 2017-05-02 |
| C7 | mixed | ND | 58.67339 | 26.09781 | 2017-05-02 |
| D6 | mixed | ND | 58.76681 | 26.31774 | 2017-05-02 |
| DD8 | mixed | ND | 58.16012 | 26.41543 | 2017-05-03 |
| CC8 | mixed | ND | 58.15813 | 26.41632 | 2017-05-03 |
| CC9 | mixed | ND | 58.09822 | 26.53882 | 2017-05-05 |
| FF8 | mixed | ND | 58.09434 | 26.53175 | 2017-05-05 |
| G4920 | mixed | ND | 57.55581 | 26.54651 | 2017-05-05 |
| G4922 | mixed | ND | 57.57449 | 26.53306 | 2017-05-06 |
| G4921 | Alnus_incana | native | 57.56308 | 26.52815 | 2017-05-06 |
| S856 | mixed | ND | 57.89817 | 25.82583 | 2017-05-08 |
| S857 | mixed | ND | 57.8987 | 25.8255 | 2017-05-08 |
| EE9 | mixed | ND | 58.09002 | 26.52668 | 2017-05-08 |
| FF7 | mixed | ND | 58.17188 | 26.41842 | 2017-05-09 |
| EE10 | mixed | ND | 58.24932 | 26.45421 | 2017-05-10 |
| C12 | mixed | ND | 58.73648 | 26.50024 | 2017-05-11 |
| EE12 | mixed | ND | 58.04135 | 26.40073 | 2017-05-12 |
| FF10 | mixed | ND | 58.50999 | 26.48083 | 2017-05-15 |
| DD9 | mixed | ND | 58.09205 | 26.52745 | 2017-05-16 |
| DD10 | mixed | ND | 58.2835 | 27.34217 | 2017-05-17 |
| BB2 | mixed | ND | 58.12368 | 26.41898 | 2017-05-21 |
| CC10 | mixed | ND | 58.4132 | 26.55914 | 2017-05-22 |
| FF13 | mixed | ND | 58.41766 | 26.55758 | 2017-05-22 |
| HB4 | mixed | ND | 58.32631 | 26.55543 | 2017-05-24 |
| HB26 | mixed | ND | 58.25782 | 27.26744 | 2017-05-25 |
| HB29 | mixed | ND | 58.24647 | 27.27207 | 2017-05-25 |
| HB34 | mixed | ND | 58.25281 | 27.28706 | 2017-05-25 |

| | | | | | |
|---|---|---|---|---|---|
| EE15 | mixed | ND | 58.2819 | 27.32479 | 2017-05-26 |
| FF9 | mixed | ND | 58.27875 | 27.32374 | 2017-05-26 |
| A27 | mixed | ND | 58.27492 | 26.38845 | 2017-05-30 |
| A15 | mixed | ND | 58.1996 | 26.2109 | 2017-05-30 |
| A17 | mixed | ND | 58.25622 | 26.4417 | 2017-05-30 |
| A18 | mixed | ND | 58.24807 | 26.43623 | 2017-05-30 |
| A26 | mixed | ND | 58.28179 | 26.37571 | 2017-05-30 |
| A31 | mixed | ND | 58.171 | 26.1442 | 2017-05-30 |
| F15 | mixed | ND | 58.19204 | 26.41113 | 2017-05-30 |
| C17 | mixed | ND | 58.30883 | 26.50111 | 2017-05-30 |
| E13 | mixed | ND | 58.30324 | 26.66952 | 2017-05-31 |
| HB10 | mixed | ND | 58.504 | 26.83705 | 2017-05-31 |
| D15 | mixed | ND | 58.28387 | 26.63609 | 2017-05-31 |
| HB1 | mixed | ND | 58.27733 | 26.66323 | 2017-05-31 |
| F14 | mixed | ND | 58.23053 | 26.70184 | 2017-05-31 |
| HB7 | mixed | ND | 58.50096 | 26.90613 | 2017-05-31 |
| A20 | mixed | ND | 58.06977 | 26.75456 | 2017-06-01 |
| B13 | mixed | ND | 58.07034 | 26.7497 | 2017-06-01 |
| D8 | mixed | ND | 58.07592 | 26.7486 | 2017-06-01 |
| E8 | mixed | ND | 58.06969 | 26.75013 | 2017-06-01 |
| F3 | mixed | ND | 58.05183 | 26.76819 | 2017-06-01 |
| C11 | mixed | ND | 58.06976 | 26.74647 | 2017-06-01 |
| B9 | mixed | ND | 57.93885 | 26.88825 | 2017-06-02 |
| B12 | mixed | ND | 57.91856 | 27.03679 | 2017-06-02 |
| HB3 | mixed | ND | 57.97129 | 26.49369 | 2017-06-02 |
| A22 | mixed | ND | 57.91848 | 27.0302 | 2017-06-02 |
| HB11 | mixed | ND | 58.18968 | 26.30146 | 2017-06-02 |
| HB12 | mixed | ND | 57.87478 | 26.10402 | 2017-06-02 |
| HB2 | mixed | ND | 57.76839 | 26.26625 | 2017-06-02 |
| A16 | mixed | ND | 57.9331 | 26.91635 | 2017-06-02 |
| DD14 | mixed | ND | 58.42041 | 26.56047 | 2017-06-02 |
| A23 | mixed | ND | 57.9295 | 26.90651 | 2017-06-02 |
| HB15 | mixed | ND | 57.90624 | 26.10429 | 2017-06-02 |
| HB9 | mixed | ND | 57.75243 | 26.25518 | 2017-06-02 |
| A19 | mixed | ND | 57.93822 | 27.05667 | 2017-06-02 |
| A21 | Picea_abies | native | 57.95081 | 26.90061 | 2017-06-02 |
| HB8 | mixed | ND | 58.59067 | 25.24696 | 2017-06-05 |
| HB19 | mixed | ND | 58.82747 | 26.37799 | 2017-06-05 |
| HB20 | mixed | ND | 58.69669 | 26.32953 | 2017-06-05 |
| HB18 | Populus_x_wettsteinii | introduced | 58.72968 | 25.34749 | 2017-06-05 |
| D14 | mixed | ND | 58.04161 | 26.38288 | 2017-06-06 |
| HB16 | mixed | ND | 58.23227 | 26.95801 | 2017-06-06 |
| E14 | mixed | ND | 58.04124 | 26.38788 | 2017-06-06 |
| EE13 | mixed | ND | 58.71877 | 26.26459 | 2017-06-06 |
| FF12 | mixed | ND | 58.74159 | 26.31976 | 2017-06-06 |
| D12 | mixed | ND | 57.99788 | 26.62186 | 2017-06-06 |

| | | | | | |
|---|---|---|---|---|---|
| HB13 | mixed | ND | 57.87069 | 27.24765 | 2017-06-06 |
| HB5 | mixed | ND | 58.17925 | 27.40557 | 2017-06-06 |
| E10 | mixed | ND | 57.99184 | 26.62243 | 2017-06-06 |
| E11 | mixed | ND | 58.02826 | 26.47319 | 2017-06-06 |
| E9 | mixed | ND | 57.95532 | 26.06582 | 2017-06-06 |
| HB14 | mixed | ND | 58.23176 | 27.31327 | 2017-06-06 |
| HB6 | mixed | ND | 58.11858 | 27.20699 | 2017-06-06 |
| HB17 | Populus_x_wettsteinii | introduced | 57.55518 | 26.65993 | 2017-06-06 |
| B11 | mixed | ND | 57.87311 | 26.12841 | 2017-06-07 |
| D10 | mixed | ND | 57.87415 | 26.11871 | 2017-06-07 |
| C13 | mixed | ND | 57.92724 | 26.05453 | 2017-06-07 |
| D9 | mixed | ND | 57.92895 | 26.05501 | 2017-06-07 |
| E12 | mixed | ND | 57.96056 | 26.76525 | 2017-06-07 |
| HB25 | mixed | ND | 58.24587 | 27.28204 | 2017-06-07 |
| HB35 | mixed | ND | 58.26926 | 27.28733 | 2017-06-07 |
| D11 | mixed | ND | 57.96177 | 26.76475 | 2017-06-07 |
| HB28 | mixed | ND | 58.28555 | 27.27409 | 2017-06-07 |
| HB37 | mixed | ND | 58.2643 | 27.27915 | 2017-06-07 |
| F7 | mixed | ND | 57.88336 | 26.57601 | 2017-06-07 |
| HB21 | mixed | ND | 58.26672 | 27.29059 | 2017-06-07 |
| HB30 | mixed | ND | 58.26216 | 27.28799 | 2017-06-07 |
| C15 | mixed | ND | 58.10949 | 26.584 | 2017-06-08 |
| A24 | mixed | ND | 57.9711 | 26.06014 | 2017-06-08 |
| DD11 | mixed | ND | 58.08949 | 26.45329 | 2017-06-08 |
| F5 | mixed | ND | 57.98786 | 26.37913 | 2017-06-08 |
| CC11 | mixed | ND | 58.09317 | 26.43862 | 2017-06-08 |
| F9 | mixed | ND | 57.84144 | 26.79959 | 2017-06-08 |
| F10 | mixed | ND | 58.34545 | 26.89262 | 2017-06-09 |
| F12 | mixed | ND | 58.11084 | 26.97941 | 2017-06-09 |
| F8 | mixed | ND | 58.0918 | 27.38157 | 2017-06-09 |
| HB24 | mixed | ND | 58.27526 | 27.32656 | 2017-06-09 |
| F11 | mixed | ND | 58.18046 | 27.36125 | 2017-06-09 |
| F6 | mixed | ND | 58.13838 | 26.95376 | 2017-06-09 |
| HB22 | mixed | ND | 58.27248 | 27.31448 | 2017-06-09 |
| HB32 | mixed | ND | 58.29836 | 27.30226 | 2017-06-09 |
| HB36 | mixed | ND | 58.28023 | 27.33497 | 2017-06-09 |
| HB38 | mixed | ND | 58.27571 | 27.3265 | 2017-06-09 |
| HB39 | mixed | ND | 58.27363 | 27.32366 | 2017-06-09 |
| HB27 | mixed | ND | 58.28379 | 27.31558 | 2017-06-13 |
| HB23 | mixed | ND | 58.28396 | 27.31331 | 2017-06-13 |
| CC12 | mixed | ND | 58.25836 | 27.26955 | 2017-06-14 |
| HB31 | mixed | ND | 58.25597 | 27.29335 | 2017-06-14 |
| HB33 | mixed | ND | 58.27065 | 27.32643 | 2017-06-14 |
| HB40 | mixed | ND | 58.2603 | 27.30493 | 2017-06-14 |
| CC14 | mixed | ND | 58.74565 | 26.28129 | 2017-06-15 |
| DD12 | mixed | ND | 58.68535 | 26.25072 | 2017-06-15 |

| | | | | | |
|---|---|---|---|---|---|
| EE11 | mixed | ND | 58.40573 | 26.56406 | 2017-06-19 |
| CC3 | mixed | ND | 58.11203 | 26.39858 | 2017-06-21 |
| DD4 | mixed | ND | 58.11081 | 26.40151 | 2017-06-21 |
| DD5 | mixed | ND | 58.08945 | 26.45323 | 2017-06-21 |
| BB5 | mixed | ND | 58.29945 | 26.65185 | 2017-06-21 |
| EE4 | mixed | ND | 58.12182 | 26.48646 | 2017-06-21 |
| EE5 | mixed | ND | 58.11503 | 26.41307 | 2017-06-21 |
| FF3 | mixed | ND | 58.0898 | 26.44714 | 2017-06-21 |
| FF6 | mixed | ND | 58.5502 | 26.92683 | 2017-06-22 |
| CC7 | mixed | ND | 58.5482 | 26.92938 | 2017-06-22 |
| DD6 | mixed | ND | 58.43674 | 26.73116 | 2017-06-22 |
| EE6 | mixed | ND | 58.54453 | 26.92458 | 2017-06-22 |
| EE7 | mixed | ND | 58.44002 | 26.72991 | 2017-06-22 |
| FF4 | mixed | ND | 58.4621 | 26.81921 | 2017-06-22 |
| BB11 | mixed | ND | 58.3431 | 27.1077 | 2017-06-26 |
| BB13 | mixed | ND | 58.35702 | 26.96298 | 2017-06-26 |
| BB4 | mixed | ND | 58.69171 | 25.95098 | 2017-06-26 |
| B15 | mixed | ND | 58.65938 | 26.11451 | 2017-06-26 |
| CC6 | mixed | ND | 58.69726 | 26.24349 | 2017-06-26 |
| FF15 | mixed | ND | 58.27591 | 27.32177 | 2017-06-26 |
| FF5 | mixed | ND | 58.74805 | 26.31946 | 2017-06-26 |
| BB14 | mixed | ND | 58.02064 | 26.82038 | 2017-06-27 |
| BB15 | mixed | ND | 57.98596 | 26.07059 | 2017-06-27 |
| BB7 | mixed | ND | 58.12728 | 26.62424 | 2017-06-27 |
| BB12 | mixed | ND | 57.9109 | 26.0991 | 2017-06-27 |
| BB6 | mixed | ND | 58.12211 | 26.61081 | 2017-06-27 |
| BB10 | mixed | ND | 58.12242 | 26.70506 | 2017-06-27 |
| E15 | mixed | ND | 57.98483 | 26.81494 | 2017-06-27 |
| DD7 | mixed | ND | 58.22426 | 26.27879 | 2017-06-27 |
| B16 | Picea_abies | native | 58.04818 | 26.82776 | 2017-06-27 |
| BB3 | mixed | ND | 57.68499 | 26.93114 | 2017-06-28 |
| B14 | mixed | ND | 57.99839 | 26.6065 | 2017-06-28 |
| BB9 | mixed | ND | 57.74994 | 26.92011 | 2017-06-28 |
| A32 | mixed | ND | 58.25067 | 27.29456 | 2017-06-28 |
| CC4 | mixed | ND | 57.71114 | 26.81106 | 2017-06-28 |
| CC5 | mixed | ND | 57.70343 | 26.74296 | 2017-06-28 |
| EE8 | mixed | ND | 57.77622 | 26.80449 | 2017-06-28 |
| BB8 | mixed | ND | 57.74928 | 26.92409 | 2017-06-28 |
| EE14 | mixed | ND | 58.4384 | 26.56969 | 2017-06-29 |
| DD13 | mixed | ND | 58.49476 | 26.49314 | 2017-07-04 |
| CC13 | mixed | ND | 58.48724 | 26.49684 | 2017-07-04 |
| CC15 | mixed | ND | 58.60082 | 26.98037 | 2017-07-05 |
| FF14 | Picea_abies | native | 58.61484 | 27.12781 | 2017-07-05 |
| G4925 | mixed | ND | 59.00101 | 24.62292 | 2017-07-10 |
| G4926 | mixed | ND | 59.00083 | 24.65429 | 2017-07-10 |
| G4924 | Salix_caprea | native | 59.2749 | 24.6893 | 2017-07-10 |

| | | | | | |
|---|---|---|---|---|---|
| DD15 | mixed | ND | 58.32543 | 26.5294 | 2017-07-17 |
| G4938 | Salix_alba | introduced | 57.8397 | 27.00099 | 2017-08-06 |
| G4939 | Picea_abies | native | 58.62724 | 23.43423 | 2017-08-18 |
| G4941 | mixed | ND | 58.69132 | 23.58581 | 2017-08-19 |
| G4942 | Corylus_avellana | native | 58.56215 | 23.55127 | 2017-08-19 |
| G4944 | mixed | ND | 58.64759 | 23.664 | 2017-08-20 |
| G4943 | Quercus_robur | native | 58.56145 | 23.72366 | 2017-08-20 |
| B7 | mixed | ND | 58.42666 | 26.56358 | 2017-10-10 |
| G4945 | Salix_caprea | native | 59.44022 | 25.15923 | 2017-10-20 |
| G4946 | mixed | ND | 59.35998 | 24.70673 | 2017-10-22 |
| E4 | mixed | ND | 58.16279 | 27.04277 | 2018-01-16 |
| S851 | mixed | ND | 59.21635 | 23.82291 | 2018-05-10 |
| S852 | mixed | ND | 59.21919 | 23.82316 | 2018-05-10 |
| S840 | Pinus_sylvestris | native | 59.21602 | 23.59926 | 2018-05-10 |
| S841 | Pinus_sylvestris | native | 59.17332 | 23.71756 | 2018-05-10 |
| S842 | Pinus_sylvestris | native | 59.1701 | 23.70469 | 2018-05-10 |
| S850 | Pinus_sylvestris | native | 59.21741 | 23.82181 | 2018-05-10 |
| S854 | Pinus_sylvestris | native | 59.18386 | 23.77166 | 2018-05-10 |
| A30 | mixed | ND | 58.74843 | 26.51465 | 2018-05-11 |
| S846 | mixed | ND | 59.23916 | 23.82582 | 2018-05-11 |
| S847 | mixed | ND | 59.20905 | 23.81343 | 2018-05-11 |
| S844 | Pinus_sylvestris | native | 59.23936 | 23.82707 | 2018-05-11 |
| S848 | Pinus_sylvestris | native | 59.20771 | 23.81091 | 2018-05-11 |
| G5002 | mixed | ND | 59.5753 | 24.5359 | 2018-05-19 |
| G5003 | Pinus_sylvestris | native | 59.5634 | 24.5451 | 2018-05-19 |
| G5050 | Salix_caprea | native | 58.36462 | 26.77559 | 2018-08-18 |
| Darta01 | mixed | ND | 57.36478 | 24.61866 | 2019-08-21 |
| Darta03 | mixed | ND | 56.55092 | 24.06976 | 2019-08-22 |
| Darta02 | mixed | ND | 56.56412 | 24.07548 | 2019-08-22 |
| G5158 | mixed | ND | 57.91265 | 27.31621 | 2019-08-24 |
| Darta04 | mixed | ND | 56.3629 | 24.3203 | 2019-08-26 |
| G5051 | mixed | ND | 58.43022 | 23.7414 | 2019-08-29 |
| G5054 | mixed | ND | 58.35534 | 26.52324 | 2019-09-02 |
| G5056 | Populus_berolinensis | introduced | 58.35775 | 26.52022 | 2019-09-02 |
| G5055 | Salix_viminalis | native | 58.35847 | 26.521 | 2019-09-02 |
| Darta06 | mixed | ND | 57.06564 | 22.76504 | 2019-09-06 |
| Darta05 | mixed | ND | 56.9193 | 22.69136 | 2019-09-06 |
| Darta07 | mixed | ND | 57.05855 | 22.71935 | 2019-09-06 |
| Darta08 | mixed | ND | 56.61266 | 23.15643 | 2019-09-10 |
| Darta09 | mixed | ND | 56.30974 | 25.45765 | 2019-09-12 |
| Darta10 | mixed | ND | 56.25794 | 25.42451 | 2019-09-12 |
| Darta18 | mixed | ND | 57.0369 | 22.67308 | 2019-09-18 |
| Darta11 | mixed | ND | 56.63219 | 24.35053 | 2019-09-19 |
| Darta12 | mixed | ND | 56.65789 | 24.33792 | 2019-09-19 |
| Darta14 | mixed | ND | 56.56941 | 24.14742 | 2019-09-20 |
| Darta13 | mixed | ND | 56.56989 | 24.14617 | 2019-09-20 |

| | | | | | |
|---|---|---|---|---|---|
| Darta16 | mixed | ND | 57.07765 | 22.68044 | 2019-09-23 |
| Darta15 | mixed | ND | 57.05434 | 22.69968 | 2019-09-23 |
| Darta17 | mixed | ND | 56.47827 | 24.1128 | 2019-09-27 |
| Darta19 | mixed | ND | 56.60674 | 22.85058 | 2019-10-02 |
| Darta20 | mixed | ND | 56.29552 | 27.28654 | 2019-10-03 |
| Darta21 | mixed | ND | 57.1905 | 21.90235 | 2019-10-04 |
| Darta22 | mixed | ND | 57.65579 | 26.09737 | 2019-10-07 |
| Darta23 | mixed | ND | 56.99967 | 23.42558 | 2019-10-08 |
| Darta27 | mixed | ND | 56.86395 | 24.78666 | 2019-10-09 |
| Darta29 | mixed | ND | 56.74038 | 24.93961 | 2019-10-09 |
| Darta28 | mixed | ND | 56.72302 | 22.99427 | 2019-10-10 |
| Darta24 | mixed | ND | 56.77181 | 22.59988 | 2019-10-10 |
| Darta25 | mixed | ND | 56.89941 | 25.18167 | 2019-10-11 |
| Darta26 | mixed | ND | 56.84068 | 25.39321 | 2019-10-11 |
| Darta30 | mixed | ND | 56.69142 | 25.20219 | 2019-10-14 |
| Darta31 | mixed | ND | 57.04657 | 25.07734 | 2019-10-14 |
| G5191 | Alnus_incana | native | 58.2113 | 26.5884 | 2020-05-29 |
| G5192 | Alnus_incana | native | 58.28489 | 26.7727 | 2020-06-01 |
| G5193 | mixed | ND | 58.4719 | 26.6808 | 2020-06-02 |
| G5194 | Alnus_incana | native | 58.4501 | 26.7073 | 2020-06-02 |
| G5195 | Larix_sibirica | introduced | 58.37389 | 26.57886 | 2020-06-02 |
| G5183 | mixed | ND | 58.36639 | 26.68209 | 2020-06-03 |
| G5197 | Alnus_incana | native | 58.1776 | 26.8035 | 2020-06-05 |
| G5205 | Quercus_robur | native | 59.5039 | 26.5339 | 2020-06-12 |
| G5206 | Corylus_avellana | native | 59.4978 | 26.6131 | 2020-06-12 |
| G5198 | Alnus_incana | native | 59.2348 | 26.2665 | 2020-06-12 |
| G5204 | Alnus_incana | native | 59.51699 | 26.5397 | 2020-06-12 |
| G5200 | Populus_tremula | native | 59.4845 | 26.4982 | 2020-06-12 |
| G5203 | Populus_balsamifera | introduced | 59.4989 | 26.5088 | 2020-06-12 |
| G5207 | Populus_x_wettsteinii | introduced | 59.4992 | 26.5792 | 2020-06-12 |
| G5199 | Salix_caprea | native | 59.4657 | 26.4831 | 2020-06-12 |
| G5201 | Salix_caprea | native | 59.5337 | 26.4682 | 2020-06-12 |
| G5202 | Salix_alba | introduced | 59.4991 | 26.5213 | 2020-06-12 |
| G5208 | Salix_alba | introduced | 59.4882 | 26.5376 | 2020-06-12 |
| G5209 | Larix_sibirica | introduced | 58.36088 | 26.68796 | 2020-06-17 |
| G5211 | Pinus_sylvestris | native | 58.18111 | 26.82206 | 2020-06-19 |
| G5218 | Alnus_glutinosa | native | 58.3605 | 25.6113 | 2020-06-21 |
| G5212 | Alnus_incana | native | 58.38357 | 25.6068 | 2020-06-21 |
| G5214 | Populus_balsamifera | introduced | 58.3805 | 25.59466 | 2020-06-21 |
| G5213 | Salix_alba | introduced | 58.37846 | 25.6003 | 2020-06-21 |
| G5217 | Salix_cinerea | native | 58.3572 | 25.5993 | 2020-06-21 |
| G5215 | Larix_sibirica | introduced | 58.3703 | 25.5949 | 2020-06-21 |
| G5216 | Pseudotsuga_menziesii | introduced | 58.3678 | 25.58716 | 2020-06-21 |
| G5222 | Alnus_glutinosa | native | 59.37195 | 24.04174 | 2020-06-23 |
| G5221 | Alnus_incana | native | 59.3409 | 24.20167 | 2020-06-23 |
| G5219 | Populus_tremula | native | 59.35455 | 24.5047 | 2020-06-23 |

| | | | | | |
|---|---|---|---|---|---|
| G5220 | Salix_caprea | native | 59.3304 | 24.351 | 2020-06-23 |
| G5225 | Alnus_glutinosa | native | 59.40425 | 24.7067 | 2020-06-24 |
| G5224 | Populus_berolinensis | introduced | 59.446 | 24.6861 | 2020-06-24 |
| G5223 | Salix_alba | introduced | 59.4152 | 24.7651 | 2020-06-24 |
| G5247 | Pseudotsuga_menziesii | introduced | 59.1057 | 26.2317 | 2020-06-30 |
| G5248 | Populus_balsamifera | introduced | 59.3052 | 26.3187 | 2020-07-01 |
| G5226 | Corylus_avellana | native | 57.9089 | 26.9936 | 2020-07-05 |
| G5227 | Larix_sibirica | introduced | 57.9812 | 26.8737 | 2020-07-05 |
| G5228 | Populus_balsamifera | introduced | 58.30835 | 26.58193 | 2020-07-12 |
| G5232 | Populus_tremula | native | 58.25424 | 26.8855 | 2020-07-13 |
| G5229 | Populus_berolinensis | introduced | 58.3891 | 26.7173 | 2020-07-13 |
| G5231 | Populus_berolinensis | introduced | 58.2411 | 26.7032 | 2020-07-13 |
| G5230 | Salix_pentandra | native | 58.3574 | 26.7373 | 2020-07-13 |
| G5187 | mixed | ND | 58.4206 | 26.6595 | 2020-07-16 |
| G5236 | Populus_tremula | native | 58.39394 | 26.79551 | 2020-07-16 |
| G5233 | Populus_balsamifera | introduced | 58.3972 | 26.7693 | 2020-07-16 |
| G5235 | Larix_sibirica | introduced | 58.3835 | 26.7742 | 2020-07-16 |
| G5234 | Pseudotsuga_menziesii | introduced | 58.3843 | 26.7744 | 2020-07-16 |
| G5241 | Populus_alba | introduced | 58.74266 | 25.80972 | 2020-07-18 |
| G5238 | Salix_fragilis | introduced | 58.6532 | 26.0082 | 2020-07-18 |
| G5237 | Larix_sibirica | introduced | 58.6109 | 26.1956 | 2020-07-18 |
| G5240 | Larix_sibirica | introduced | 58.6612 | 25.9695 | 2020-07-18 |
| G5239 | Pseudotsuga_menziesii | introduced | 58.6599 | 25.9695 | 2020-07-18 |
| G5243 | Populus_berolinensis | introduced | 58.36588 | 26.84505 | 2020-07-23 |
| G5242 | Populus_balsamifera | introduced | 58.09161 | 27.08756 | 2020-07-23 |
| G5244 | Populus_berolinensis | introduced | 58.46515 | 26.71431 | 2020-07-24 |
| G5246 | Populus_balsamifera | introduced | 58.35488 | 26.61914 | 2020-07-26 |
| G5245 | Populus_x_wettsteinii | introduced | 58.23347 | 27.30861 | 2020-07-26 |
| G5250 | Populus_tremula | native | 58.31888 | 25.53064 | 2020-07-31 |
| G5251 | Corylus_avellana | native | 59.41437 | 27.67419 | 2020-08-01 |
| G5254 | Populus_balsamifera | introduced | 59.40758 | 27.11273 | 2020-08-01 |
| G5256 | Populus_balsamifera | introduced | 59.40011 | 28.07172 | 2020-08-01 |
| G5252 | Populus_alba | introduced | 59.38811 | 27.78241 | 2020-08-01 |
| G5255 | Salix_pentandra | native | 59.39218 | 28.19321 | 2020-08-01 |
| G5253 | Larix_sibirica | introduced | 59.38419 | 27.35992 | 2020-08-01 |
| G5257 | Salix_pentandra | native | 58.36355 | 26.38814 | 2020-08-03 |
| G5258 | Picea_pungens | introduced | 58.35475 | 26.6845 | 2020-08-10 |
| G5262 | Populus_balsamifera | introduced | 58.5848 | 26.69838 | 2020-08-14 |
| G5260 | Picea_omorika | introduced | 58.63459 | 26.38551 | 2020-08-14 |
| G5261 | Pinus_nigra | introduced | 58.64096 | 26.38468 | 2020-08-14 |
| G5259 | Larix_sibirica | introduced | 58.6339 | 26.38555 | 2020-08-14 |
| G5263 | Picea_pungens | introduced | 58.05234 | 27.04099 | 2020-08-15 |
| G5265 | Populus_tremula | native | 59.34881 | 26.26735 | 2020-08-18 |
| G5264 | Salix_fragilis | introduced | 59.35277 | 26.34935 | 2020-08-18 |
| G5266 | Salix_alba | introduced | 59.3491 | 26.1102 | 2020-08-18 |
| G5267 | Picea_pungens | introduced | 59.43404 | 24.85862 | 2020-08-20 |

| | | | | | |
|---|---|---|---|---|---|
| G5270 | Betula_pendula | native | 58.9089 | 24.90225 | 2020-08-26 |
| G5268 | Populus_berolinensis | introduced | 58.77773 | 25.42577 | 2020-08-26 |
| G5271 | Salix_caprea | native | 58.90706 | 24.90023 | 2020-08-26 |
| G5269 | Larix_sibirica | introduced | 58.86771 | 24.52982 | 2020-08-26 |
| G5282 | Quercus_robur | native | 58.28188 | 27.05216 | 2020-08-30 |
| G5278 | Quercus_rubra | introduced | 58.28449 | 27.28438 | 2020-08-30 |
| G5277 | Populus_x_wettsteinii | introduced | 58.28429 | 27.28327 | 2020-08-30 |
| G5272 | Picea_omorika | introduced | 58.28456 | 27.28768 | 2020-08-30 |
| G5273 | Pinus_sylvestris | native | 58.28311 | 27.28654 | 2020-08-30 |
| G5276 | Pinus_contorta | introduced | 58.28396 | 27.28492 | 2020-08-30 |
| G5420 | Pinus_koraiensis | introduced | 58.28381 | 27.28559 | 2020-08-30 |
| G5274 | Larix_sibirica | introduced | 58.28386 | 27.28612 | 2020-08-30 |
| G5281 | Pseudotsuga_menziesii | introduced | 58.28476 | 27.28656 | 2020-08-30 |
| G5279 | Abies_sachalinensis | introduced | 58.2847 | 27.28436 | 2020-08-30 |
| G5275 | Abies_veitchii | introduced | 58.28381 | 27.28495 | 2020-08-30 |
| G5280 | Abies_holophylla | introduced | 58.28454 | 27.2855 | 2020-08-30 |
| G5283 | Quercus_robur | native | 58.38449 | 26.5943 | 2020-08-31 |
| G5284 | Populus_nigra | introduced | 58.37531 | 26.67882 | 2020-08-31 |
| G5421 | Pinus_nigra | introduced | 58.39132 | 26.69648 | 2020-09-02 |
| G5422 | Pinus_strobus | introduced | 58.39093 | 26.69732 | 2020-09-02 |
| G5423 | Abies_sp | introduced | 58.39312 | 26.69297 | 2020-09-02 |
| G5291 | Quercus_robur | native | 56.86893 | 24.35352 | 2020-09-03 |
| G5426 | Quercus_rubra | introduced | 56.8663 | 24.3526 | 2020-09-03 |
| G5289 | Corylus_avellana | native | 56.86743 | 24.36013 | 2020-09-03 |
| G5292 | Populus_x_wettsteinii | introduced | 56.87014 | 24.35424 | 2020-09-03 |
| G5285 | Populus_sp | introduced | 57.55197 | 25.43747 | 2020-09-03 |
| G5287 | Populus_sp | introduced | 57.03912 | 24.39745 | 2020-09-03 |
| G5288 | Populus_sp | introduced | 56.86902 | 24.36231 | 2020-09-03 |
| G5286 | Salix_fragilis | introduced | 57.26109 | 24.88131 | 2020-09-03 |
| G5424 | Picea_mariana | introduced | 57.18122 | 24.69495 | 2020-09-03 |
| G5427 | Picea_mariana | introduced | 56.87097 | 24.35989 | 2020-09-03 |
| G5293 | Larix_sibirica | introduced | 56.87127 | 24.36017 | 2020-09-03 |
| G5290 | Pseudotsuga_menziesii | introduced | 56.8662 | 24.3542 | 2020-09-03 |
| G5294 | Pinus_mugo | introduced | 58.37468 | 26.74911 | 2020-09-27 |
| G5295 | Pinus_mugo | introduced | 58.3592 | 26.6443 | 2020-09-27 |
| S1381 | mixed | ND | 56.64042 | 24.69382 | 2020-11-04 |
| S1384 | mixed | ND | 57.20478 | 25.20654 | 2020-11-06 |
| S1386 | mixed | ND | 57.19698 | 25.20423 | 2020-11-06 |
| S1388 | mixed | ND | 56.99859 | 23.17395 | 2020-11-10 |
| S1387 | mixed | ND | 57.00299 | 23.14729 | 2020-11-10 |
| S1390 | mixed | ND | 56.77796 | 25.43266 | 2020-11-11 |
| S1392 | mixed | ND | 56.76726 | 25.4353 | 2020-11-11 |
| S1393 | mixed | ND | 56.5908 | 23.77725 | 2020-11-12 |
| S1394 | mixed | ND | 56.59206 | 23.78062 | 2020-11-12 |

[1]ND, not determined
