# Supplementary material for: The influence of tree genus, phylogeny, and richness on the specificity, rarity, and diversity of ectomycorrhizal fungi
Source: Environ Microbiol Rep. 2024 Apr 4;16(2):e13253. doi: 10.1111/1758-2229.13253 (PMC10994715; doi:10.1111/1758-2229.13253)
Supplement: Supplementary file 18 — TABLE S3. Best generalized mixed models for diversity parameters of fungi. [file EMI4-16-e13253-s006.pdf]

Table S3 Best generalized mixed models for diversity parameters of fungi.

	SS	DF	F-value	R^2	*P*-value
**Richness of EcM fungi**					
EcM tree genus	19.0	10	14.3	0.177	<0.001
soil pH	13.9	2	71.0	0.129	<0.001
year of sampling	10.0	8	27.0	0.093	<0.001
EcM plant basal area (%)	7.3	1	49.2	0.068	<0.001
soil $\delta^{15}N$	3.8	1	44.6	0.035	<0.001
richness of EcM plants	1.5	1	12.0	0.015	0.003
error	51.8	1257			
**Richness of non-ectomycorrhizal fungi**					
EcM plant basal area (%)	5.1	1	387.7	0.184	<0.001
year of sampling	3.7	8	31.5	0.134	<0.001
EcM tree genus	1.3	10	10.7	0.046	<0.001
soil pH	0.8	2	61.4	0.029	<0.001
richness of woody plants	0.3	1	19.5	0.009	<0.001
vegetation age	0.1	1	11.1	0.005	<0.001
error	17.4	1248			
**Richness of all fungi**					
vegetation age	2.3	1	13.8	0.141	<0.001
soil pH	2.0	2	83.5	0.123	<0.001
year of sampling	1.7	8	28.0	0.104	<0.001
tree genus	1.4	10	6.1	0.085	<0.001
EcM plant basal area (%)	0.4	1	17.1	0.024	<0.001
error	8.4	1268			
**Richness of EcM fungal lineages**					
EcM tree genus	4.7	10	37.7	0.206	<0.001
soil pH	2.1	2	188.3	0.094	<0.001
EcM plant basal area (%)	0.9	1	78.9	0.039	<0.001
vegetation age	0.7	1	61.8	0.031	<0.001
error	14.2	1267			
**Richness of rare EcM fungi**					
richness of all EcM fungi	99.0	1	2012.1	0.564	<0.001
richness of EcM plants	7.7	1	156.9	0.043	<0.001
EcM plant basal area (%)	6.6	1	133.8	0.037	<0.001
error	62.3	1266			
**Proportion of rare EcM fungal species**					
soil pH	1.3	2	278.9	14.5	<0.001
richness of all EcM fungi	1.0	1	219.3	11.4	<0.001
EcM tree genus	0.7	10	18.1	8.0	<0.001
error	5.7	1257			
**Relative abundance of EcM fungi**					
year of sampling	38.3	8	3.3	0.112	<0.001
month of sampling	13.8	7	1.0	0.040	0.420
year x month of sampling	35.7	34	6.5	0.105	<0.001
EcM plant basal area (%)	30.0	1	49.9	0.088	<0.001
soil pH	24.7	1	13.1	0.072	<0.001
EcM tree genus	23.3	10	4.1	0.068	0.002
soil P concentration	6.6	1	17.1	0.019	<0.001
error	168.6	1221			
