# Supplementary material for: The influence of tree genus, phylogeny, and richness on the specificity, rarity, and diversity of ectomycorrhizal fungi
Source: Environ Microbiol Rep. 2024 Apr 4;16(2):e13253. doi: 10.1111/1758-2229.13253 (PMC10994715; doi:10.1111/1758-2229.13253)
Supplement: Supplementary file 19 — TABLE S4. Best models for Φmax,ave and ΦW values. [file EMI4-16-e13253-s009.pdf]

TABLE S4 Best models for $\Phi_{max,ave}$ and Φ^W values.

	SS	DF	F-value	R^2	*P*-value
**$\Phi_{max,ave}$ values**					
EcM tree genus	0.71	9	82.2	0.371	<0.001
soil pH	0.10	2	147.9	0.074	<0.001
error	1.00	1106			
**Φ^W values**					
EcM tree genus	2.51	9	80.5	0.357	<0.001
soil pH	0.50	2	145.7	0.072	<0.001
soil $\delta^{15}N$	0.21	1	54.8	0.027	<0.001
error	3.80	1105			
